# Targeting the gut-ovarian axis: *Scutellaria baicalensis* improves polycystic ovary syndrome by modulating gut microbiota composition and inhibiting the *LPS/TLR4/NF-κB* signaling pathway

**DOI:** 10.1128/msystems.01825-25

**Published:** 2026-06-15

**Authors:** Zhengxiu Pan, Caili Zhang, Qiangqiang Chu, Mingwei Chen, Cancan Hui, Tianjuan Wang, Maozhen Han, Datong Deng

**Affiliations:** 1 Department of Endocrinology and Metabolism, The First Affiliated Hospital of Anhui Medical University, Hefei, China; 2 Endocrinology Department of He County Traditional Chinese Medicine Hospital, Ma'anshan, Anhui, China; 3 College of Life Sciences, Anhui Medical University, Hefei, China; 4 Anhui Tongling Bionic Technology Co., Ltd., Hefei, China; 5 Department of Geriatric Endocrinology, The First Affiliated Hospital of Anhui Medical University, Hefei, China; 6 Department of Reproductive Medicine, The First Affiliated Hospital of Anhui Medical University, Hefei, China; 7 Anhui Medical University, Institute of Endocrinology and Metabolism 12485, Hefei, China; 1 Saha Institute of Nuclear Physics, Kolkata, India; Saha Institute of Nuclear Physics, Kolkata, India

**Keywords:** polycystic ovary syndrome, *Scutellaria baicalensis*, gut microbiota, network pharmacology, molecular docking

## Abstract

Polycystic ovary syndrome (PCOS) is a chronic inflammatory disease caused by a combination of genetic, environmental, and gut microbiota factors. Western medicines can improve ovulation rates, but are linked with reduced pregnancy success and side effects. *Scutellaria baicalensis* (*BAL*) is a famous anti-inflammatory Chinese herb, which has therapeutic effects for PCOS; however, the mechanism remains unclear. This study integrated network pharmacology, molecular docking, and animal experiments to investigate the mechanisms by which *BAL* improves gut microbiota and intestinal inflammation in a rat PCOS model. We identified 36 active components of *BAL*, including *wogonin*, *baicalein*, and *oroxylin A*, along with 145 potential targets, such as *TNF*, *IL-6*, and *AKT1*, and network analysis revealed that *TNF*, *IL-6*, and *AKT1* occupied central positions. Enrichment analyses indicated that *BAL* may exert its therapeutic effects by modulating the *NF-κB* and Toll-like receptor signaling pathways. Molecular docking results demonstrated strong binding affinities between *BAL*’s primary active components and the core targets *TNF*, *AKT1*, and *IL-6. BAL* experiments in PCOS rats showed that it restored gut microbiota balance. These results showed that *BAL* inhibits the *LPS/TLR4/NF-κB* pathway and downregulates PI3K and p-Akt/Akt protein expression in ovarian tissue. *BAL* also improved reproductive metabolic abnormalities, intestinal inflammation, and dysbiosis. Our study suggests that *BAL* inhibits the *LPS/TLR4/NF-κB* pathway via the gut-ovarian axis, regulating gut microbial composition and alleviating inflammatory states. This may be a mechanism for the therapeutic efficacy of *BAL* for PCOS and a novel therapeutic strategy for the clinical management of polycystic ovary syndrome.

**IMPORTANCE:**

PCOS affects women of reproductive age. Current clinical treatment strategies still face limitations in efficacy and long-term side effects. This study employs network pharmacology, molecular docking, and experimental validation to elucidate the mechanism by which the traditional Chinese medicine *BAL* improves PCOS. We found that *BAL* modulates gut microbiota in a PCOS rat model, significantly increasing the probiotic genus *Phocaeicola*. This change correlates with reduced serum LPS levels and alleviated ovarian inflammation. Experiments confirm that *BAL* alleviates local ovarian inflammation and insulin resistance by inhibiting the *LPS/TLR4/NF-κB* signaling pathway, thereby improving PCOS pathological manifestations. This study reveals new mechanisms of *BAL* in treating PCOS and highlights the potential bridging role of probiotics like *Phocaeicola*. It also provides experimental evidence and theoretical support for integrated Chinese and Western medicine treatment strategies based on regulating gut microbiota.

## INTRODUCTION

Polycystic ovary syndrome (PCOS) is a highly prevalent endocrine disorder among women, particularly common in those of childbearing age (1). It was first identified in 1935 by Stein and colleagues and is also known as hyperandrogenic anovulation (HA) or the Stein-Leventhal syndrome (2, 3). Approximately 5–20% of women of childbearing age worldwide are affected by it (4). A characteristic manifestation of this condition is the presence of elevated levels of androgen hormones in conjunction with chronic anovulation, primarily manifested as irregular menstrual cycles, infrequent or absent ovulation, hyperandrogenism, and polycystic ovarian changes (5, 6). The Rotterdam criteria are typically used to make a diagnosis, and these require the presence of at least two of the following: oligomenorrhea or amenorrhea, clinical or biochemical evidence of hyperandrogenism, and ultrasound detection of polycystic ovarian morphology (7). PCOS can lead to infertility, obesity, hyperandrogenism, and long-term complications such as type 2 diabetes, cardiovascular disease, metabolic syndrome, and endometrial cancer. Additionally, PCOS increases the risk of complications such as preeclampsia, gestational diabetes, and miscarriage, posing a significant burden on women’s health (8, 9). Health and Human Development (NICHD) claimed that PCOS is one of the leading causes of infertility in women of reproductive age (10).

In recent years, numerous studies have identified various etiologies of PCOS, including genetic factors, environmental factors, and gut microbiota dysbiosis. However, due to the complexity and heterogeneity of the disease, the precise pathogenesis of PCOS remains unclear (11, 12). In particular, many PCOS patients obtain poor clinical outcomes. Therefore, further in-depth research to elucidate the pathogenesis of PCOS is crucial (13). Currently, the primary goal of PCOS treatment is to alleviate its symptoms. This includes using combined oral contraceptives (COCs), metformin, and ovulation-inducing medications to address irregular menstruation, insulin resistance, hyperandrogenism, and infertility (9, 14). However, these treatments typically increase ovulation rates but decrease pregnancy rates, leading to multiple adverse consequences including abnormal uterine bleeding, ovarian hyperstimulation syndrome, premature ovarian failure, venous thromboembolism, and severe gastrointestinal adverse reactions.

Therefore, while striving to develop safe and effective drugs for PCOS, it is essential to actively seek safe and effective treatment strategies from traditional Chinese medicine (15, 16). Certain phytochemicals derived from traditional Chinese medicinal herbs show promise in improving various PCOS-related symptoms, including insulin resistance, hyperinsulinemia, hyperandrogenism, ovarian dysfunction, obesity, miscarriage, and infertility (17). *Scutellaria baicalensis (BAL*) belongs to the *Scutellaria* genus within the *Lamiaceae* family and is recognized for its traditional use in herbal medicine, particularly in East Asian countries. *BAL* possesses potent anti-inflammatory, anti-cancer, and antiviral properties (18, 19). PCOS is a chronic inflammatory disease; improving chronic inflammation can alleviate PCOS (20). However, there is limited research on whether *BAL* alleviates reproductive and metabolic disorders associated with PCOS through anti-inflammatory mechanisms. Network pharmacology, a pioneering discipline grounded in systems biology and network analysis, enables the investigation of drug-disease correlations at the molecular level (21). Predicting the active components, core targets, and mechanisms of action of *BAL* in treating PCOS, molecular docking can elucidate interactions between *BAL* and its target proteins, reflecting the stability of docking complexes between *BAL*’s active components and core targets (22). To provide a research foundation and insights for further experimental validation, guiding the development and application of drugs.

Recent studies suggest a potential association between gut microbiota and PCOS (23). Gut microbiota may contribute to the development of PCOS through multiple mechanisms, including regulating the immune system, modulating sex hormones, and releasing inflammatory factors. These pathways exert adverse effects on reproductive and metabolic functions, leading to alterations in sex hormone levels and metabolic dysfunction. Consequently, this may trigger polycystic ovary syndrome, infertility, and various metabolic disorders (8, 24). Although gut microbiota is associated with PCOS, little is known about whether *BAL* improves PCOS reproductive and metabolic disorders by regulating gut microbiota dysbiosis. The relationship between the pathogenesis and pathophysiological processes of PCOS and the structure and function of gut microbiota requires further investigation.

This study employed an integrated approach combining network pharmacology and molecular docking to systematically investigate the potential targets and mechanisms of *BAL* (whose primary active components include *baicalein*, *wogonin*, and others) in the treatment of PCOS. Using the TCMSP, HERB, and UniProt databases, we identified the active components of *BAL* and their corresponding targets. Key active compounds, such as *wogonin*, *baicalein*, and *oroxylin A*, were identified, with core targets including *TNF*, *IL-6*, *AKT1*, and *TP53*. GO and KEGG enrichment analyses indicated that *BAL* may exert therapeutic effects by modulating the *NF-κB*, *PI3K-Akt*, Toll-like receptor, and *TNF* signaling pathways. Molecular docking results further demonstrated strong binding affinities between *wogonin*, *baicalein*, and *oroxylin A* and key targets such as *TNF*, *IL-6*, and *AKT1*. To further validate its mechanism, a letrozole-induced PCOS rat model was established. Groups included healthy controls (Con), PCOS model (Mod), *BAL* intervention (50 mg/kg/day), and metformin (Met) control (270 mg/kg/day), with continuous intervention for 5 weeks. Throughout the experiment, changes in rat body weight and estrous cycle were monitored to evaluate the effects of *BAL* on metabolic and reproductive endocrine functions. Glucose metabolism status was assessed via oral glucose tolerance test (OGTT), area under the curve (AUC), and insulin resistance index (HOMA-IR) using the steady-state model. Fecal samples were collected for metagenomic sequencing to evaluate gut microbiota composition changes. Ovarian and colon tissues were subjected to hematoxylin and eosin (H&E) staining to observe histopathological alterations; serum levels of neutral hormones, insulin, and inflammatory cytokines were measured via ELISA; and RT-qPCR was used to detect mRNA expression of colonic tight junction proteins (*Occludin*, *Claudin-1*, *ZO-1*) and ovarian inflammatory factors (*TNF-α*, *IL-6*, *IL-1β*, *IL-18*). Western blot analysis assessed protein expression of the *TLR4/PI3K/Akt/NF-κB p65* pathway in ovarian tissue. Results demonstrated that *BAL* intervention significantly suppressed weight gain in PCOS rats (*P <* 0.05), improved glucose metabolism disorders, and corrected reproductive endocrine abnormalities. Metagenomic analysis revealed that *BAL* significantly increased the abundance of the intestinal genus *Phocaeicola* (*P <* 0.01), repaired intestinal barrier function, and alleviated inflammatory responses. Mechanistically, *BAL* reduced serum LPS levels (*P <* 0.0001), improved insulin resistance and impaired glucose tolerance, markedly alleviated pathological damage in ovarian and colonic tissues, upregulated colonic tight junction protein expression, decreased ovarian inflammatory factor expression, and significantly suppressed key protein expression in the *TLR4/PI3K/Akt/NF-κB* pathway. Network pharmacology and molecular docking results further confirmed the *TLR4/NF-κB* pathway as the key mechanism underlying *BAL*’s effects. In summary, *BAL* may exert therapeutic effects on PCOS by modulating gut microbiota composition, increasing the abundance of the beneficial bacterium *Phocaeicola*, thereby inhibiting the *LPS/TLR4/NF-κB* signaling pathway, improving insulin resistance, reducing inflammatory responses, and mitigating ovarian tissue damage. *Phocaeicola* could serve as a potential biomarker reflecting *BAL*’s regulation of the intestinal microbiome.

The study employed an integrated multi-omics approach to systematically elucidate the mechanism by which *BAL* improves letrozole-induced PCOS. Through the integration of network pharmacology, molecular docking, molecular biology, and gut microbiota analysis, *BAL* was confirmed to possess therapeutic potential via multiple targets and pathways. Specifically, network pharmacology and molecular docking revealed interactions between *BAL*’s active components and key targets; histological experiments demonstrated its ability to regulate the expression of reproductive, metabolic, and inflammation-related genes; and gut microbiota analysis further confirmed *BAL*’s capacity to remodel the intestinal microbiome structure. Results indicate that *BAL* exerts its anti-PCOS effects by modulating reproductive-metabolic signaling pathways, improving ovarian gene expression networks, and restoring gut microbiota balance. This study provides a theoretical basis for the clinical application of *BAL* in treating PCOS.

## MATERIALS AND METHODS

**Fig 1:**
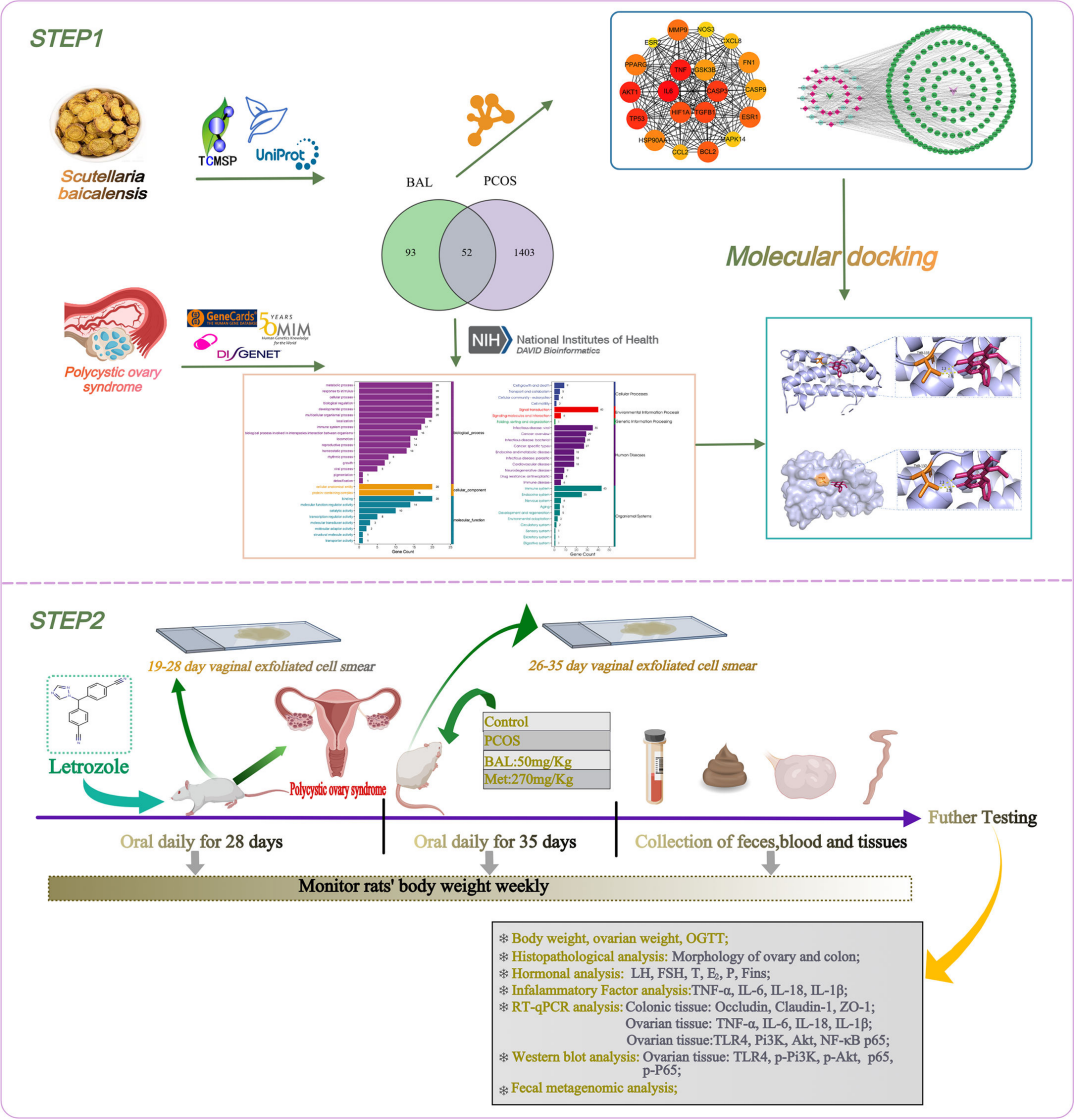
This study established an integrated research framework to elucidate the multi-target therapeutic mechanism of *Scutellaria baicalensis* in treating PCOS by combining network pharmacology, molecular docking, and animal experiments. This study employed an integrative strategy to explore the potential mechanisms of *BAL* in treating PCOS. A Venn diagram revealed 52 common interaction targets after 145 action targets of *BAL* from various databases. A “component-target” network was constructed to identify 20 core targets. A multidimensional network was established to reveal the association between *BAL* bioactive components (*baicalein*, *wogonin*, and *oroxylin A*) and PCOS pathology. Further analyses were performed using the DAVID platform, and the data were visualized online. Molecular docking results indicated that the three major *BAL* components exhibited good binding activity with core PCOS targets. These computational findings were experimentally validated in an animal model of PCOS. The 63-day experiment was divided into two phases: model establishment (28 days of letrozole intervention) and therapeutic intervention (35 days). Treatment groups received *BAL*, metformin, or saline. Evaluation parameters included biological, biochemical, and molecular indicators. Additionally, gut microbiota was analyzed via metagenomic sequencing. This study elucidates the mechanisms underlying *BAL*’s intervention in PCOS.

To investigate the molecular mechanisms underlying *BAL*’s treatment of PCOS, this study employed network pharmacology to predict its potential targets and pathways, followed by validation through *in vivo* experiments in rats (Fig. 1).

### Identification of active components and targets of *Scutellaria baicalensis*

The active ingredients of *BAL* are sourced from the TCMSP database (https://old.tcmsp-e.com/tcmsp.php) and the HERB database (http://herb.ac.cn/). Given that most traditional Chinese medicines are administered as oral decoctions, screening thresholds were set based on oral bioavailability (OB) ≥30% and drug-likeness (DL) ≥0.18 (25). The active components of *BAL* and their corresponding protein targets were identified. Using the UniProt database and restricting the target species to humans, the obtained protein targets were converted into their corresponding gene names. Protein targets without corresponding gene names were removed, thereby identifying the active components of *BAL* and their target proteins.

### Selection of targets for PCOS

PCOS targets were sourced from GeneCards (https://www.genecards.org/), DisGeNET (https://disgenet.com/), DrugBank (https://go.drugbank.com), and OMIM (https://www.omim.org/). Using “polycystic ovary syndrome” as the keyword, targets identified across these databases were consolidated and deduplicated using an Excel spreadsheet. This process yielded a comprehensive compilation of PCOS-related target information (26).

### Identification of common targets between *Scutellaria baicalensis* and PCOS

By utilizing an online platform (https://www.omicshare.com), we identified the intersection of *BAL*’s active ingredient targets and PCOS targets and plotted a Venn diagram, thereby obtaining potential targets for *BAL* treatment of PCOS.

### Protein-protein interaction (PPI) network construction and topological assessment

The 52 potential targets of *BAL* for treating PCOS were imported into the STRING database (https://cn.string-db.org/), specifying the species as “Homo sapiens” and the confidence level as “0.400.” Free nodes were hidden, while all other parameters were generated at their default settings to generate the PPI model. The TSV file was downloaded and imported into Cytoscape 3.10.3 for topological analysis. The top 20 key targets were calculated using the cytoHubba plugin. The CytoNCA plugin was employed to compute node degree values and generate a PPI network diagram for the top 20 key targets.

### Functional enrichment analysis of biological processes and signaling pathways

GO functional enrichment and KEGG pathway enrichment analyses were performed on potential targets of *BAL* treatment for PCOS using the DAVID database (https://davidbioinformatics.nih.gov/tools.jsp) (25). The obtained results were visualized by plotting bar charts using an online platform.

### Construction of the “drug-component-target-disease” network

Mapping the active components of *BAL* and their target molecules to diseases yielded intersecting target molecules. These were imported into Cytoscape 3.10.3 software to construct a “*Scutellaria baicalensis*-component-target-disease” network diagram. Software tools were employed to analyze and calculate network parameters, thereby illustrating the interaction relationships among the active components and intersecting target molecules.

### Molecular conformation validation

In the PPI network diagram, core targets were identified as receptors, and active components were selected as ligands for molecular docking validation. For details on the strategy for screening core targets and the workflow for identifying active compounds, please refer to the “Materials and Methods” section in the Supplemental materials. First, the 2D structures of *BAL* active components were obtained from the PubChem database in SDF format. The 2D structures of *BAL* active components were optimized using Chem3D software and converted to PDB format. Subsequently, hydrogen atoms were added to these components in AutodockTools software, and the files were exported in PDBQT format. Simultaneously, the corresponding ID for the core target was retrieved from the Unitprot database. The core target ID was entered into the PDB database to download the core target protein file. Using Pymol software, water molecules and existing ligands were removed from the file. The modified file was imported into AutodockTools for hydrogenation processing and exported in PDBQT format. The prepared receptor macromolecules and ligand small molecules were imported into AutodockTools to calculate their binding energies. Finally, docking diagrams of *BAL* key active ingredients with core target proteins were generated using Pymol for visual presentation.

### Animal experiment validation

#### Experimental design

This experiment utilized 50 female SD rats aged 4–5 weeks and weighing 130 ± 9.5 g (SPF grade, Liaoning Changsheng Biotechnology Co., Ltd., Certificate No.: 210726241100810247). The rats were housed in the SPF environment at 5–6 per cage with free access to food and water. The rats were maintained at a constant temperature (22–24°C) and humidity (60–70%) under a 12 h light/12 h dark cycle. After a 1-week acclimatization period, rats were randomly assigned to a blank control group (Con, *n* = 12) and a model group (Mod, *n* = 38). The Con group received daily oral administration of 1% CMC solution (1 mL/kg), while the Mod group received daily oral administration of letrozole dissolved in 1% CMC solution (1 mg/kg, 1 mL/kg) for 28 consecutive days to establish the PCOS model.

Starting from day 19 of modeling, vaginal smears were collected at a fixed time daily for 10 consecutive days. If rats exhibited persistent vaginal epithelial keratinization and estrous cycle disruption, preliminary modeling success was considered achieved. Following modeling completion, randomly select five rats from the Mod group and two from the Con group for ovarian H&E staining. If the Mod group ovaries exhibit increased cystic follicles, thinned and loosely arranged granulosa cell layers, and reduced numbers of corpus luteum and normal oocytes, the PCOS model is deemed successful.

Successfully modeled PCOS rats were randomly assigned to three groups (*n* = 11 per group): *BAL* group (*BAL*, 50 mg/kg/d), Met group (Met, 270 mg/kg/d dissolved in saline), and model control group (Mod, equivalent saline). A separate Con group (*n* = 10) received continuous saline gavage throughout the study. All groups underwent continuous intervention for 5 weeks with weekly weighing. Preliminary experiments suggested that the 50 mg/kg dose of *BAL* may be approaching the plateau phase of its effect; however, dose-gradient experiments ranging from 25 to 100 mg/kg are still required to determine the optimal dose for improving reproductive endocrine disorders in PCOS rats.

Starting on day 26 of treatment, vaginal smears were collected from all rats for 10 consecutive days. After Giemsa staining, the smears were examined under an optical microscope (400 × magnification). The estrous cycle phase was determined based on the proportions of keratinized epithelial cells, nucleated epithelial cells, and leukocytes to assess the drug’s regulatory effect on ovarian function.

After 5 weeks of treatment, fecal samples were collected from rats in each group, rapidly frozen in liquid nitrogen, and stored at –80°C. Body weight and fasting blood glucose levels were measured (blood drawn from the tail tip after 12-hour fasting and analyzed using a Roche blood glucose meter). At the end of the experiment, rats were anesthetized with 20% carbamide ethyl ester via intraperitoneal injection. Blood (5–10 mL) was collected from the abdominal aorta, centrifuged to obtain serum, and stored at –80°C. Following blood collection, rats were euthanized by cervical dislocation. Bilateral ovaries were dissected, weighed after fat removal, and preserved: the left ovary was fixed in 4% paraformaldehyde for histological analysis, while the right ovary was cryopreserved at –80°C for molecular testing. The spleen was dissected and isolated; the connective tissue was removed; the tissue was rinsed with PBS, blotted dry with filter paper, and weighed; and the spleen index was calculated [Spleen Index = Spleen wt (mg) / Body Weight (g)]. Concurrently, the entire colon was collected, with portions fixed and portions cryopreserved for subsequent analysis.

#### H&E staining and the oral glucose tolerance test (OGTT)

Colon and ovarian tissues were fixed, dehydrated, embedded, and sectioned. Sections were placed in a 60°C oven for 2 h to remove paraffin, followed by xylene dewaxing and hydration with a gradient of ethanol (100%–70%). Sections underwent routine H&E staining and neutral resin mounting before histopathological examination under a light microscope. Following drug treatment, rats were fasted for 12 h (with free access to water). Body weight was measured, and blood was collected via tail vein to determine fasting blood glucose (FBG). A 50% glucose solution was administered via gavage at a dose of 2 g/kg body weight. Blood glucose levels were measured at 15, 30, 60, 90, and 120 min post-glucose challenge. The area under the curve (AUC) was calculated, and a glucose-time curve was plotted to assess changes in glucose tolerance.

#### ELISA assay

The levels of LH, FSH, T, E2, P, TNF-α, IL-6, IL-18, IL-1β, and FINS in the serum samples from each group of rats were determined according to the ELISA kit instructions. First, serum samples were allowed to equilibrate at room temperature for 30 min. Subsequently, the samples were diluted using the provided sample diluent at ratios ranging from 1:5 to 1:10. Standards and test samples were sequentially added to pre-coated plate wells, then incubated at 37°C for 60 min. After the plate was washed three times, the substrate solution (TMB) was added and incubated at 37°C for 30 min. When a distinct gradient appears in the standard wells (a clear blue gradient in the first four colorimetric wells), the stop solution was added to terminate the reaction. The optical density (OD) at 450 nm for each well was immediately measured using an enzyme-linked immunosorbent assay (ELISA) reader. The concentration of each indicator was then calculated based on the standard curve.

#### RT-qPCR analysis

The samples were reverse transcribed into cDNA templates, which were then incorporated into qPCR reaction systems. Following denaturation, annealing, and extension PCR steps, target genes were amplified with real-time detection of fluorescence signals. After amplification, melting curves were plotted, and the relative expression levels of *Occludin*, *Claudin-1*, and *Zo-1* in rat colon tissue, as well as *TNF-α*, *IL-6*, *IL-18*, *IL-1β*, *TLR4*, *P13K*, *AKt*, and *NF-κB p65* mRNA in ovarian tissue, were determined.

#### Western blot analysis

Ovarian tissue was lysed using RIPA buffer to extract total protein, and the protein concentration of the lysate was determined by the BCA protein assay. Following electrophoresis, membrane transfer, primary antibody incubation, secondary antibody incubation, ECL chemiluminescent substrate addition, and development results were analyzed using ImageJ software to assess the expression levels of TLR4, P13K, p-P13K, AKt, p-AKt, p65, and p-P65 proteins in rat ovarian tissue.

#### Metagenomic analysis

A standardized bioinformatics workflow was employed to analyze metagenomic sequencing data (27, 28). First, raw sequencing data underwent quality control processing using fastp software (version 0.23.2) to remove low-quality reads (default parameter settings applied). Subsequently, to exclude host DNA contamination, the quality-controlled sequencing data were aligned against the rat reference genome (mRatBN7.2) using Bowtie2 (version 2.4.5), and matched host sequences were removed. Finally, microbial community taxonomic analysis was performed using the MetaPhlAn4 platform (version 4.0.6) with its default marker gene database (mpa_vjan21_CHOCOPhlAnSGB).

#### Statistical analysis

Statistical analysis was performed using GraphPad Prism (v9.5.1). Data are expressed as mean ± standard error of the mean (SEM). Differences were considered statistically significant at *P <* 0.05, with significance levels denoted as follows: *P < 0.05, **P < 0.01, ***P < 0.001, ****P < 0.0001*.* Two sets of normally distributed data were analyzed using two-tailed Student’s *t*-tests; nonparametric data were analyzed using the Mann-Whitney *U* test. Multiple sets of normally distributed data were analyzed using one-way analysis of variance (ANOVA), followed by Tukey’s test or Dunnett’s test for multiple comparisons. Nonparametric data were analyzed using the Kruskal-Wallis test, followed by Dunn’s multiple comparison test. The anosim function from the vegan package was used to calculate the significance (*P-*value) and effect size (*R-*value) of community differences. Principal coordinate analysis (PCoA) was performed using the stats package (v3.5.0), and visualization was conducted using the ggplot2 package (v3.2.0). Linear regression analysis was performed using Origin 2024. Linear discriminant analysis (LDA), heatmap generation, mediation effect testing, and visualization of bacterial species interactions were conducted on an online platform.

## RESULTS

### This study reveals that *Scutellaria baicalensis* exerts significant therapeutic potential for PCOS through a multi-target mechanism, acting synergistically on key molecular targets

**Fig 2:**
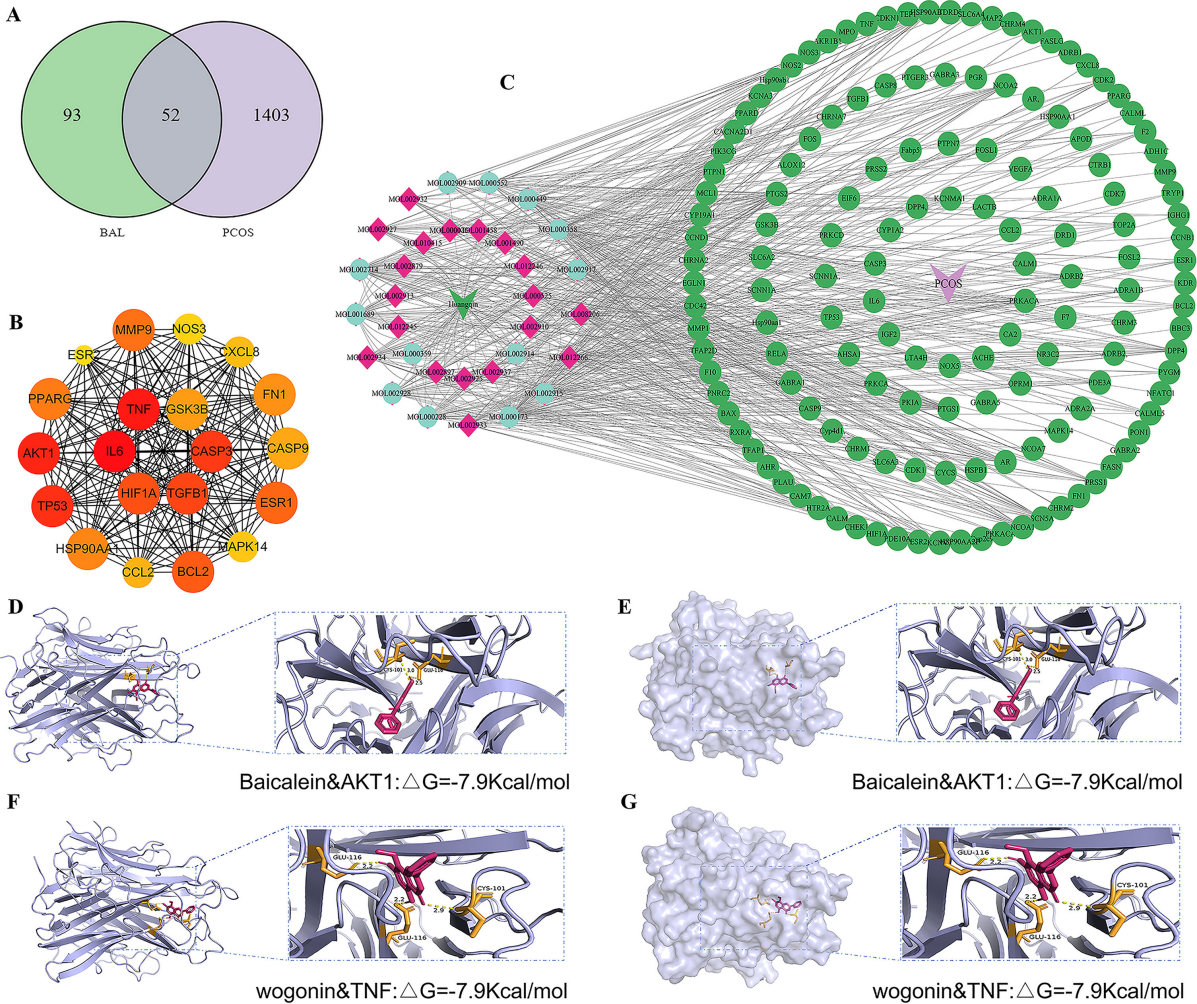
Network pharmacological analysis of *Scutellaria baicalensis* in PCOS management and molecular docking of *BAL*’s key active components with core targets. (**A**) The Venn diagram illustrates target interactions between *BAL* and PCOS. The green and purple sets represent the therapeutic targets of *BAL* and PCOS-associated targets, respectively. Their intersection identifies potential molecular targets for *BAL* in PCOS intervention. (**B**) *BAL* modulates the protein interaction network in PCOS. Nodes represent potential target proteins, with node size and color intensity increasing with degree values; edge thickness reflects the strength of protein interactions. (**C**) Molecular interaction network integration diagram. This network integrates the complex relationships among *BAL*’s active components, target molecules, and PCOS. *IL-6, TNF, AKT1, and CASP3* serve as core protein nodes, showing high overlap with the core targets identified in panel B. This reflects *BAL*’s multi-component, multi-target action profile. (**D–G**) Molecular docking validation. Three-dimensional diagrams of the binding patterns between *baicalein* and *AKT1*, and *wogonin* and *TNF*, along with key interaction sites. Free energy calculations indicate that both *baicalein* (−7.9 kcal/mol) and *wogonin* (−7.9 kcal/mol) exhibit strong binding affinity with *AKT1* and *TNF*, respectively.

Through screening from TCMSP, HERB databases, and relevant literature (29–31), a total of 36 bioactive compounds meeting the criteria for OB and DL were identified. After excluding compounds with unpredicted target information, 145 potential targets were obtained after deduplication. Subsequently, disease-related targets associated with PCOS were screened using databases such as GeneCards and DisGeNET. After consolidation and deduplication, 1,455 PCOS-related targets were obtained. An intersection analysis between *BAL* targets and PCOS targets yielded 52 potential overlapping targets, which may be involved in the mechanism of *BAL* treatment for PCOS (Fig. 2A). We further constructed an association table linking *BAL* active components and targets, then imported the data into Cytoscape 3.10.3 for analysis to evaluate the degree values of *BAL* active components (Table S1) and select the top 10 active components in *BAL* ranked by degree value. The PPI network derived from the STRING database was successfully constructed and visualized using Cytoscape 3.10.3. (Fig. 2B; Fig. S1). By analyzing the network using CytoHubba and CytoNCA modules, the top 20 targets ranked by degree value were identified and visualized. In the diagram, target colors and node sizes are directly proportional to their degree values—darker hues and larger areas indicate higher degree values for the corresponding target proteins. Simultaneously, the thickness of inter-node connections reflects interaction strength, with thicker lines signifying stronger interactions. Top-ranked targets include *TNF*, *IL-6*, *AKT1*, *TP53*, and *CASP3*. The *Scutellaria baicalensis*-component-target-PCOS network diagram (Fig. 2C) reveals that targets such as *IL-6*, *TNF*, *AKT1*, and *CASP3* are key components within this network. These targets largely overlap with the top 20 core targets identified in Fig. 2B. This network reveals that *BAL* exerts its therapeutic effects in treating PCOS through a multi-component and multi-target mechanism. Using an online tool (http://cloudtutu.com.cn/), GO and KEGG enrichment analyses were performed on the top 20 potential targets ranked by degree value in *BAL.* The results revealed enrichment in 11,527 biological processes (BP), 930 cellular components (CC), and 884 molecular functions (MF). In the secondary classification, the number of annotated genes for each category is displayed via bar charts (Fig. S2). Among these, BP primarily involves metabolic processes and immune system processes; CC mainly includes cellular anatomical entities and protein-containing complexes; whereas MF predominantly concerns molecular function regulator activity and catalytic catalysis. Furthermore, the analysis enriched 379 signaling pathways, with 27 major pathways visualized (Fig. S3). The length of each bar represents the number of annotated genes assigned to the pathway, which encompass systems such as the immune system, signal transduction, and cancer. Collectively, the effect of *BAL* intervention on PCOS results from the combined action of multiple mechanisms.

Among the active components in *BAL*, *baicalein*, *wogonin*, and *orocylin A* exhibit multiple target interactions. We selected these three components for molecular docking with *AKT1*, *IL-6*, and *TNF*, where the active components served as ligands and *AKT1*, *IL-6*, and *TNF* served as receptors. The results indicate that the evaluation parameter for docking outcomes is ΔG (expressed in kcal/mol), representing the free binding energy between the receptor protein and the ligand molecule. A ΔG value below −7.0 kcal/mol indicates that the receptor and ligand can naturally bind to each other under physiological conditions. Specifically, the binding energy between *baicalein* and *AKT1* is −7.9 kcal/mol (Fig. 2D and E), whereas that between *wogonin* and *AKT1* is −7.0 kcal/mol (Fig. S4). The binding energy between *wogonin* and *IL-6* was −6.4 kcal/mol (Fig. S5), and *orocylin A* exhibited a binding energy of −6.3 kcal/mol with *IL-6* (Fig. S6), whereas *wogonin* showed a binding energy of −7.9 kcal/mol with *TNF* (Fig. 2F and G). All combinations demonstrate strong binding affinity.

### Experimental studies confirm that *Scutellaria baicalensis* effectively suppresses body weight gain and restores normal estrous cyclicity in PCOS rat models

**Fig 3:**
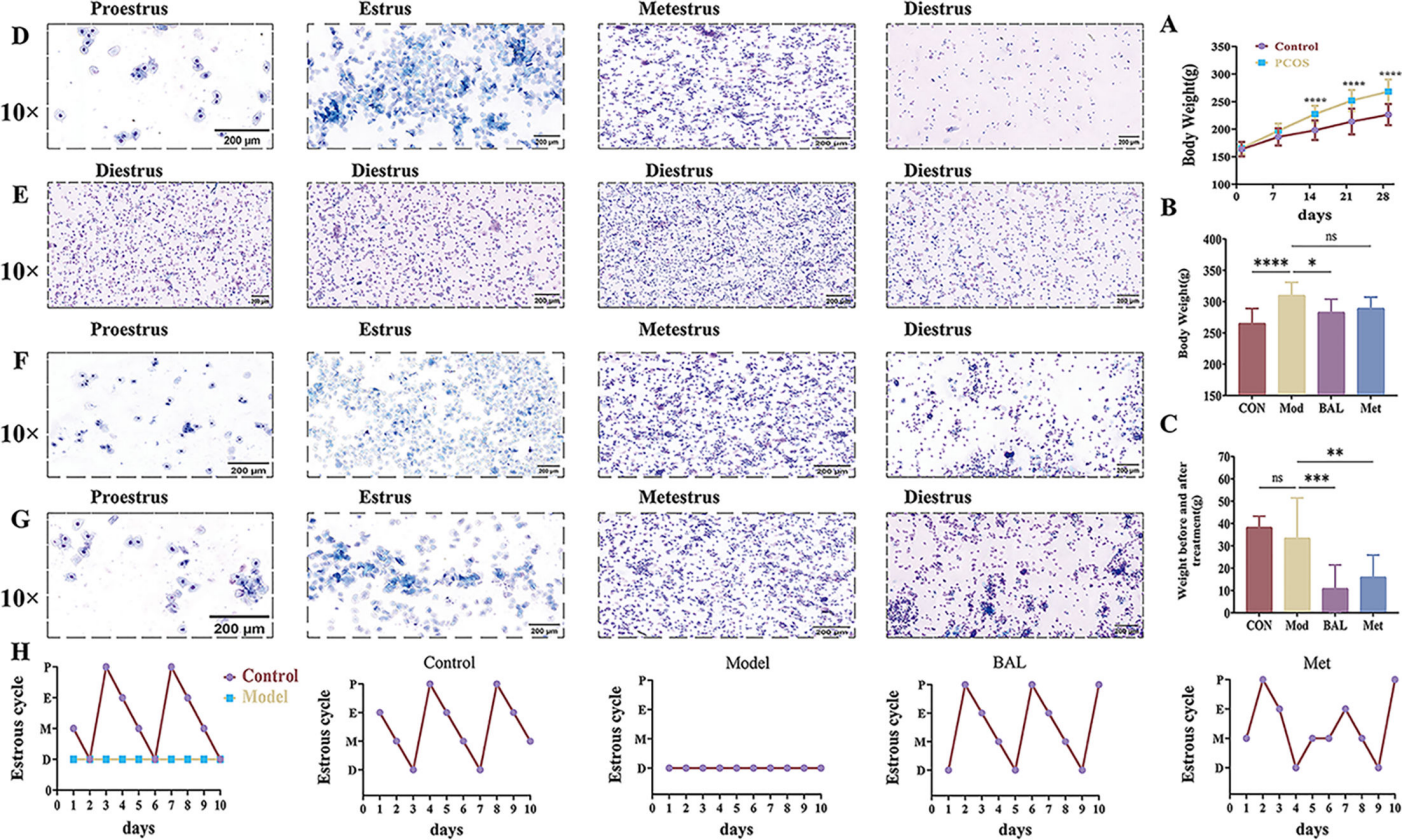
The therapeutic effects of *Scutellaria baicalensis* supplementation on metabolic and reproductive parameters in PCOS model rats. (**A**) Comparative body mass progression between healthy controls and PCOS-induced groups during disease induction. (**B**) Impact of *BAL* and Met interventions on weight regulation in PCOS model subjects. (**C**) Pre- and post-treatment body mass variations across experimental cohorts. (**D**) Vaginal cytology specimens (10 × magnification) illustrating characteristic estrus phase cellular patterns in control group rats: Proestrus—predominantly nucleated epithelial cells; Estrus—abundant keratinized squamous cells; Metestrus—tripartite mixture of nucleated/keratinized epithelial cells and leukocytes; Diestrus—leukocyte-dominated smears. (**E**) Quantitative representation of cyclicity restoration across treatment groups. (**F**) BAL group restored a regular and complete estrous cycle. (**G**) Met group although a relatively complete estrous cycle was observed, changes during each phase and cycle length remained irregular. (**H**) Line charts of the estrous cycles for each group.

The effect of *BAL* on body weight in PCOS rats showed that rats in the model group exhibited significantly increased body weight compared to the control group (*P* < 0.0001). Following intervention treatment, rats in the *BAL* group demonstrated a marked decrease in body weight compared to the Mod group (*P* < 0.05), while rats in the Met group showed a reduction in body weight that did not reach statistical significance (*P >* 0.05). Furthermore, no significant weight difference was observed between Mod and Con groups before and after treatment (*P* > 0.05). Following intervention, *BAL* rats exhibited a significant decrease in body weight both before and after treatment (*P* < 0.001), while Met rats also showed a significant weight reduction (*P* < 0.01). These findings indicate that *BAL* effectively mitigates weight gain in PCOS rats (Fig. 3A through C).

The estrous cycle of sexually mature female rats can be divided into four phases based on the characteristic changes in vaginal epithelial cells shed during each stage:

**(i) Proestrus**: Smears primarily contain nucleated epithelial cells, which are large and mostly oval-shaped, with occasional scattered white blood cells and keratinized epithelial cells.
**(ii) Estrus**: Smears reveal abundant keratinized epithelial cells exhibiting irregular shapes. These cells typically aggregate in large numbers, forming leaf-like clusters with nearly absent nuclei.
**(iii) Metestrus**: Smears contain nucleated epithelial cells, keratinized epithelial cells, and leukocytes in relatively balanced proportions.
**(iv) Diestrus**: Smears predominantly show small, deeply stained leukocytes with nuclei occupying nearly the entire cell. Nucleated epithelial cells and keratinized epithelial cells are occasionally visible.

Observations revealed that rats in the control group exhibited regular and complete estrous cycles, whereas model group rats showed no cyclic changes in the estrous cycle and remained persistently in the estrus interval. Following intervention treatment, the *BAL* group restored a complete estrous cycle. Although the Met group exhibited a relatively complete estrous cycle, the transitions between phases and cycle duration remained irregular (Fig. 3D and E).

### Experiments demonstrate that *Scutellaria baicalensis* can effectively correct insulin resistance, ovarian pathological damage, and endocrine disorders in PCOS rats

**Fig 4:**
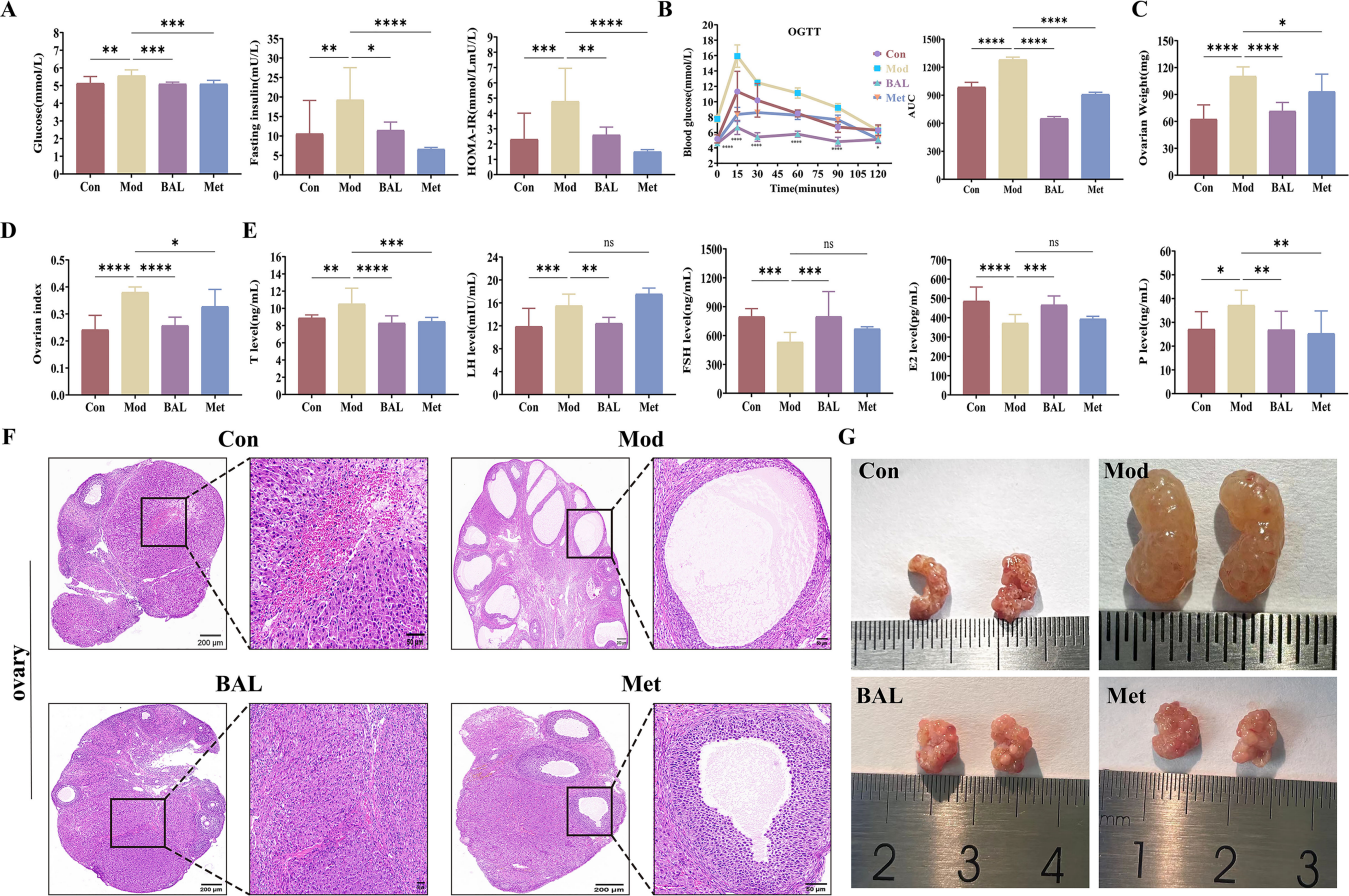
*Scutellaria baicalensis* improves insulin resistance, impaired glucose tolerance, and ovarian pathological damage in PCOS rats. (**A**) Fasting blood glucose, insulin, and HOMA-IR levels were assessed across groups. (**B**) OGTT results and AUC values are presented. (**C and D**) Quantitative analysis of ovarian mass and ovarian index. (**E**) Serum sex hormone concentrations were measured, revealing regulatory effects of *BAL* and Met. (**F**) H&E staining revealed improved ovarian follicular development and stromal structure following *BAL* and Met intervention. (**G**) Representative macroscopic images of bilateral ovaries from rats in each group are displayed.

Compared with the control group, rats in the model group exhibited significantly elevated fasting blood glucose (FBG) and fasting insulin (FINS) levels (*P <* 0.01), while the HOMA-IR index markedly increased (*P <* 0.001). Following intervention treatment, compared with Mod rats, *BAL* rats exhibited significantly decreased FBG (*P <* 0.001), markedly reduced FINS (*P <* 0.05), and significantly lowered HOMA-IR (*P <* 0.01). The Met group exhibited a significant decrease in FBG (*P <* 0.001), a significant reduction in FINS (*P <* 0.0001), and a significant decrease in HOMA-IR (*P <* 0.0001). These results indicate that *BAL* effectively improves insulin resistance in PCOS rats (Fig. 4A).

Following oral administration of 50% glucose solution, rats in the model group exhibited significantly elevated blood glucose levels at all time points compared to the control group, with markedly increased area under the curve (AUC) for blood glucose levels (*P* < 0.0001). Following intervention treatment, compared with Mod rats, *BAL* rats exhibited markedly reduced blood glucose levels at all-time points during the oral glucose tolerance test (OGTT), with a significant decrease in AUC (*P* < 0.0001). Similarly, Met rats also demonstrated significantly lower blood glucose levels at all OGTT time points, accompanied by a significant reduction in AUC (*P <* 0.0001). These results indicate that Mod rats exhibit diabetic impairment, while *BAL* effectively ameliorates diabetic issues in PCOS rats (Fig. 4B).

Effects of *BAL* on ovarian morphology in PCOS rats

Compared with the control group, the model group exhibited significantly increased ovarian weight and ovarian index (*P <* 0.0001). Following intervention treatment, ovarian weight and ovarian index in the *BAL* group were markedly reduced compared with the Mod group (*P* < 0.0001), while ovarian weight and ovarian index in the Met group also decreased significantly (*P* < 0.05) (Fig. 4C and D).

H&E staining revealed that ovaries in the Con group exhibited follicles at various developmental stages alongside a small number of corpus luteum. Granulosa cells (GCs) were neatly arranged with intact morphology, and no cystic follicle formation was observed. In contrast, ovarian tissue in the Mod group exhibited disorganized structure with numerous cystic follicles. The GCs layer was thinner and more loosely arranged, and oocytes were absent. Compared to the Mod group, the *BAL* group showed follicles at all stages, a thickened granulosa layer, and multiple distinct corpus luteum. No cystically dilated follicles were observed. The Met group showed a marked reduction in cystic follicles, with the presence of primary, secondary, and mature follicles alongside corpus luteum. The dominant follicles exhibited a thick, dense GCs layer, resembling the local morphology observed in the Con group. These findings indicate that *BAL* improves ovarian histopathological changes in PCOS rats (Fig. 4F and G).

Compared with rats in the control group, serum follicle-stimulating hormone (FSH) and estradiol (E2) levels were significantly decreased in rats in the model group (*P* < 0.001), while luteinizing hormone (LH), testosterone (T), and progesterone (*P*) levels were significantly elevated (*P* < 0.05, *P* < 0.01, *or P* < 0.001). Following intervention therapy, compared with the Mod group, serum FSH and E2 levels in the *BAL* group rats significantly increased (*P* < 0.001), while LH, T, and P levels significantly decreased (*P* < 0.01 or *P* < 0.0001). Serum FSH, E2, and LH levels in the Met group rats increased, but without statistical significance (*P >* 0.05), while T and P levels decreased significantly (*P <* 0.01 or *P <* 0.001). These results indicate that *BAL* effectively improves serum hormone disorders in PCOS rats (Fig. 4E).

### *Scutellaria baicalensis* alleviates systemic inflammation and mitigates ovarian/colonic tissue damage in PCOS rats by restoring intestinal barrier integrity and suppressing the *TLR4/NF-κB* pathway

**Fig 5:**
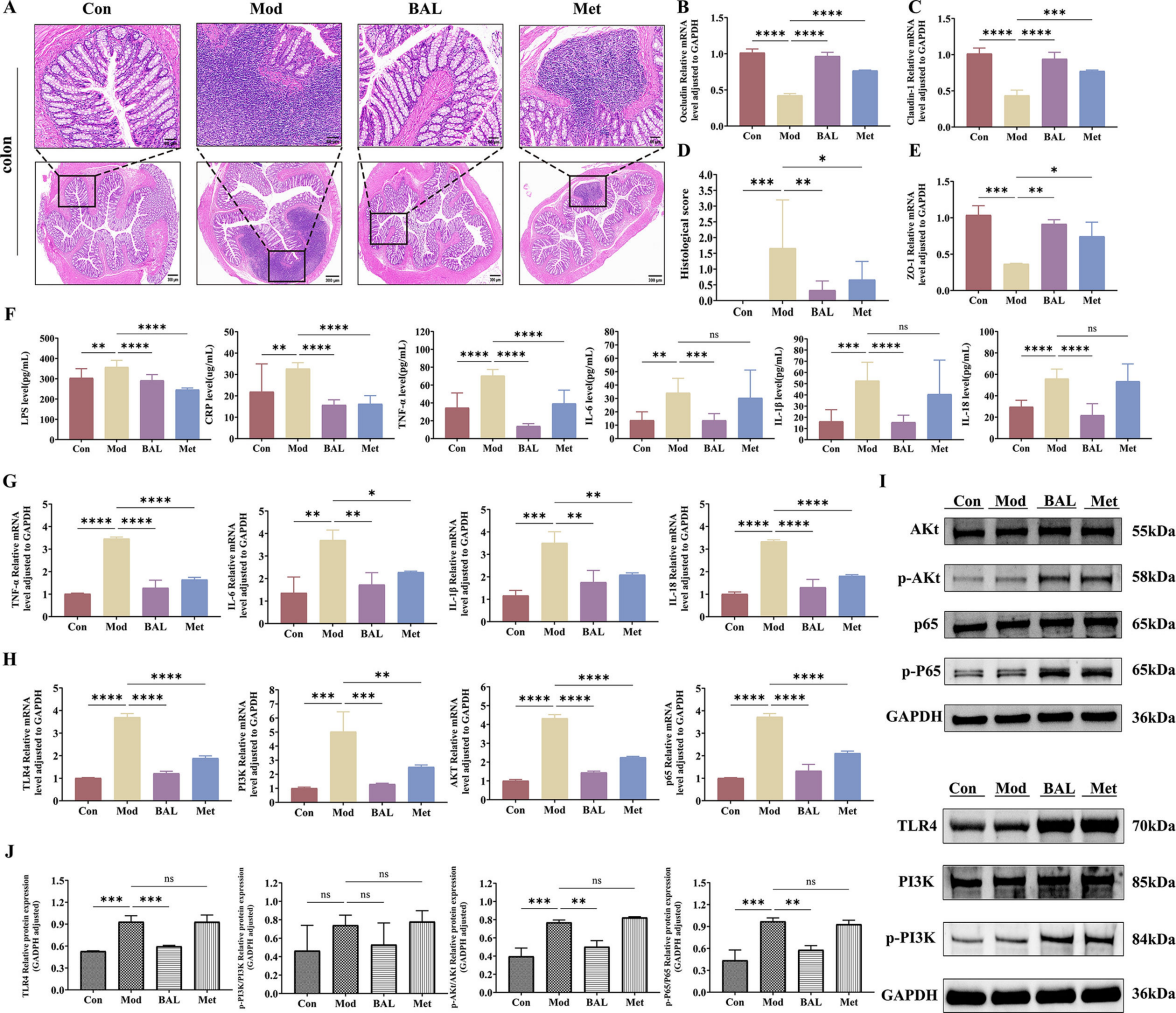
*Scutellaria baicalensis* improves intestinal barrier function and ovarian inflammation in PCOS rats by downregulating the *TLR4/NF-κB* pathway. (**A**) Representative hematoxylin-eosin (H&E) stained colonic tissue sections display histological features across experimental groups, with overview images captured at 5× magnification (200 μm scale bar) and detailed views at 20× magnification (60 μm scale bar). (**B**) Quantitative assessment of colonic tissue pathology through histological scoring. (**C–E**) Evaluation of colonic tight junction protein expression profiles including *Occludin*, *Claudin-1*, *and ZO-1*. (**F**) Comparative analysis of circulating inflammatory cytokine concentrations across experimental groups. (**G**) Measurement of pro-inflammatory cytokine mRNA levels (*TNF-α*, *IL-6*, *IL-1β*, *IL-18*) in ovarian tissues. (**H**) Transcriptional regulation analysis of *TLR4/PI3K/AKT* pathway components in each group of rats. (**I, J**) The expression patterns of *NF-κB p65* in ovarian tissues were systematically examined across different experimental groups. Quantitative analysis of TLR4, PI3K, AKT, and p65 protein levels in ovarian tissues was performed through standardized assessment protocols.

H&E staining of the colon revealed that in the control group rats, the colonic tissue layers were clearly defined, epithelial cells were neatly arranged, glandular structures were distinct and regularly organized, crypt structures were normal, and capillaries with scattered lymphocytes were visible within the lamina propria. In contrast, the model group exhibited markedly impaired colonic mucosal epithelial structure, with loosened epithelial cell junctions, deformed and reduced glands, disrupted and irregularly arranged crypts, and extensive inflammatory cell infiltration in the mucosal lamina propria. Histopathological scores were significantly higher than those in the control group (*P* < 0.001). Following intervention therapy, compared with the model group, the colonic pathological damage in the *BAL* group was significantly improved, with a marked decrease in histopathological scores (*P* < 0.01). Concurrently, the Met group also exhibited improved colonic pathological damage with markedly reduced histopathological scores (*P* < 0.05, Fig. 5A and B; Table S2).

The expression of intestinal tight junction proteins is often used as a key indicator for assessing intestinal barrier integrity. Damage to the intestinal barrier leads to a significant reduction in the expression of three important tight junction proteins: *Occludin*, *Claudin-1*, and *ZO-1*. As shown in Fig. 5C through E, compared with the control group, the mRNA expression levels of these three colonic tight junction proteins were significantly reduced in the model group rats (*P <* 0.001 or *P <* 0.0001). Following intervention treatment, mRNA expression levels of these three tight junction proteins were significantly elevated in the *BAL* group compared to the model group (*P <* 0.01 or *P <* 0.0001), while the Met group also exhibited significantly increased mRNA expression levels (*P <* 0.05, *P <* 0.001, or *P <* 0.0001). These findings indicate that *BAL* effectively improves impaired intestinal barrier function in PCOS rats.

The effects of *BAL* on serum inflammatory factors in PCOS rats were evaluated. Results showed that compared with the control group, serum levels of lipopolysaccharide (LPS), C-reactive protein (CRP), tumor necrosis factor (TNF-α), interleukin-6 (IL-6), interleukin-1β (IL-1β), and interleukin-18 (IL-18) levels were significantly elevated (*P* < 0.01, *P* < 0.001, or *P* < 0.0001). Following intervention treatment, compared with the model group, the levels of LPS, CRP, TNF-α, IL-6, IL-1β, and IL-18 in the *BAL* group were significantly reduced (*P* < 0.001 or *P <* 0.0001). Additionally, rats in the Met group exhibited significantly reduced levels of LPS, CRP, and TNF-α (*P* < 0.0001). Although IL-6, IL-1β, and IL-18 levels also decreased, these reductions did not reach statistical significance (*P* > 0.05). These findings indicate that *BAL* effectively ameliorates the chronic low-grade inflammatory state in PCOS rats (Fig. 5F).

We examined the effects of *BAL* on spleen weight and spleen index in PCOS rats. Results showed that the spleen index in the model group was significantly lower than that in the control group (*ANOVA, P* < 0.05), indicating immunosuppression under PCOS conditions. Although spleen weight decreased, no statistically significant difference was observed. Following *BAL* intervention, both spleen weight (*ANOVA, P* < 0.05) and spleen index (*ANOVA, P* < 0.01) significantly recovered compared to the Mod group, whereas the Met group showed no notable changes. These findings indicate that *BAL* effectively ameliorates immune abnormalities in PCOS rats (Fig. S8). We evaluated the effects of *BAL* on the mRNA expression levels of pro-inflammatory factors TNF-α, IL-6, IL-1β, and IL-18 in ovarian tissues of PCOS rats. Compared with the control group, the gene expression levels of pro-inflammatory factors in the ovarian tissue of the model group rats were increased to varying degrees (*P <* 0.01, *P* < 0.001, or *P* < 0.0001). Following intervention treatment, the mRNA expression levels of TNF-α, IL-6, IL-1β, and IL-18 were significantly reduced in the *BAL* rats (*P* < 0.01 or *P* < 0.0001). Additionally, the Met group exhibited reduced mRNA expression of TNF-α, IL-6, IL-1β, and IL-18 (*P* < 0.05, *P* < 0.001, or *P* < 0.0001), although these reductions did not reach uniform statistical significance. These findings indicate that *BAL* root extract effectively ameliorates chronic low-grade inflammation in ovarian tissues of PCOS rats (Fig. 5G).

The effects of *BAL* on the expression levels of *TLR4*, *PI3K*, *AKT*, and *NF-κB p65* mRNA in ovarian tissues of PCOS rats were evaluated. Compared with the control group, the expression levels of *TLR4*, *PI3K*, *AKT*, and *NF-κB p65* mRNA in ovarian tissues of the model group rats were significantly upregulated (*P <* 0.001 or *P <* 0.0001). Following intervention treatment, compared with the model group, the *BAL* group exhibited significantly reduced expression levels of *TLR4*, *PI3K*, *AKT*, and *NF-κB p65* mRNA (*P* < 0.001 or *P <* 0.0001). Additionally, the Met group exhibited varying degrees of decreased *TLR4*, *PI3K*, *AKT,* and *NF-κB p65* mRNA expression *(P <* 0.01 or *P <* 0.0001). These findings indicate that *BAL* effectively suppresses mRNA expression of the *TLR4/NF-κB* signaling pathway in PCOS rats (Fig. 5H).

We evaluated the effects of *BAL* on the expression levels of TLR4, PI3K, AKT, and p65 proteins in ovarian tissues of PCOS rats. Based on network pharmacology predictions, the key targets *TNF*, *IL-6*, *AKT1*, and *CASP3* were enriched in the immune system signaling pathway. Therefore, this study selected TLR4, p-PI3K/PI3K, p-AKT/AKT, and p-P65 protein expression in this pathway for detection to validate the network pharmacology predictions. Compared with the control group, the expression levels of TLR4, p-PI3K/PI3K, p-AKT/AKT, and p-P65/P65 in ovarian tissues of the model group rats were reduced to varying degrees (*P* > 0.05, *P* < 0.01, or *P* < 0.001). However, the Met group showed no significant decrease in the expression levels of TLR4, p-PI3K/PI3K, p-AKT/AKT, and p-P65/P65 *(P* > 0.05). These results indicate that *BAL* effectively inhibits the expression of proteins in the *TLR4/NF-κB* signaling pathway in PCOS rats (Fig. 5I and J).

### *Scutellaria baicalensis* intervention restores the intestinal mucosal barrier by enriching *Phocaeicola*, thereby improving systemic inflammation and endocrine disorders in a PCOS model

**Fig 6:**
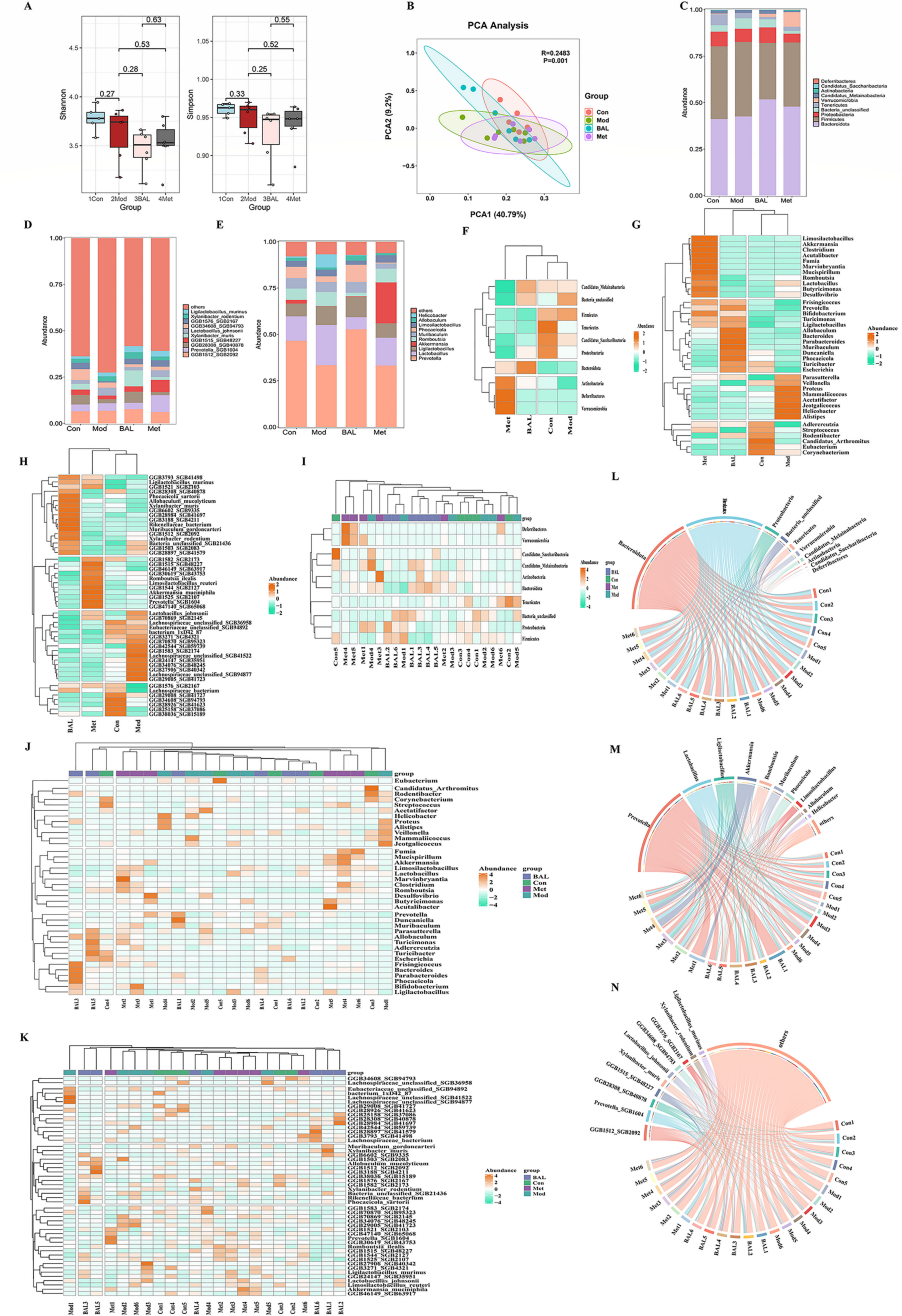
*Scutellaria baicalensis* improves gut dysbiosis in PCOS rats by enriching *Phocaeicola*. (**A**) Species *α* diversity; (**B**), species PCA. (**C–E**) Effects of *BAL* on PCOS gut microbiota composition (phylum, genus, species). (**F–K**) Bidirectional clustering heatmaps of community structure at phylum/genus/species levels (panel E shows mean-based clustering). (**L–N**) Chord diagrams of microbial proportion changes.

*BAL* significantly affected the *α* and *β* diversity of the gut microbiota in PCOS rats. Species diversity serves as a crucial metric for analyzing gut microbiota, with *α* diversity reflected by the *Shannon index* and *Simpson index* measuring species richness. Principal component analysis (PCA) in Fig. 6B revealed significant differences in gut microbiota species composition at the species level among groups (*P* = 0.001). Figure 6A shows that although there were no significant differences in *Shannon* and *Simpson* indices between groups, the *Shannon* and *Simpson* indices at the species level for gut microbiota composition in Mod rats were lower than those in Con rats. Following intervention treatment, both *BAL* and Met groups exhibited increased *Shannon* and *Simpson* indices, suggesting that *BAL* treatment partially enhanced the diversity and richness of gut microbiota in PCOS rats.

*BAL* improves gut microbiota dysbiosis in PCOS rats. Analysis of species abundance in the gut microbiota of rats across groups at the phylum, genus, and species levels (Fig. 6C, F, I, and L) revealed that the dominant phyla were *Bacteroidetes*, *Firmicutes*, *Proteobacteria,* and *Verrucomicrobia*, collectively accounting for over 88% of the total gut microbiota abundance in normal rats. Compared to the Con group, *Firmicutes* expression was elevated in the gut microbiota of Mod group rats. Following intervention therapy, *Firmicutes* expression in *BAL* and Met groups decreased significantly compared to the Mod group. Additionally, *Bacteroidetes* expression was lower in Mod rats than in *BAL* and Met rats, while the *Firmicutes*/*Bacteroidetes* (F/B) ratio was higher than in these two groups, indicating that *BAL* can improve gut dysbiosis in PCOS rats (Fig. S7). Figures 6D, G, J, and M show that at the genus level, the dominant microbiota include *Prevotella*, *Lactobacillus*, *Ligilactobacillus*, and *Akkermansia*, which collectively account for over 68% of the total abundance of the normal rat gut microbiota. Compared with the Con group, *Prevotella* expression in the gut microbiota of Mod group rats was significantly reduced. Following intervention, *Prevotella* expression increased in both *BAL* and Met groups relative to the Mod group. Additionally, *Ligilactobacillus* expression was higher in the *BAL* group than in the Mod group, while *Lactobacillus* and *Akkermansia* expression were both higher in the Met group than in the Mod group. These findings further indicate that *BAL* effectively improves gut microbiota dysbiosis in PCOS rats. Figures 6E, H, K, and N revealed that at the species level, the dominant microbiota included *Prevotella_SGB1604*, *Lactobacillus_johnsonii*, and *Ligilactobacillus_murinus*. Compared with the Con group, *Prevotella_SGB1604* expression was elevated in the gut microbiota of Mod group rats. Following intervention treatment, *Prevotella_SGB1604* expression in the *BAL* group was lower than that in the Mod group, while no significant decrease was observed in the Met group. Additionally, *Lactobacillus johnsonii* and *Ligilactobacillus murinus* expression in Mod rats was lower than in *BAL* and Met groups. Collectively, these findings indicate significant gut microbiota dysbiosis in PCOS rats, with *BAL* effectively improving this disordered state.

**Fig 7:**
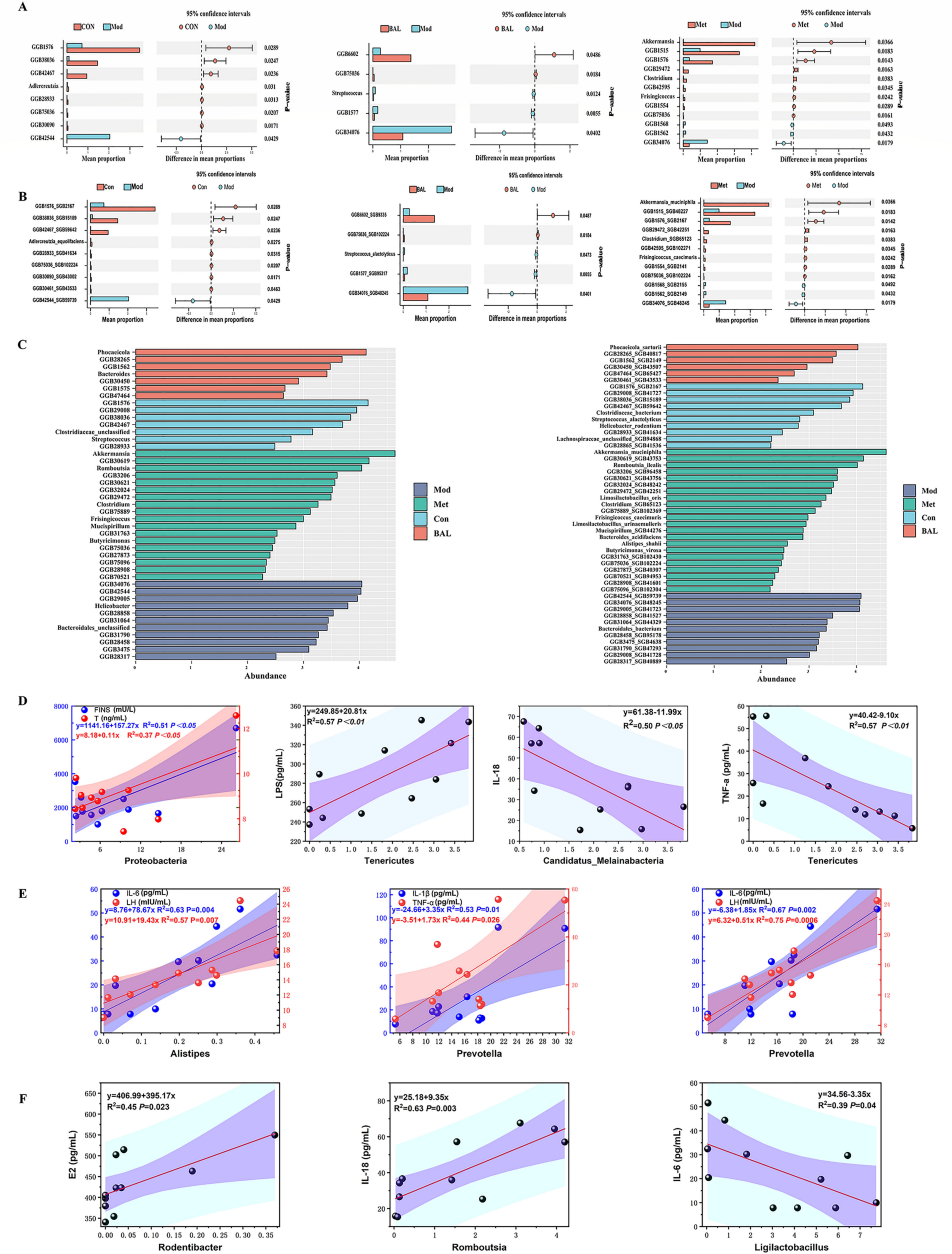
The genus *Phocaeicola* plays a pivotal role in *Scutellaria baicalensis’s* alleviation of PCOS, primarily by repairing the intestinal mucosal barrier to improve systemic inflammation and endocrine dysfunction. (**A, B**) STAMP analysis revealed the effects of *BAL* treatment on the distribution of gut microbiota at the genus and species levels in PCOS rats. (**C**) LEfSe analysis was employed to examine the abundance variation of gut microbiota at different taxonomic levels following *BAL* intervention. (**D, E, F**) The effects of *BAL* on PCOS gut microbiota were analyzed at the phylum and genus levels, followed by correlation studies linking changes in specific microbial communities to key biochemical parameters.

Fig. 7A and B show the stamp analysis of gut microbiota species abundance in PCOS rats treated with *BAL.* At the genus level, compared with the Con group, the abundance of *GGB42544* in the gut microbiota of Mod group rats significantly increased (*P* < 0.05), while the abundance of *GGB1576* and *GGB38036* significantly decreased (*P* < 0.05). Following intervention treatment, *Akkermansia* abundance significantly increased in the Met group (*P* < 0.05) compared to the Mod group. At the species level, the abundance of *GGB42544_SGB59739* in the gut microbiota of Mod rats was significantly higher than that in the Con group (*P* < 0.05), while the abundance of *GGB1576_SGB2167* was significantly lower than that in the Con group (*P* < 0.05). Following intervention treatment, the abundance of *GGB6602_SGB9335* in the gut microbiota of *BAL* rats was significantly increased compared to Mod rats (*P* < 0.05). Stamp analysis (Wilcoxon test, *P* < 0.05) suggests that *GGB42544* may serve as a potential marker microorganism in the gut microbiota of rats in the Mod group, *GGB6602_SGB9335* in the BAL group, and *Akkermansia* in the Met group.

Fig. 7C shows the LEfSe analysis of gut microbiota species abundance in PCOS rats treated with *BAL*. At the genus level, the abundance of *Bacteroidales_Unclassified* (*P* = 0.02, LDA = 3.42) and *Helicobacter* (*P* = 0.007, LDA = 3.79) was significantly higher in the Mod group than in other groups. In contrast, rats in the *BAL* group exhibited significantly higher abundance of *Phocaeicola* (*P* = 0.007, LDA = 4.12) and Bacteroides (*P* = 0.04, LDA = 3.42) compared to other groups. Rats in the Met group showed significantly increased abundance of *Akkermansia* (*P* = 0.002, LDA = 4.64), *Romboutsia* (*P* = 0.03, LDA = 4.04), and *Clostridium* (*P* = 0.009, LDA = 3.26). At the species level, *Bacteroidetes_bacterium* (*P* = 0.02, LDA = 3.36) was also more abundant in the gut microbiota of Mod group rats than in other groups. The *BAL* group exhibited significantly higher abundance of *Phocaeicola_sartorii* (*P* = 0.007, LDA = 4.03) compared to other groups. Additionally, the abundances of *Akkermansia_muciniphila* (*P* = 0.002, LDA = 4.62), *Romboutsia ilealis* (*P* = 0.03, LDA = 4.02), *Clostridium SGB65123* (*P* = 0.009, LDA = 3.25), and *Limosilactobacillus oris* (*P* = 0.02, LDA = 3.35) in the Met group were significantly higher than those in the other groups. LEfSe analysis (*P* < 0.05, *LDA* > 3) indicated that *Phocaeicola* is a potential key genus for *BAL* treatment of gut dysbiosis in PCOS rats, while *Bacteroides*, *Romboutsia*, *and Clostridium* were potential key genera for Met treatment of gut dysbiosis in PCOS rats. At the species level, *Phocaeicola sartorii* was identified as a potential key species for BAL treatment of gut microbiota dysbiosis in PCOS rats, while *Akkermansia muciniphila*, *Clostridium SGB65123*, and *Limosilactobacillus oris* were identified as key species for Met treatment of gut microbiota dysbiosis in PCOS rats.

Fig. 7D through F show linear regression analysis of *BAL* intervention on PCOS-related biological parameters in rats. At the phylum level (Fig. 7D), the gut bacterium *Proteobacteria* showed significant positive correlations with FINS and T, with *R*² = 0.51 (*P <* 0.05) for FINS and *R*² = 0.37 (*P* < 0.05) for T. Additionally, gut bacteria *Tenericutes* showed a significant positive correlation with LPS (*R*² = 0.57, *P* < 0.01). Conversely, *Candidates_Melainabacteria* showed a significant negative correlation with IL-18 (*R*² = 0.50, *P* < 0.05), while *Tenericutes* exhibited a significant negative correlation with TNF-α (*R*² = 0.57, *P < 0.01*). At the genus level (Fig. 7E), the gut bacterium *Alistipes* showed significant positive correlations with IL-6 and LH (IL-6: *R*² = 0.63, *P = 0.004*; LH: *R*² = 0.57, *P* = 0.007). Simultaneously, *Precotella* showed significant positive correlations with IL-1β, TNF-α, IL-6, and LH (IL-1β: *R*² = 0.53, *P =* 0.01; TNF-α: *R*² = 0.44, *P =* 0.026; IL-6: *R*² = 0.67, *P =* 0.002; LH: *R*² = 0.75, *P =* 0.0006). *Rodentibacter* also showed a significant correlation with E2 (*R*² = 0.45, *P =* 0.023). Furthermore, *Romboutsia* exhibited a significant positive correlation with IL-18 (*R*² = 0.63, *P =* 0.003). Notably, *Ligilactobacillus* exhibited a significant negative correlation with IL-6 (*R*² = 0.39, *P =* 0.04). Collectively, these findings indicate that these gut bacteria play a crucial role in the therapeutic effects of *BAL* on PCOS rats. The underlying mechanism may involve repair of the intestinal mucosal barrier, thereby promoting normalization of inflammatory states and hormone levels.

## DISCUSSION

Our research has demonstrated that *BAL* improves ovarian and colonic pathological conditions in PCOS rats while also restoring their disrupted estrous cycles, sex hormones, and inflammatory factors. Through metagenomic sequencing of fecal samples, we further confirmed *BAL*’s efficacy in ameliorating gut microbiota dysbiosis in PCOS rats.

We employed network pharmacology methods to screen 36 bioactive compounds from *BAL*, including *wogonin*, *beta-sitosterol*, *oroxylin A*, *acacetin*, *moslosooflavone*, and *baicalein.* These compounds act on 52 targets involved in regulating PCOS. Notably, *wogonin* exhibits multiple pharmacological activities, including anti-inflammatory, antiviral, antioxidant, and antitumor effects (32). Increasing evidence suggests that the pathogenesis of PCOS is associated with inflammation (33). *Beta-sitosterol* can reverse the imbalance of gut microbiota in the pathogenesis of PCOS and improve the progression of PCOS. Studies indicate that *beta-sitosterol* has the ability to regulate sex hormone balance in PCOS mice while demonstrating antioxidant stress capabilities. *Beta-sitosterol* may exert its protective effects by altering the composition and structure of gut microbiota in PCOS mice (34). *Oroxylin A* is a naturally occurring flavonoid with multiple pharmacological effects, including antioxidant, anti-inflammatory, and hepatoprotective properties. It modulates the *TNF-α* and nuclear factor *κB* (NF-κB) signaling pathways, thereby treating inflammatory diseases (35). *Baicalein* is a flavonoid compound renowned for its multifaceted pharmacological activities, particularly its anti-inflammatory properties. This anti-inflammatory effect stems from its ability to effectively modulate signaling pathways involved in the inflammatory response (18). Through PPI analysis, we identified key targets including *TNF*, *IL-6*, *AKT1*, *TP53*, and *CASP3*. Gene Ontology (GO) functional enrichment analysis suggests that *BAL* primarily exerts its molecular function regulator activity and catalytic activity by regulating biological processes such as metabolic processes and immune system processes, primarily through cellular components (CC) including cellular anatomical entities and protein-containing complexes. In the Kyoto Encyclopedia of Genes and Genomes (KEGG) enrichment analysis, we found that *BAL* treatment for PCOS primarily involves multiple signaling pathways, including the *NF-κB* signaling pathway, *PI3K-AKT* signaling pathway, Toll-like receptor (TLR) signaling pathway, and *TNF* signaling pathway, as well as disease-related pathways such as the *AGE-RAGE* signaling pathway in diabetic complications, human T-cell leukemia virus type 1 infection, Salmonella infection, insulin resistance, and fluid shear stress-mediated atherosclerosis. From the PPI network analysis, we identified the top 20 key targets ranked by degree value, with *TNF*, *IL-6*, and *AKT1* ranking prominently. We further conducted molecular docking analysis between *TNF, IL-6,* and *AKT1* with the core active components of *BAL*, including *wogonin*, *oroxylin A*, and *baicalein*. The results demonstrated low binding energies and high binding affinities between ligands and receptors. Based on comprehensive enrichment analysis, we identified core therapeutic targets for *BAL* in PCOS primarily enriched in *TNF*, *TLR4*, *NF-κB*, and *PI3K/AKT*-related pathways. In future studies, we will combine high-throughput technologies such as network pharmacology and proteomics to systematically identify the potential target profiles of the bioactive components in *Scutellaria baicalensis* and validate their functions through gene silencing and overexpression to elucidate the multi-target synergistic mechanism underlying its treatment of PCOS. Notably, dysbiosis of the gut microbiota compromises the intestinal mucosal barrier, allowing LPS produced by Gram-negative bacteria to enter the circulation. LPS binds to *TLR4* on immune cell surfaces, thereby activating the *NF-κB* signaling pathway. Therefore, the animal experiments in this study focused on the *LPS/TLR4/NF-κB* signaling pathway and were validated using real-time quantitative PCR and Western blot analysis.

The clinical manifestations of PCOS vary widely, and its etiology is highly complex. Therefore, establishing an animal model that closely mimics clinical features while minimizing invasiveness is crucial for in-depth research into this disease. Extensive research indicates that the letrozole-induced PCOS rat model exhibits marked weight gain, estrous cycle disruption, insulin resistance, hyperandrogenism, and polycystic ovarian tissue changes. These pathophysiological features largely mirror the alterations experienced by clinical PCOS patients, making this model widely adopted in animal studies of PCOS (36). Therefore, this study employed 5–6-week-old female SD rats to establish a PCOS model via oral administration of letrozole. Experimental results demonstrated that rats in the model group exhibited significant weight gain, estrous cycle disruption, insulin resistance, polycystic-like ovarian tissue changes, and dysregulation of sex hormone levels. These observations confirmed that the established model closely mirrors the physiological characteristics and pathological changes observed in PCOS patients. These findings indicate the successful establishment of a PCOS rat model, consistent with results from other relevant studies (37, 38). This further validates the efficacy of the methodology employed in this study and its potential application in PCOS research. The study demonstrates that *BAL* intervention significantly ameliorates reproductive and metabolic disorders in a rat model of PCOS. Following intervention, the animals‘ estrous cycles returned to normal levels, the number of granulosa cells in ovarian tissue increased significantly, and cystic dilatation within the follicular lumen markedly improved. These changes not only indicate restored ovarian function but also suggest *BAL*’s potential role in regulating ovarian physiological mechanisms. Furthermore, *BAL* effectively alleviated PCOS-related metabolic issues, such as weight gain, hormonal imbalance, and insulin resistance. These findings underscore the efficacy of *BAL* in treating the letrozole-induced PCOS rat model, further supporting its potential application in managing related reproductive endocrine disorders.

The *LPS/TLR4/NF-κB* signaling pathway is a classic pathway of the inflammatory response. When intestinal barrier function is compromised, LPS released by Gram-negative bacteria in the gut enter the bloodstream. Here, lipopolysaccharide-binding protein (LBP) binds to the Toll-like receptor 4 (TLR4) complex on the surface of innate immune cells, thereby activating the *NF-κB* signaling pathway. This process leads to the release of numerous pro-inflammatory factors, such as TNF-α, IL-6, IL-18, and IL-1β, thereby promoting chronic inflammation in PCOS. In the pathological context of PCOS, gut-derived LPS may induce a systemic inflammatory cascade by persistently activating the TLR4/NF-κB signaling pathway. This process has exacerbated lymphocyte apoptosis within splenic tissue while suppressing its regenerative capacity, ultimately leading to splenic atrophy and compromised immune function. Furthermore, elevated inflammatory factors not only contribute to insulin resistance but also increase free testosterone levels by suppressing hepatic synthesis of sex hormone-binding globulin or directly stimulating ovarian theca cells to produce androgens. This inhibits follicular growth and may even cause follicular atresia. Research indicates that repairing the intestinal barrier and inhibiting the *LPS/TLR4/NF-κB* signaling pathway can improve ovarian function and alleviate PCOS-related reproductive and metabolic disorders (39, 40). In our study, serum levels of pro-inflammatory factors such as C-reactive protein (CRP), LPS, TNF-α, IL-1β, IL-6, and IL-18 were significantly elevated in PCOS rats. mRNA expression of three tight junction proteins—*Claudin-1*, *Occludin*, and *ZO-1*—was markedly reduced in colonic tissue, with evident damage to the colonic mucosal epithelial structure. Additionally, in PCOS rats, spleen weight exhibited a decreasing trend without statistical significance (ANOVA, *P >* 0.05), whereas the spleen index was significantly reduced (ANOVA, *P <* 0.05), suggesting impaired systemic immune function. Moreover, mRNA expression of *TNF-α*, *IL-1β*, *IL-6,* and *IL-18* in ovarian tissue was markedly elevated, alongside significant increases in mRNA expression of *TLR4*, *PI3K*, *AKT,* and *NF-κB p65.* Notably, *BAL* intervention effectively reversed these alterations, reduced inflammatory factor levels in serum and ovarian tissue, improved colonic tight junction protein expression and mucosal structure, restored spleen index, and suppressed abnormal expression of the TLR4/PI3K/AKT/NF-κB p65 signaling axis in ovarian tissue, suggesting that *BAL* may alleviate immune dysregulation in PCOS rats by targeting the LPS/TLR4/NF-κB pathway, suppressing its excessive activation, thereby restoring immune homeostasis, modulating the local ovarian inflammatory microenvironment, and improving ovarian function. In summary, the *LPS/TLR4/NF-κB* signaling pathway was activated in PCOS rats, and *BAL* intervention successfully suppressed its activity, providing an effective intervention strategy for PCOS management.

**Fig 8:**
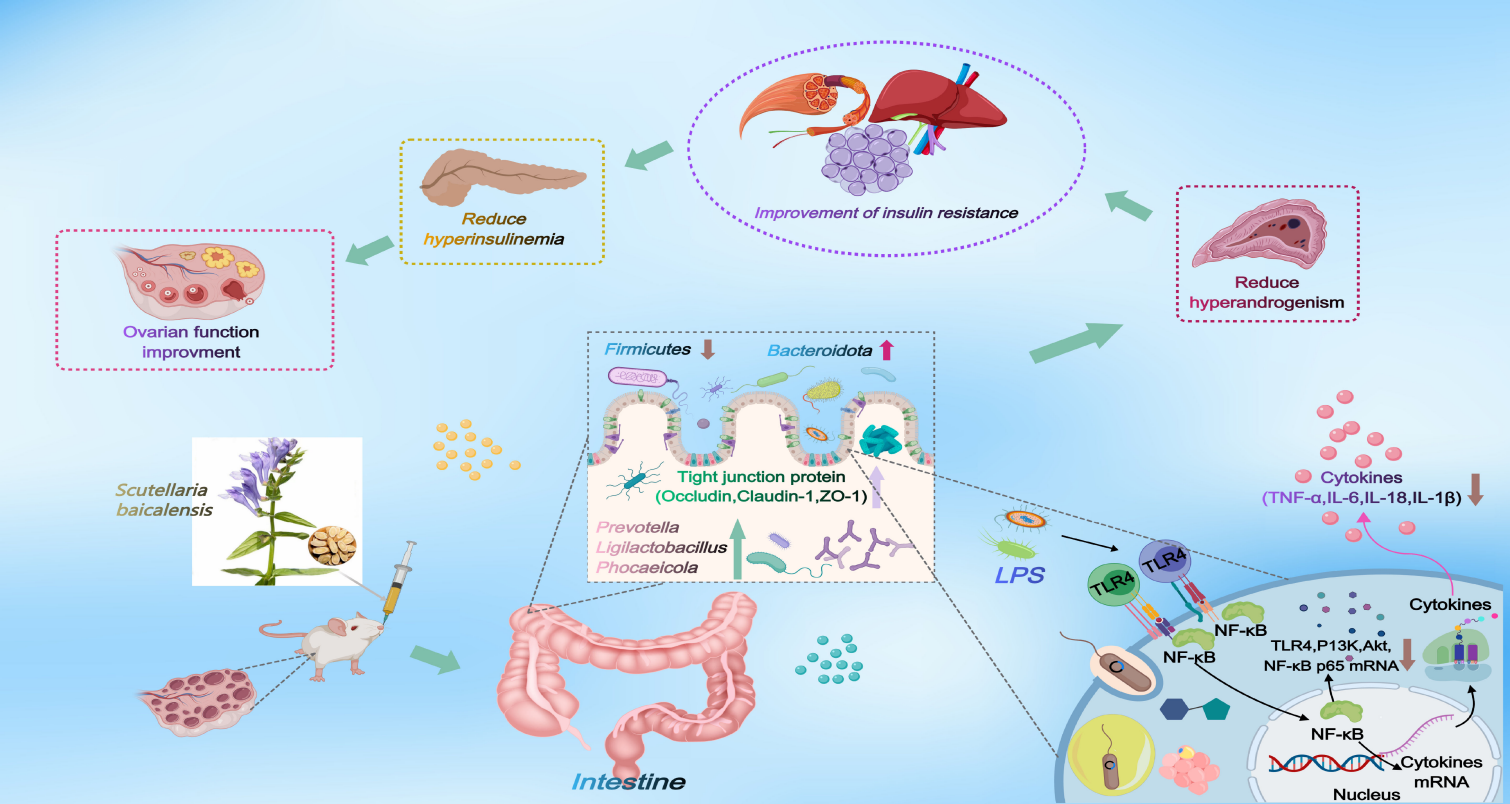
Schematic diagram of the proposed mechanism by which Scutellaria baicalensis ameliorates reproductive and metabolic dysregulation in PCOS rats via gut microbiota modulation.

A growing body of research evidence suggests that improving dysbiosis of the gut microbiota may be an important therapeutic strategy for PCOS (41). In a metagenomic sequencing study of the gut microbiota, we found that *BAL* significantly increased the *α* diversity index in PCOS rats, indicating enhanced species diversity and richness within the gut microbiota. Through *β* diversity analysis, results further revealed that *BAL* and Met interventions significantly altered gut microbiota composition. Analysis of gut microbial species abundance demonstrated that *BAL* effectively improved dysbiosis in PCOS rats. Additionally, Stamp and LEfSe analyses identified significant differences in gut microbial species abundance following *BAL* and Met interventions. At the genus level, *Phocaeicola* was identified as a potential marker microorganism for the *BAL* group, while *Akkermansia* and *Clostridium* were considered potential marker microorganisms for the Met group. At the species level, we further confirmed *Phocaeicola sartorii* as a characteristic microorganism of the *BAL* group, while *Akkermansia muciniphila* and *Clostridium SGB65123* were characteristic microorganisms of the Met group. Therefore, we speculate that the effect of *BAL* in improving gut dysbiosis in PCOS rats is closely related to the increased abundance of *Phocaeicola. Phocaeicola* is a Gram-negative genus widely present in the human gut, playing a crucial role in regulating host health. As a non-spore-forming, non-motile anaerobic rod, *Phocaeicola* is a common and important commensal microorganism in the human gut, possessing the ability to reduce inflammation and regulate gut microbiota and cytokine levels. Research indicates that gut microbiota dysbiosis is a key factor in the pathogenesis of PCOS, and increased *Phocaeicola* abundance may help restore gut microbial balance. By modulating lipopolysaccharide production and reducing systemic inflammation, it protects patients’ metabolic health (42, 43) (Fig. 8). Therefore, promoting the growth of *Phocaeicola* or introducing supplements containing this genus may become a potential therapeutic strategy for PCOS in the future. We also recognize that this study has certain limitations; please refer to the Supplemental materials for a detailed discussion.

### Impact analysis study

The beneficiary populations of this study include patients with PCOS accompanied by metabolic disturbances, immune dysregulation, and chronic low-grade inflammation, as well as researchers in related fields and developers of PCOS therapeutics. This study proposes that dysregulation of the “gut-immune-ovarian” axis serves as a driving mechanism of PCOS, and elucidates the value of *BAL* in restoring immune homeostasis and complementing metformin therapy. Future research should validate the mechanisms underlying this axis, identify the key active components of *BAL*, and verify the predictive value of *Phocaeicola* as a biomarker. For further details, please refer to the Supplemental materials.

### Conclusion

This study employed network pharmacology methods to analyze the active components, target molecules, and potential mechanisms of *BAL* in the treatment of PCOS, which were validated through molecular docking and animal experiments. Results indicate that *BAL* improves symptoms, signs, and reproductive metabolic disorders in PCOS rats via multi-component, multi-target pathways while reducing pro-inflammatory factor levels. Its mechanism likely involves regulating gut microbiota dysbiosis and alleviating intestinal inflammation, inhibiting activation of the *LPS/TLR4/NF-κB* signaling pathway, thereby participating in PCOS pathological progression and exerting therapeutic effects. This study provides scientific evidence for elucidating the modern mechanism of *BAL* in treating PCOS and supports future clinical research. Furthermore, this study underscores the crucial role of *BAL* in regulating immune and inflammatory responses. By thoroughly analyzing the interactions between active components and targets, along with the specific mechanisms influencing downstream signaling pathways, we provide a more comprehensive understanding of *BAL*’s application in PCOS treatment. This not only offers new perspectives for exploring the potential value of traditional Chinese herbs in modern medicine but also presents novel therapeutic approaches for PCOS disease management. However, the metabolic pathways of *BAL*’s active components and the synergistic or antagonistic interactions among these components remain unclear; this study did not explore these aspects in depth, which constitutes a major limitation (see the “Conclusions” section in the Supplemental materials).

## Supplementary Material

Reviewer comments

## Data Availability

The metagenomic sequencing data sets for fecal samples from our study have been deposited into the Sequence Read Archive (SRA) database at NCBI under BioProject accession number PRJNA1451408.

## SUPPLEMENTAL MATERIAL

Supplemental material msystems.01825-25-s0001.docx Supplemental text, figures, and tables.

Graphical abstract msystems.01825-25-s0002.tif Overview.

OPEN PEER REVIEW reviewer-comments.pdf An accounting of the reviewer comments and feedback.

ASM does not own the copyrights to Supplemental Material that may be linked to, or accessed through, an article. The authors have granted ASM a non-exclusive, world-wide license to publish the Supplemental Material files. Please contact the corresponding author directly for reuse.
