# Supplementary material for: Targeting the gut-ovarian axis: Scutellaria baicalensis improves polycystic ovary syndrome by modulating gut microbiota composition and inhibiting the LPS/TLR4/NF-κB signaling pathway
Source: mSystems. 2026 Jun 15;11(7):e01825-25. doi: 10.1128/msystems.01825-25 (PMC13386983; doi:10.1128/msystems.01825-25)
Supplement: Supplemental material — Supplemental text, figures, and tables. [file msystems.01825-25-s0001.docx]

**Supplementary Material**

**Targeting the Gut-Ovarian Axis: *Scutellaria baicalensis* Improves Polycystic Ovary Syndrome by Modulating Gut Microbiota Composition and Inhibiting the *LPS/TLR4/NF-κB* Signaling Pathway**

Zhengxiu Pan^1,2,#^, Caili Zhang^3,#^,Qiangqiang Chu^4^, Mingwei Chen^1^, Cancan Hui^5^, Tianjuan Wang^6,*^, Maozhen Han^3,*^,

Datong Deng^1,7,*^

^1^Department of Endocrinology and Metabolism, The First Affiliated Hospital of Anhui Medical University, Hefei, China.

^2^Endocrinology Department of He County Traditional Chinese Medicine Hospital, Ma'anshan, Anhui, China.

^3^College of Life Sciences, Anhui Medical University, Hefei, China.

^4^Anhui Tongling Bionic Technology Co., Ltd., Hefei, China.

^5^Department of Geriatric Endocrinology, The First Affiliated Hospital of Anhui Medical University, Hefei, China.

^6^Department of Reproductive Medicine, The First Affiliated Hospital of Anhui Medical University, Hefei, China.

^7^Institute of Endocrinology and Metabolism, Anhui Medical University, Hefei, China.

^#^These authors contributed equally to this work.

*Corresponding author E-mail: dengdayong@ahmu.edu.cn; hanmz@ahmu.edu.cn; [wangtianjuan@fy.ahmu.edu.cn](mailto:wangtianjuan@fy.ahmu.edu.cn)

**Design and Preliminary Results of the Preliminary Experiment**

To preliminarily evaluate the dose-response relationship and safety of *Scutellaria baicalensis* in improving PCOS, a small number of PCOS rats were randomly divided into a model group and *Scutellaria baicalensis* treatment groups receiving low (25 mg/kg), medium (50 mg/kg), and high (100 mg/kg) doses(1-3), Each group consisted of three rats, which received the drug via oral gavage for 21 consecutive days; a control group was also included. Efficacy was assessed based on the estrous cycle, serum testosterone levels, and ovarian morphology, while safety was evaluated using general clinical indicators.

Preliminary results indicated that all doses of *Scutellaria baicalensis* improved the estrous cycle and reduced serum testosterone levels in rats with PCOS. The 50 mg/kg group showed the best efficacy, but there was no significant difference compared to the 100 mg/kg group (Fig. S9), suggesting that the 50 mg/kg dose may have reached a plateau. No deaths, behavioral abnormalities, or weight loss were observed in any of the treatment groups (Fig. S10), indicating that *Scutellaria baicalensis* exhibited good safety within the 50–100 mg/kg dose range.

**2 Materials and Methods**

**2.7 Molecular Docking Validation**

In the PPI network diagram, the core target is the receptor, and the active compound is selected as the ligand for molecular docking.

**2.7.1 Strategies for screening core targets**

This study employed a “core-key-validation” stepwise approach to screen for targets: first, a “component-target” network was constructed using network pharmacology and ranked by degree; then, central hub nodes were identified through protein interaction network analysis. Ultimately, TNF, IL-6, and AKT1 were identified as core validation targets due to their high network connectivity and their roles as central regulatory factors in inflammatory and insulin signaling pathways. The literature confirmed that these targets are closely associated with chronic inflammation, insulin resistance, and ovarian dysfunction in PCOS(4, 5). Currently, most studies combining network pharmacology with molecular docking employ methods based on PPI networks to identify hub genes, which is consistent with the mainstream approach in this field(6-8).

**2.7.2 Screening of bioactive compounds and validation via molecular docking**

Three flavonoid compounds—*wogonin, baicalein*, and *oroxylin A*—which are abundant in *Scutellaria baicalensis* and exhibit significant biological activity, were selected as core ligand molecules. These compounds are recognized as the fundamental basis for the pharmacological effects of *Scutellaria baicalensis* (9) and possess well-defined anti-inflammatory and metabolic regulatory activities. Previous studies have confirmed that these components exert biological effects by regulating inflammatory factors such as TNF-α and IL-6, as well as the PI3K-AKT signaling pathway (10, 11), This is consistent with the functional characteristics of the three core targets identified in this study. Accordingly, this study used the three bioactive compounds as ligands to validate TNF, IL-6, and AKT1 through molecular docking, thereby evaluating the core “compound-target” interactions predicted by network pharmacology with minimal resource investment.

**Supplementary Tables and Figures**

**Table S1.** Active ingredients of *Scutellaria baicalensis* (Top 10).

**Fig.S1. PPI Network of**Scutellaria baicalensis**in Treating Polycystic Ovary Syndrome.**

**Fig.S2. GO Enrichment Analysis.**

**Fig.S3.** KEGG Enrichment Analysis.

**Fig.S4. Molecular Docking of *Baicalein* Active Constituent *Wogonin* with Key Protein *AKT1* and Corresponding Binding Free Energy (ΔG, kcal/mol).**

**Fig.S5.** Molecular Docking Analysis of *Baicalein's* Active Component *Wogonin* with *IL6* Protein and Associated Binding Energy (ΔG, kcal/mol).

**Fig.S6.**Molecular Interaction Study of *Baicalein* Derivative *Oroxylin A* with *IL6* Protein and Corresponding Binding Energy (ΔG, kcal/mol).

**Tab S2.** Pathological scoring criteria for rat colon tissue.

**Fig.S7** Relative abundance of *Firmicutes* and *Bacteroidetes* and their ratio(*F/B* ratio) in each group.

**Fig.S8** Quantitative analysis of spleen weight and spleen index.

**Fig.S9** Effects of low (25 mg/kg), medium (50 mg/kg), and high (100 mg/kg) doses of *Scutellaria baicalensis* on serum testosterone levels.

**Fig.S10** Comparison of body weight changes among different groups of rats on the 22nd day.

**Table S1 Active ingredients of *Scutellaria baicalensis*（Top 10）**

Mol ID Molecule Name OB (%) DL Degree Order Number

MOL000173 wogonin 30.68 0.23 45 BAL2

MOL000358 beta-sitosterol 36.91 0.75 38 BAL4

MOL002714 baicalein 33.52 0.21 37 BAL13

MOL000449 Stigmasterol 43.83 0.76 29 BAL6

MOL002928 oroxylin a 41.37 0.23 26 BAL27

MOL001689 acacetin 34.97 0.24 26 BAL12

MOL008206 Moslosooflavone 44.09 0.25 25 BAL32

MOL012266 rivularin 37.94 0.37 22 BAL35

MOL002934 NEOBAICALEIN 104.34 0.44 22 BAL30

MOL000552 5,2'-Dihydroxy-6,7,8-trimethoxyflavone 31.71 0.35 21 BAL8


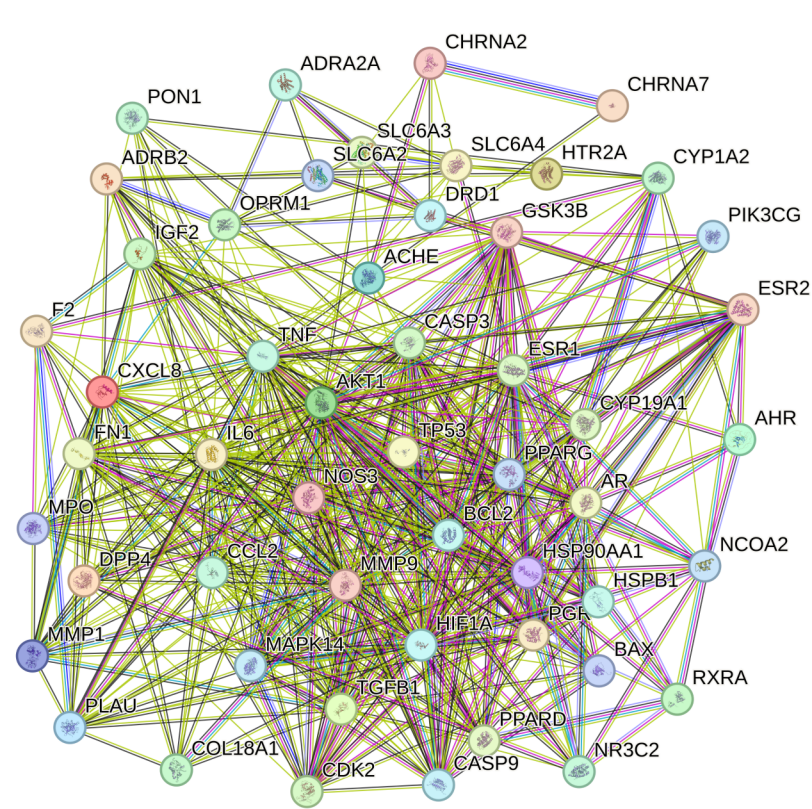


**Fig.S1 PPI Network of Scutellaria baicalensis in Treating Polycystic Ovary Syndrome：**Each node represents a potential therapeutic target of Scutellaria baicalensis for PCOS, while edges denote protein-protein interactions. The size and color intensity of a node correlate with the degree of the corresponding target protein, with larger and darker nodes indicating higher connectivity. Similarly, edge thickness reflects the strength of the interaction. (PPI: protein-protein interaction)。


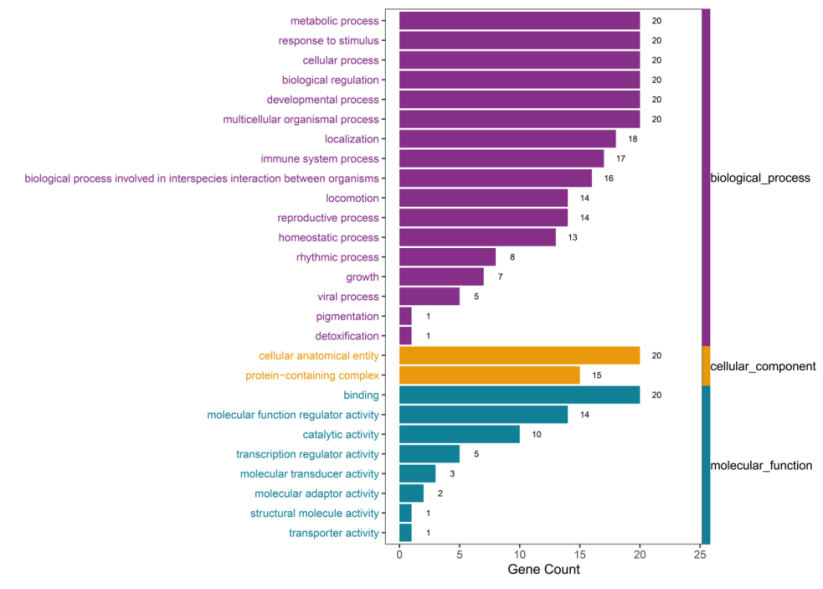


**Fig.S2 GO Enrichment Analysis：**This bar plot displays 27 significantly enriched GO terms associated with PCOS. The length of each bar represents the number of enriched genes, while bar colors correspond to the three major Gene Ontology categories: biological process, cellular component, and molecular function.


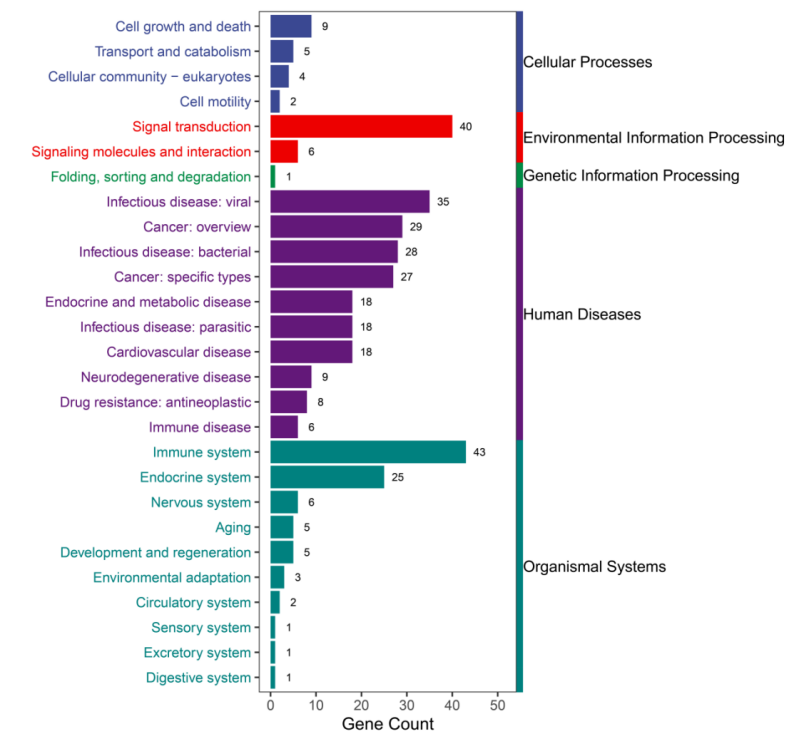


**Fig.S3 KEGG Enrichment Analysis：**This visualization displays 27 statistically significant KEGG pathways linked to PCOS. Horizontal bars denote distinct biological pathways, where bar dimensions reflect gene quantity enrichment levels. Chromatic variations represent functional categories including metabolic processes, immune responses, and hormonal regulation mechanisms.


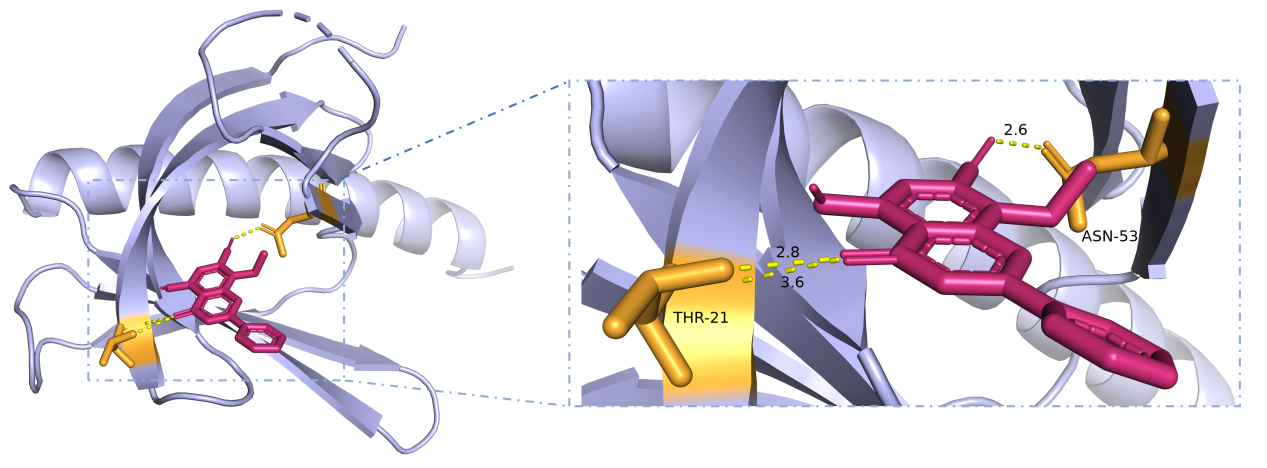


Wogonin&AKT1:△G=-7.0Kcal/mol


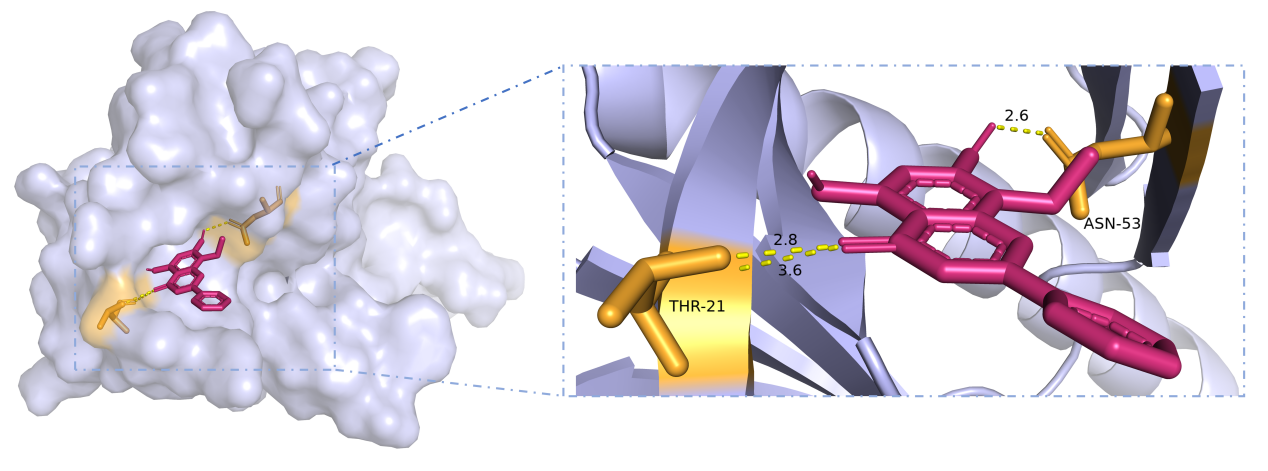


Wogonin&AKT1:△G=-7.0Kcal/mol

**Fig.S4** **Molecular Docking of *Baicalein* Active Constituent *Wogonin* with Key Protein *AKT1* and Corresponding Binding Free Energy (ΔG, kcal/mol):**The three-dimensional conformation demonstrates *wogonin's* molecular interaction with *AKT1*, emphasizing critical binding regions through spatial visualization. Computational analysis reveals a strong binding affinity between the compounds, quantified as -7.0 kcal/mol free energy value.


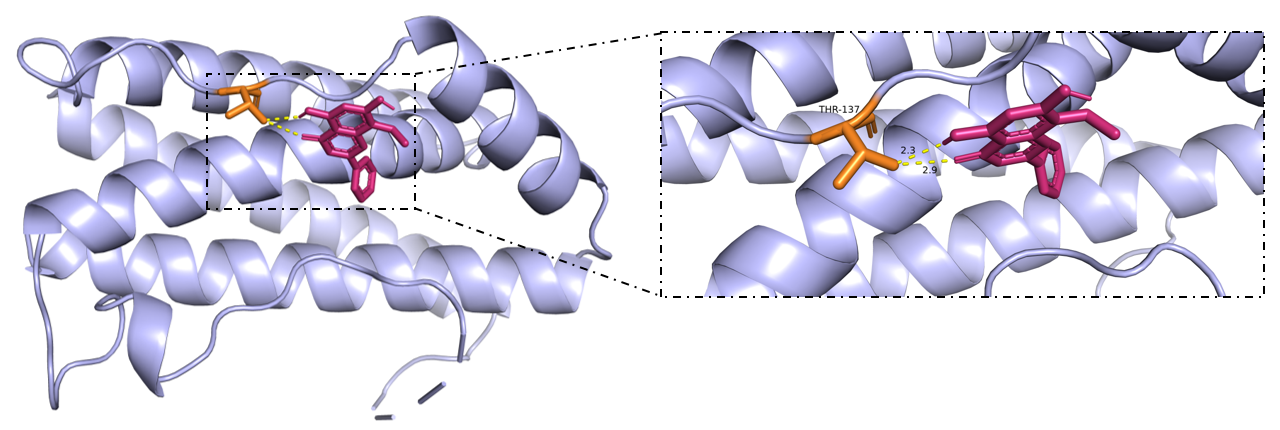


Wogonin&IL6:△G=-6.4Kcal/mol


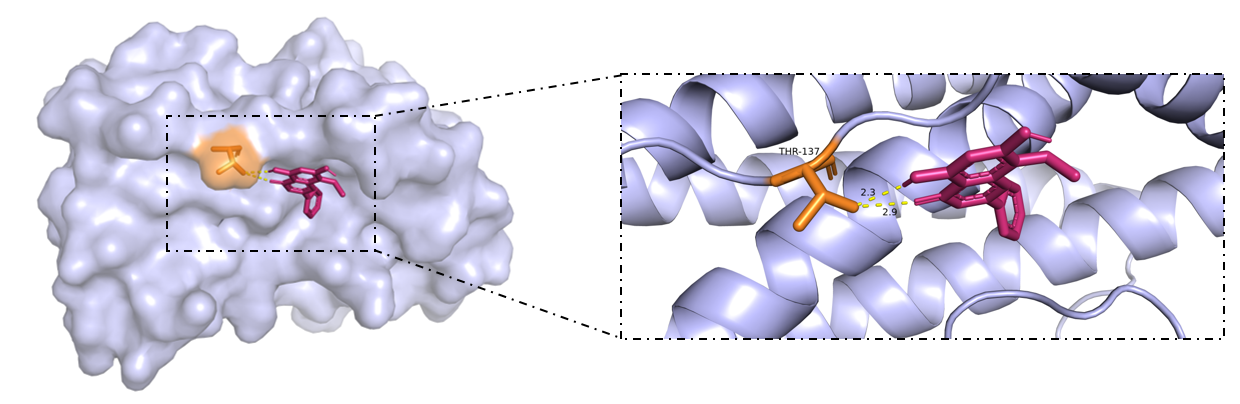


Wogonin&IL6:△G=-6.4Kcal/mol

**Fig.S5 Molecular Docking Analysis of *Baicalein's* Active Component *Wogonin* with *IL6* Protein and Associated Binding Energy (ΔG, kcal/mol):** The three-dimensional configuration illustrates the binding of *wogonin* to *IL6*, emphasizing critical interaction regions. Computational analysis reveals a binding free energy value of -6.4 kcal/mol between *wogonin* and *IL6*.


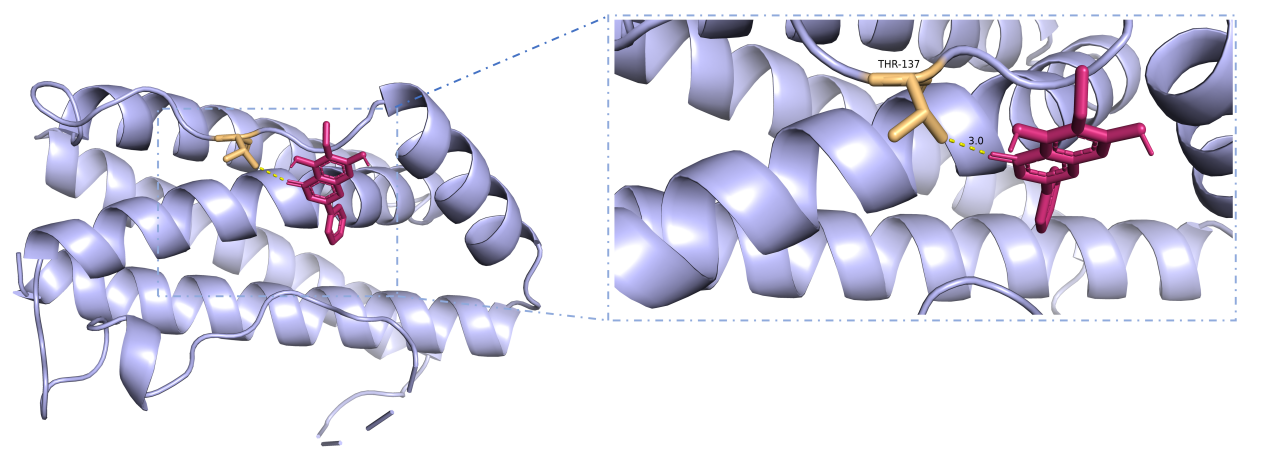


Oroxylin A&IL6:△G=-6.3Kcal/mol


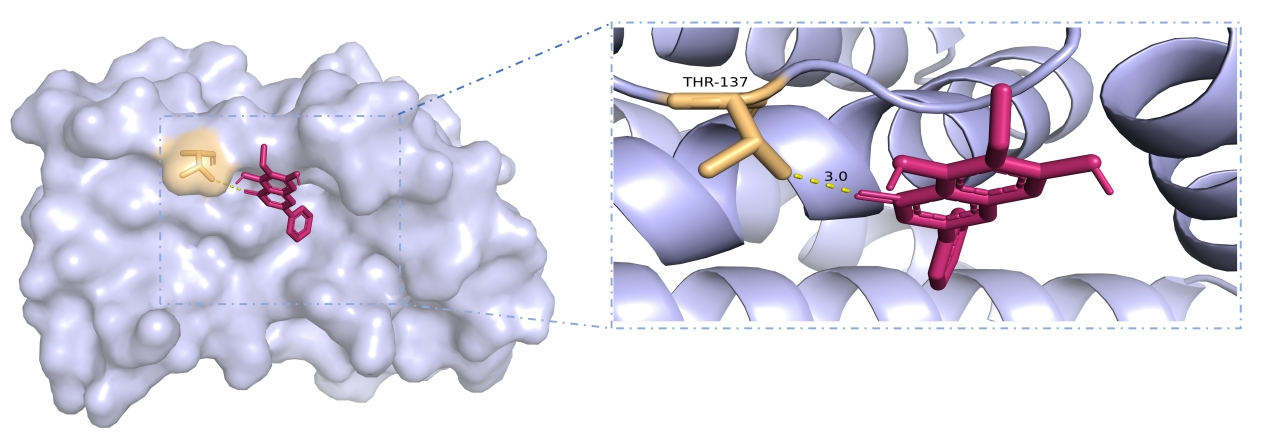


Oroxylin A&IL6:△G=-6.3Kcal/mol

**Fig.S6 Molecular Interaction Study of *Baicalein* Derivative *Oroxylin A* with *IL6* Protein and Corresponding Binding Energy (ΔG, kcal/mol):** Structural visualization demonstrates *Oroxylin A's* binding conformation with *IL6*, highlighting essential molecular contact points. The calculated interaction energy between *Oroxylin A* and *IL6* reaches -6.3 kcal/mol.

Tab S2 Pathological scoring criteria for rat colon tissue

Degree of inflammation Damage range Crypt destruction Affected proportion score

None None None 0% 0

Mild limited to the mucosal layer basal layer 1/3 1%-25% 1

Moderate mucosa and submucosa basal layer 2/3 26%-50% 2

Severe entire layer only surface epithelium intact 51%-75% 3

/ / both epithelial and basal 76%-100% 4

layers are destroyed


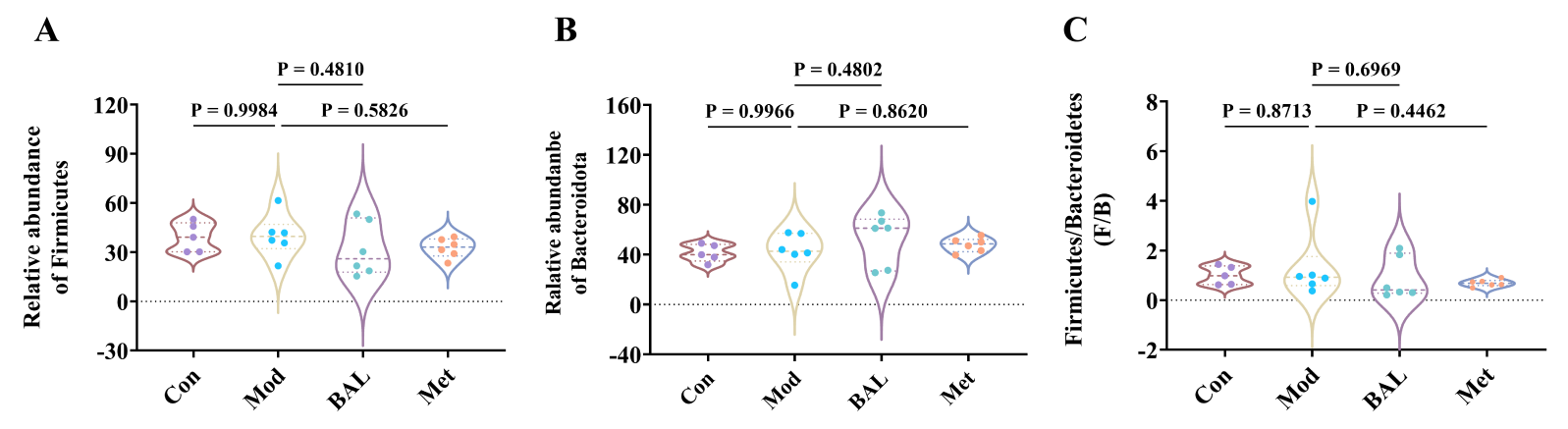


**Fig.S7** Relative abundance of *Firmicutes* and *Bacteroidetes* and their ratio (*F/B* ratio) in each group.


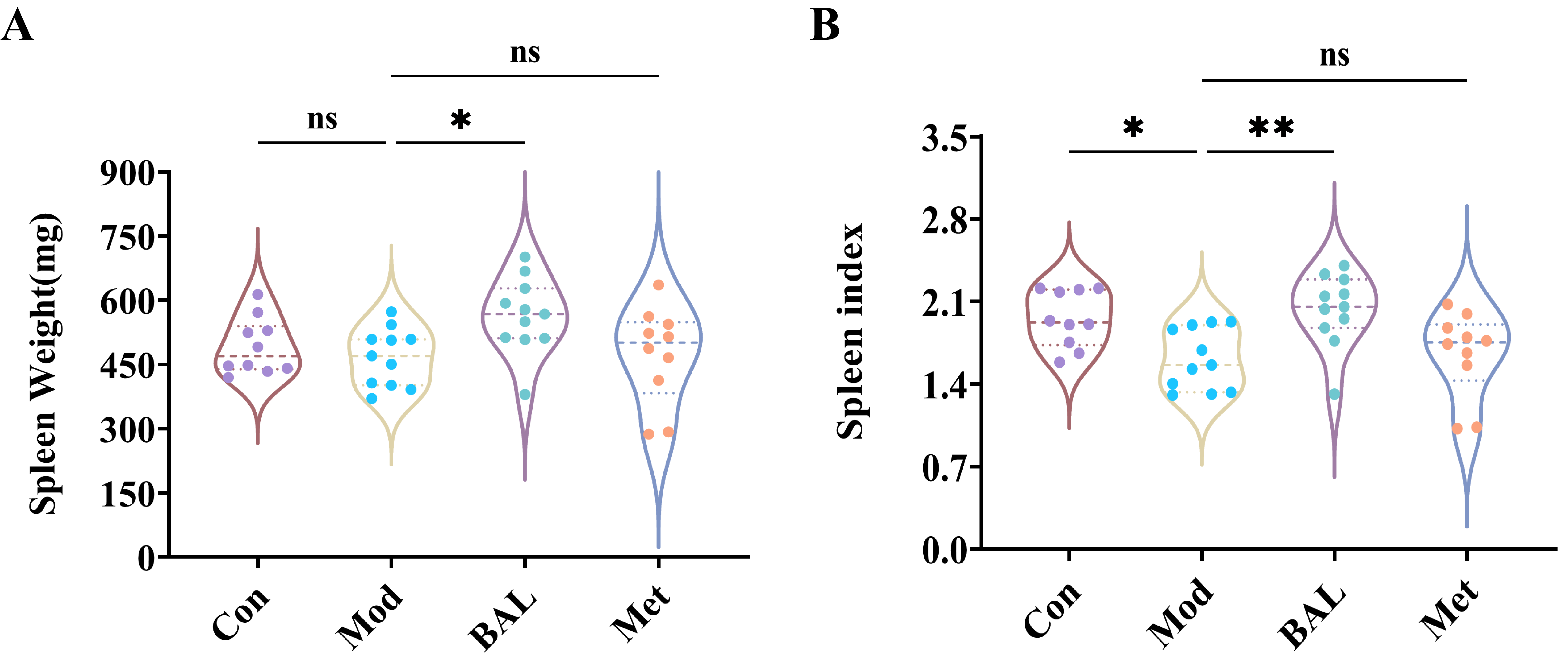


**Fig.S8** Quantitative analysis of spleen weight and spleen index.


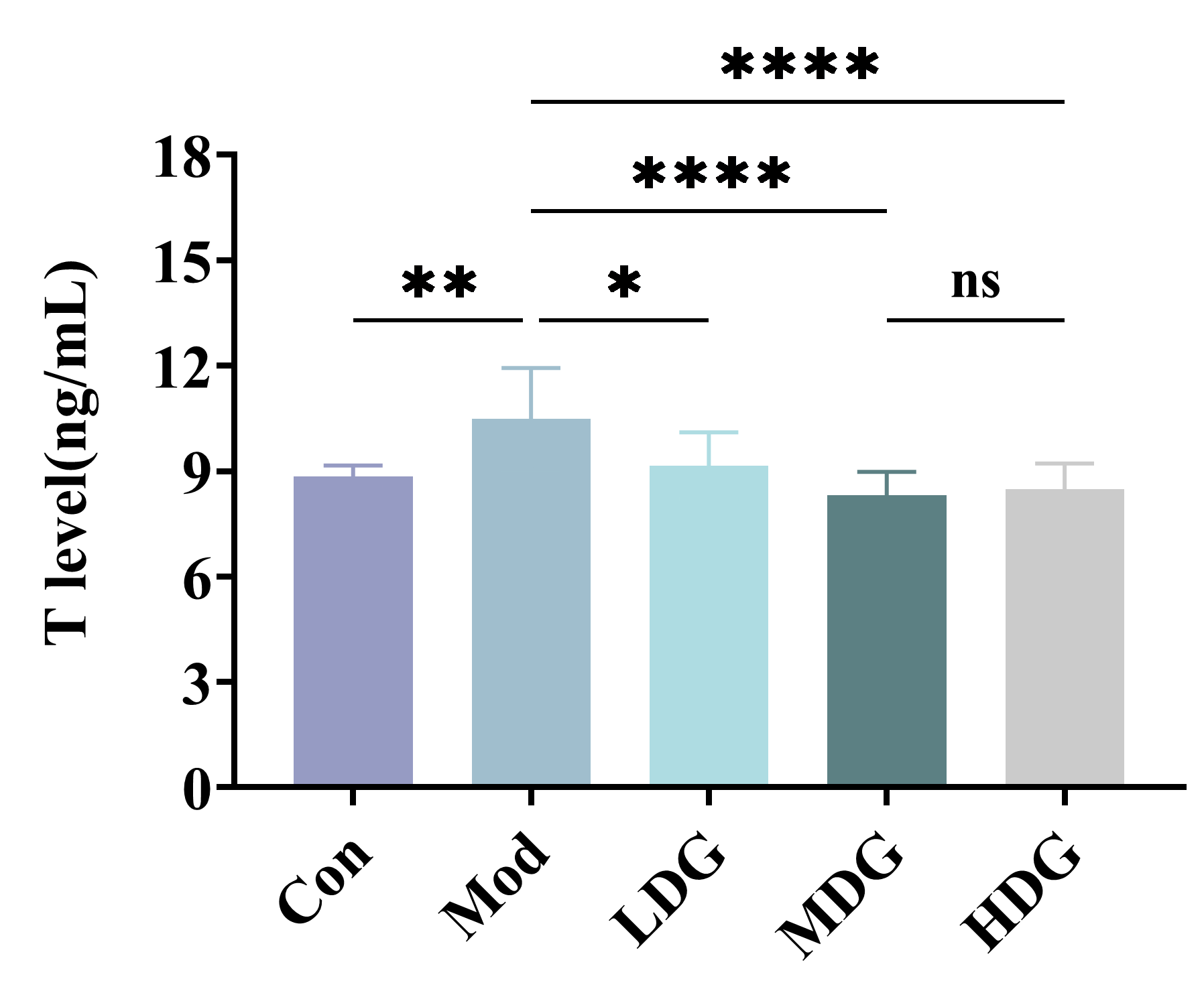


**Fig.S9** Effects of low (25 mg/kg), medium (50 mg/kg), and high (100 mg/kg) doses of *Scutellaria baicalensis* on serum testosterone levels.


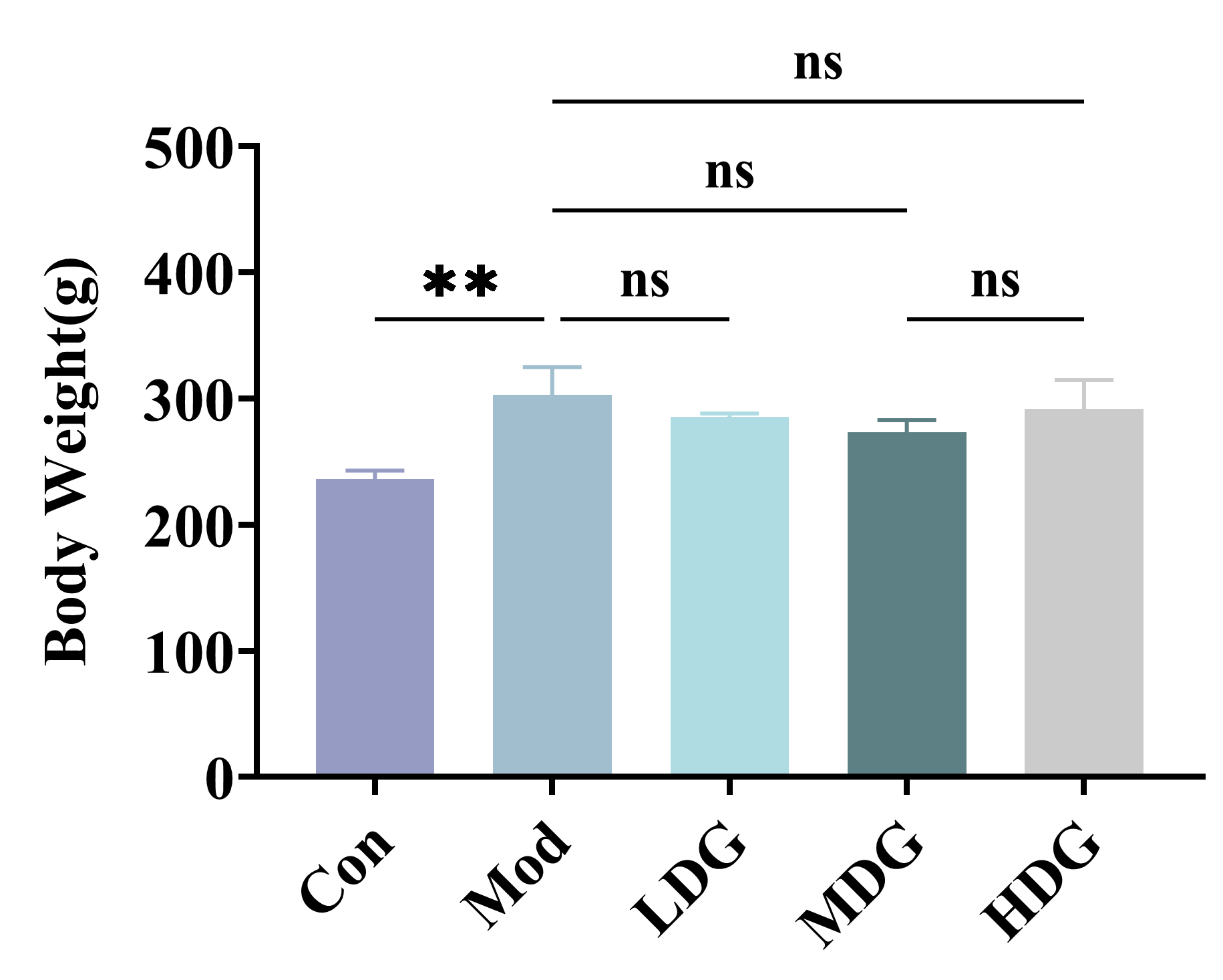


**Fig.S10** A comparison of body weight changes on day 22 across the groups showed that the Mod group had a significantly higher body weight than the Con group (*P < 0.01*). Compared with the Mod group, body weights in the low-dose (25 mg/kg), medium-dose (50 mg/kg), and high-dose (100 mg/kg) *Scutellaria baicalensis* groups all decreased, but the differences were not statistically significant (*P values* of *0.6391*, *0.2039*, and *0.9008*, respectively). Furthermore, although the body weight of rats in the medium-dose *Scutellaria baicalensis* group was lower than that in the high-dose group, the difference was not statistically significant (*P = 0.5921*).

**4 Discussion**

**Impact Analysis Study**

Based on the key findings of this study, we have conducted an in-depth analysis of its potential academic value and practical significance. The aim is to identify the target audience for this study, clarify its contribution to existing scientific questions, and point the way forward for future research. For a detailed impact analysis, please refer to the supplementary materials.

1. Target Beneficiary Group
   The findings of this study are expected to benefit various populations. First, for patients with PCOS who also suffer from metabolic disorders and immune dysregulation, this study revealed that *Scutellaria baicalensis* improved reproductive endocrine function by modulated the “gut-immune-ovarian” axis. Given that approximately 30% to 70% of PCOS patients exhibit varying degrees of chronic low-grade inflammation(12, 13)，This study provided a novel immunomodulatory intervention based on *Scutellaria baicalensis* for patients with PCOS. From the perspectives of reproductive endocrinology and traditional Chinese medicine, this study revealed new pathophysiological mechanisms underlying the heterogeneity of PCOS, laying a theoretical foundation for precision treatment integrating Western and traditional Chinese medicine. Furthermore, the TLR4/PI3K/AKT pathway and Phocaeicola-genus microbiota identified in this study may serve as potential targets and biomarkers for the development of PCOS drugs and gut microbes.

**2.** Potential contribution to existing scientific questions and clinical needs
This study explored the core issues of PCOS from multiple perspectives. At the mechanistic level, it not only validated the multi-target effects of *Scutellaria baicalensis* but also proposed, for the first time, the concept of “gut-immune-ovarian” axis dysregulation: LPS release caused by intestinal barrier damage serves as an upstream event driving systemic low-grade inflammation and ovarian dysfunction, integrating metabolic, immune, and reproductive phenotypes. At the therapeutic level, the study elucidated the complementary mechanisms of *Scutellaria baicalensis* and metformin—the latter focuses on improving metabolism, while the former focuses on restoring immune homeostasis and inhibiting inflammatory pathways. Although metformin, as a first-line drug, can increase ovulation rates by approximately 30%–50%, its gastrointestinal side effects and efficacy in non-obese PCOS patients remain controversial(14, 15)。For PCOS patients who respond poorly to metformin or exhibit marked immunological and inflammatory features, *Scutellaria baicalensis* or its compound formulations offer a unique therapeutic option, addressing the shortfall of existing treatment regimens that fail to cover all pathological subtypes. In diagnostic assessment, immune parameters such as the *Phocaeicola* index identified through fecal metagenomics, combined with spleen indices, can serve as novel biomarkers for evaluating the immune status and treatment response in PCOS, thereby facilitating precise subtyping and personalized treatment.

**3.** Future Research Directions Based on Current Findings
The findings of this study lay the groundwork for further exploration of underlying mechanisms and translational applications. Future research could focus on the following areas:

In-depth Mechanism Exploration and Causal Validation: This study has preliminarily elucidated the correlations and regulatory mechanisms. Future research should involve fecal microbiota transplantation experiments, in which the “healthy” microbiota enriched with *Phocaeicola* following *Scutellaria baicalensis* treatment is transplanted into germ-free or antibiotic-treated PCOS model rats, to validate the direct causal role of the gut microbiota in the therapeutic efficacy of *Scutellaria baicalensis*(16, 17)，In addition, tissue-specific knockout mice (such as ovarian granulosa cell-specific TLR4 knockout mice) can be used to further validate the critical role of the ovarian TLR4 signaling pathway in mediating the transition from systemic immune dysregulation to reproductive dysfunction.

Identification of Key Active Ingredients and Efficacy Evaluation: Although network pharmacology predicted that *Scutellaria baicalensis* contained multiple bioactive compounds, it remains to be determined which specific flavonoids or other compounds directly act on intestinal or ovarian targets in vivo. Future studies should employ techniques such as affinity chromatography-mass spectrometry to precisely identify the core active compounds in *Scutellaria baicalensis* that regulate the intestinal barrier, inhibit the TLR4 pathway, or promote the growth of *Phocaeicola*, and conduct pharmacodynamic validation.

Clinical Translation and Precision Medicine Research: Rigorously designed randomized, double-blind, placebo-controlled trials are needed to evaluate the efficacy and safety of *Scutellaria baicalensis* or its active monomers in specific PCOS subtypes (e.g., those with elevated LPS levels, reduced spleen index, or specific microbial profiles). Concurrently, baseline fecal samples should be prospectively collected from patients to validate the efficacy of *Phocaeicola* abundance as a biomarker for predicting treatment response to *Scutellaria baicalensis*, thereby laying the foundation for precision immunological interventions in PCOS.

**Limitation**

This study has certain limitations. Although the 5-week treatment period identified the core bacterial genus *Phocaeicola* and validated the early mechanisms by which *Scutellaria baicalensis* modulates the gut microbiota to improve PCOS phenotypes, it did not assess long-term effects. Future studies should extend the observation period and utilize multi-omics technologies to continuously monitor changes in the core microbiota and their impact on long-term host health, with the aim of providing more comprehensive evidence to support clinical translation.

**5 Conclusion**

First, Although this study preliminarily confirmed the efficacy of *Scutellaria baicalensis* in treating PCOS and explored its mechanisms of action, it did not quantitatively analyze the metabolism and distribution of active components in target tissues. It remained unclear whether major components such as *baicalin* and *baicalein* could reach key target organs, such as the ovaries and intestines, at effective concentrations, which limited the ability to elucidate their direct targets and pharmacological basis from a PK/PD perspective.

Second, The therapeutic effects of *Scutellaria baicalensis* may stem from the synergistic action of various flavonoid compounds. Although this study preliminarily confirmed its “multicomponent, multitarget” characteristics, it did not elucidate the synergistic or antagonistic relationships among the individual components. Future research should combine PK/PD analysis with combination index models to investigate the optimal ratios and interaction mechanisms of *Scutellaria baicalensis’s* active components in the treatment of PCOS, thereby integrating network-based predictions more closely with experimental validation.

1. Li YY, Peng YQ, Yang YX, Shi TJ, Liu RX, Luan YY, Yin CH. 2024. Baicalein improves the symptoms of polycystic ovary syndrome by mitigating oxidative stress and ferroptosis in the ovary and gravid placenta. Phytomedicine 128:155423.

2. Shah M-A, Park D-J, Kang J-B, Kim M-O, Koh P-O. 2019. Baicalin attenuates lipopolysaccharide-induced neuroinflammation in cerebral cortex of mice via inhibiting nuclear factor kappa B (NF-κB) activation. Journal of Veterinary Medical Science 81:1359-1367.

3. Xu X, Xu X, Wang X, Shen L. 2023. Baicalin suppress the development of polycystic ovary syndrome via regulating the miR-874-3p/FOXO3 and miR-144/FOXO1 axis. Pharm Biol 61:878-885.

4. Xu W, Tang M, Wang J, Wang L, Kennedy DA. 2020. Identification of the Active Constituents and Significant Pathways of Cangfu Daotan Decoction for the Treatment of PCOS Based on Network Pharmacology. Evidence-Based Complementary and Alternative Medicine 2020.

5. Ying Z, Hongqiu Z, Junjie L, Peijuan W, qian Z, Mengxue D, Yan L. 2026. Network pharmacology and molecular docking reveal the mechanism of Zishen Yutai Wan against polycystic ovary syndrome. Journal of Ovarian Research 19.

6. Ding J, Shanshan M, Mengcheng C, Danying Z, Jin Y. 2022. Integrated Network Pharmacology and Clinical Study to Reveal the Effects and Mechanisms of Bushen Huoxue Huatan Decoction on Polycystic Ovary Syndrome. Evid Based Complement Alternat Med 2022:2635375.

7. Hai B, Zhang Y, Huang J, Mprah R, Wang M. 2025. Exploring the key ingredients and mechanisms of Banxia Xiexin decoction for the treatment of polycystic ovary syndrome based on network pharmacology and experimental validation. Annals of Medicine 57.

8. Liu Y, Bai H, Guan H, Wang C, Song X, Yong Z, Guo X, Li L, Zhang Z. 2025. Animal experiments and network pharmacology to explore the anti-inflammatory mechanism of dapagliflozin in the treatment of polycystic ovary syndrome. Gynecological Endocrinology 41.

9. Ji Y, Han J, Lee N, Yoon J-H, Youn K, Ha HJ, Yoon E, Kim DH, Jun M. 2020. Neuroprotective Effects of Baicalein, Wogonin, and Oroxylin A on Amyloid Beta-Induced Toxicity via NF-κB/MAPK Pathway Modulation. Molecules 25.

10. Zhang L, Tian Y, Wang J, Deng S, Fan H. 2023. Network pharmacology-based research on the effect of Scutellaria baicalensis on osteosarcoma and the underlying mechanism. Medicine 102.

11. Zhu G, Zhang J, Yang Y, Zhang H, Jin W, Su F, Liang J, Wang K, Zhang J, Chen C. 2021. The Key Target and Molecular Mechanism of the Volatile Component of Scutellaria baicalensis Georgi in Acute Lung Injury Based on Network Pharmacology. Frontiers in Pharmacology 12.

12. Armanini D, Boscaro M, Bordin L, Sabbadin C. 2022. Controversies in the Pathogenesis, Diagnosis and Treatment of PCOS: Focus on Insulin Resistance, Inflammation, and Hyperandrogenism. International Journal of Molecular Sciences 23.

13. Rudnicka E, Suchta K, Grymowicz M, Calik-Ksepka A, Smolarczyk K, Duszewska AM, Smolarczyk R, Meczekalski B. 2021. Chronic Low Grade Inflammation in Pathogenesis of PCOS. International Journal of Molecular Sciences 22.

14. Juda A, Rojek K, Kamińska M, Strzoda AJ, Strzoda A, Sowiński WJ, Zdybel M, Strzoda A. 2025. Metformin in the Treatment of Polycystic Ovary Syndrome: A Literature Review. International Journal of Infertility & Fetal Medicine 16:186-190.

15. Teede HJ, Tay CT, Laven JJE, Dokras A, Moran LJ, Piltonen TT, Costello MF, Boivin J, Redman LM, Boyle JA, Norman RJ, Mousa A, Joham AE, Arlt W, Azziz R, Balen A, Bedson L, Berry L, Boivin J, Boyle J, Brennan L, Brown W, Burgert T, Busby M, Ee C, Garad RM, Gibson-Helm M, Harrison C, Hart R, Hopkins K, Hirschberg AL, Ho T, Hoeger K, Jordan C, Legro RS, Li R, Lujan M, Ma R, Mansfield D, Marsh K, Mocanu E, Mol B, Mormon R, Norman R, Oberfield S, Patel M, Pattuwage L, Peña A, Redman L, Rombauts L, et al. 2023. Recommendations from the 2023 international evidence-based guideline for the assessment and management of polycystic ovary syndrome. European Journal of Endocrinology 189:G43-G64.

16. Li P, Shuai P, Shen S, Zheng H, Sun P, Zhang R, Lan S, Lan Z, Jayawardana T, Yang Y, Zhao J, Liu Y, Chen X, El-Omar EM, Wan Z. 2023. Perturbations in gut microbiota composition in patients with polycystic ovary syndrome: a systematic review and meta-analysis. BMC Medicine 21.

17. Miller CB, Bader GA, Kay CL. 2026. Fecal Microbiota Transplantation in 2025: Two Steps Forward, One Step Back. Current Gastroenterology Reports 28.
