# Supplementary figures and images for: Targeting the gut-ovarian axis: Scutellaria baicalensis improves polycystic ovary syndrome by modulating gut microbiota composition and inhibiting the LPS/TLR4/NF-κB signaling pathway
Source: mSystems. 2026 Jun 15;11(7):e01825-25. doi: 10.1128/msystems.01825-25 (PMC13386983; doi:10.1128/msystems.01825-25)

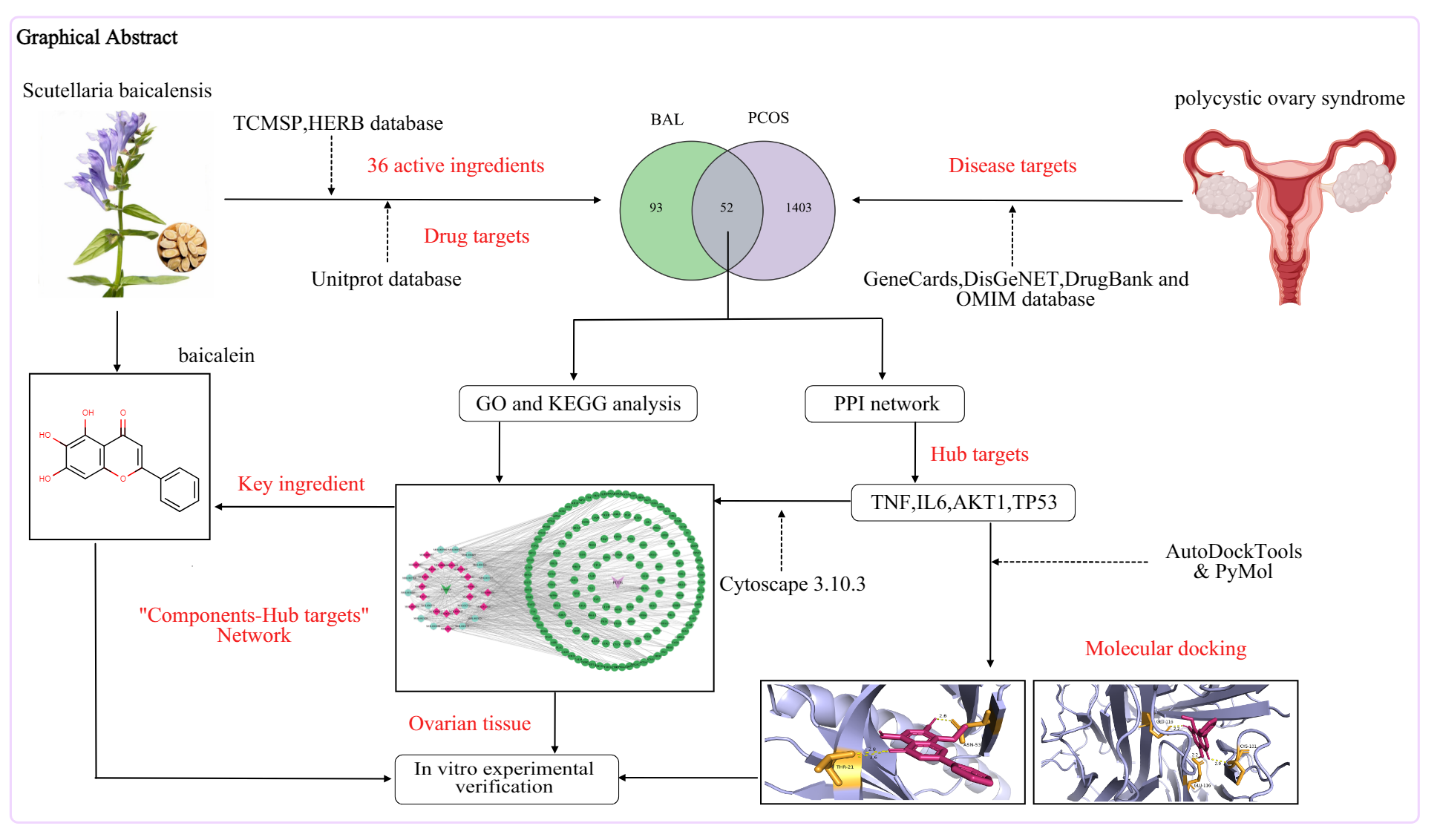

Supplement: Graphical abstract — Overview. [file msystems.01825-25-s0002.tif]
