# Supplementary material for: Targeting the gut-ovarian axis: Scutellaria baicalensis improves polycystic ovary syndrome by modulating gut microbiota composition and inhibiting the LPS/TLR4/NF-κB signaling pathway
Source: mSystems. 2026 Jun 15;11(7):e01825-25. doi: 10.1128/msystems.01825-25 (PMC13386983; doi:10.1128/msystems.01825-25)
Supplement: Reviewer comments [file reviewer-comments.pdf]

AMERICAN SOCIETY FOR MICROBIOLOGY | mSystems®

Targeting the Gut-Ovarian Axis: Scutellaria baicalensis Improves Polycystic Ovary Syndrome by Modulating Gut Microbiota Composition and Inhibiting the LPS/TLR4/NF-κB Signaling Pathway

Zhengxiu Pan, Caili Zhang, Qiangqiang Chu, Mingwei Chen, Cancan Hui, Tianjuan Wang, Maozhen Han, and Datong Deng

Corresponding Author(s): Zhengxiu Pan, The First Affiliated Hospital of Anhui Medical University

Review Timeline:

Submission Date:	December 24, 2025
Editorial Decision:	February 22, 2026
Revision Received:	April 19, 2026
Accepted:	May 15, 2026

Editor: Sangram Bagh

Reviewer(s): The reviewers have opted to remain anonymous.

Transaction Report:

(Note: With the exception of the correction of typographical or spelling errors that could be a source of ambiguity, letters and reports are not edited. The original formatting of letters and referee reports may not be reflected in this compilation.)

DOI: https://doi.org/10.1128/msystems.01825-25

Re: mSystems01825-25 (Targeting the Gut-Ovarian Axis: Scutellaria baicalensis Improves Polycystic Ovary Syndrome by Modulating Gut Microbiota Composition and Inhibiting the LPS/TLR4/NF-κB Signaling Pathway)

Dear Dr. Datong Deng:

Thank you for the privilege of reviewing your work. Below you will find my comments, instructions from the mSystems editorial office, and the reviewer comments.

Based on the reviewers' comments, this manuscript requires revision. Please address the points raised by the reviewers and revise the manuscript accordingly. A clear point-by-point response to all comments must be provided.

Please return the manuscript within 60 days; if you cannot complete the modification within this time period, please contact me. If you do not wish to modify the manuscript and prefer to submit it to another journal, notify me immediately so that the manuscript may be formally withdrawn from consideration by mSystems.

Revision Guidelines
To submit your modified manuscript, log into the submission site at https://msystems.msubmit.net/cgi-bin/main.plex. Go to Author Tasks and click the appropriate manuscript title to begin. The information you entered when you first submitted the paper will be displayed; update this as necessary. Note the following requirements:

• Upload point-by-point responses to the issues raised by the reviewers in a file named "Response to Reviewers," NOT in your cover letter.
• Upload a compare copy of the manuscript (without figures) as a "Marked-Up Manuscript" file.
• Upload a clean .DOC/.DOCX version of the revised manuscript and remove the previous version.
• Each figure must be uploaded as a separate, editable, high-resolution file (TIFF or EPS preferred), and any multipanel figures must be assembled into one file.
• Any supplemental material intended for posting by ASM should be uploaded with their legends separate from the main manuscript. You can combine all supplemental material into one file (preferred) or split it into a maximum of 10 files with all associated legends included.

For complete guidelines on revision requirements, see our Submission and Review Process webpage. Submission of a paper that does not conform to guidelines may delay acceptance of your manuscript.

Data availability: ASM policy requires that data be available to the public upon online posting of the article, so please verify all links to sequence records, if present, and make sure that each number retrieves the full record of the data. If a new accession number is not linked or a link is broken, provide mSystems production staff with the correct URL for the record. If the accession numbers for new data are not publicly accessible before the expected online posting of the article, publication may be delayed; please contact production staff (mSystems@asmusa.org) immediately with the expected release date.

Publication Fees: For information on publication fees and which article types are subject to charges, visit our website. If your manuscript is accepted for publication and any fees apply, you will be contacted separately about payment during the production process; please follow the instructions in that e-mail. Arrangements for payment must be made before your article is published.

ASM Membership: Corresponding authors may join or renew ASM membership to obtain discounts on publication fees. Need to upgrade your membership level? Please contact Customer Service at Service@asmusa.org.

The ASM Journals program strives for constant improvement in our submission and publication process. Please tell us how we can improve your experience by taking this quick Author Survey.

Thank you for submitting your paper to mSystems.

Sincerely,
Sangram Bagh
Editor
mSystems

Reviewer #1 (Comments for the Author):

This study integrates network pharmacology, animal experiments, and multi-omics analysis techniques to demonstrate that Scutellaria baicalensis exerts therapeutic effects in a polycystic ovary syndrome rat model by regulating the LPS/TLR4/NF-κB signaling pathway. The research systematically elucidates the interactions among the active components of S. baicalensis, key

gut microbes, and significant differentially expressed host genes, providing new evidence for the mechanism of action of traditional Chinese medicine in treating PCOS. The overall study design is innovative and holds significant scientific value in applying multi-omics technologies to decipher mechanisms of traditional Chinese medicine, aligning with the publication standards of mSystems. Authors are advised to further supplement and refine the manuscript based on the following aspects.

1. Lines 39, 40, 42, 44: Given that the abbreviation "BAL" has already been explicitly introduced, the repeated use of its full name "Scutellaria baicalensis" constitutes redundant expression. It is recommended to uniformly replace "Scutellaria baicalensis" with "BAL" in these instances to maintain terminological consistency and enhance textual conciseness.

2. The author analyzed the Firmicutes/Bacteroidetes (F/B) ratio in the Con, Mod, BAL, and Met groups within fecal metagenomics. However, the paper does not provide any figures illustrating this ratio data. It is recommended to supplement the manuscript with corresponding figures presenting the specific experimental results for the F/B ratio. This would provide more comprehensive data support for the relevant conclusions and enhance the verifiability of the paper.

3. The ruler shown in Fig. 5 is currently yellow, which differs from the ruler colors in other illustrations within the text. To maintain visual consistency across all figures and tables in the paper, it is recommended that the ruler color be adjusted to black.

4. When discussing the immune-related mechanisms by which Scutellaria baicalensis modulates symptoms in rats with polycystic ovary syndrome, the author mentions the role of the immune system. To further enhance the reliability and verifiability of the findings, it is recommended to supplement the experimental data with spleen weights and spleen indices for each group of rats, thereby providing more direct quantitative evidence of immune status.

5. This manuscript currently lacks a Highlights section. To emphasize the key innovations and findings of this study, it is recommended that this section be added.

6. The discussion section could further deepen the practical value of the research. It is recommended to include a section addressing the study's impact, covering: 1. The target beneficiaries of this research (e.g., clinicians or specific patient groups); 2. The potential contributions to resolving existing scientific questions or clinical needs; 3. Clear directions for future research based on the current findings. Incorporating these elements would enhance the paper's academic influence and practical guidance.

Reviewer #2 (Comments for the Author):

The authors used several techniques to investigate the therapeutic effects of Scutellaria baicalensis exerts for PCOS, and try to illustrate the depth mechanism, which involved in the LPS/TLR4/NF-κB signaling pathway. The quality of this article is ok. However, several problems should be solve and the the comments as follows.

1. As mentioned in the manuscript, the authors determined the intervention dose of Scutellaria baicalensis, namely, 50 mg/kg/day, why? Was the efficacy and safety of this dose validated through preliminary experiments (e.g., dose-gradient experiments: 25, 50, 100 mg/kg)? Without multiple dose groups, how were dose-dependent effects ruled out or the optimal therapeutic dose confirmed?
2. Was the dose for the metformin control group (270 mg/kg/day) determined with reference to the body surface area conversion standard between rats and humans? Is this dose supported by previous literature regarding its efficacy in PCOS rat models?
3. Molecular docking was performed with only three core active compounds (wogonin, baicalein, and oroxylin A) and three targets (TNF, IL6, AKT1), without explaining why other potential targets were excluded. Is the rationale for target selection sufficiently justified?
4. The in vivo metabolic fate of the active compounds from Scutellaria baicalensis remains unclear. Were the concentrations of these compounds measured in serum, ovarian, or intestinal tissues to confirm their ability to reach the target sites and achieve effective concentrations? Were potential synergistic or antagonistic interactions among the compounds considered?
5. The intervention period lasted merely 5 weeks. No assessments were performed on long-term outcomes (e.g., the recovery of fertility in rats, offspring growth) or long-term safety (e.g., hepatic and renal function, sustained remodeling of the gut microbiota). Can the results of short-term intervention adequately reflect the efficacy of long-term clinical administration?
6. There are significant differences in the composition of the gut microbiota between humans and rats. In the present study, is there any supporting literature in human patients with polycystic ovary syndrome (PCOS) for the regulatory effects of Scutellaria baicalensis on the abundance of Phocaeicola? How can the translational gaps between animal models and clinical applications in humans be addressed?
7. The study indicates that the genus Phocaeicola is a core gut microbiota component regulated by Scutellaria baicalensis, but its specific mechanism of action remains unclear. Does Phocaeicola contribute to intestinal barrier repair by producing metabolites such as short-chain fatty acids or bile acids? Or does it directly interact with intestinal epithelial cells to inhibit LPS release?
8. In microbiome analysis, when no significant differences were observed in α-diversity (Shannon and Simpson indices) but distinct significant differences existed in β-diversity, how to interpret the phenomenon of "unchanged species richness yet

remodeling of microbial community structure"? Could this be attributed to the selective regulation of specific functional microbial taxa by Scutellaria baicalensis?

81 Meishan Road, Hefei, Anhui 230032, China

April 6, 2026

Editorial Staff, ***mSystems***
Dear editor and reviewers,

In response to your decision on our submission of "**Targeting the Gut-Ovarian Axis: *Scutellaria baicalensis* Improves Polycystic Ovary Syndrome by Modulating Gut Microbiota Composition and Inhibiting the *LPS/TLR4/NF-κB* Signaling Pathway**" to *mSystems* (Manuscript Number: **mSystems01825-25**).

Thank you for considering our manuscript and providing insightful comments and suggestions, which have not only made our present work scientifically more accurate and sounder, but also helped to improve its readability to a broad audience. Here we are submitting the revision of the manuscript, along with this responding letter addressing the suggestions.

We have modified our manuscript, especially for the issues raised by reviewers, and carefully checked and proof-read it again to make the descriptions to be more precise. Again, thank you and the anonymous reviewers for the tremendous efforts helping us improve our manuscript.

We guarantee any part of the paper has not been published or is under consideration for publication elsewhere, including on the Internet.

We thank you for your time and consideration, and look forward to future correspondence.

We hope you will find our updated manuscript to be suitable for *mSystems*. Thank you again for the valuable and constructive suggestion!

Yours Sincerely

Best Regards

Datong Deng, Maozhen Han, Tianjuan Wang, Zhengxiu Pan, and Caili Zhang

Responses to comments of Editor and Reviewers:

Editors:

Answer: We sincerely appreciate the constructive feedback from the reviewers, which has significantly strengthened our manuscript. Below, we address each comment in detail and outline the revisions made to the manuscript and supplementary materials. All changes are highlighted in the revised manuscript (track-changes mode) and supplementary files.

Reviewer #1 (Comments for the Author):

1.Lines 39,40,42,44:Given that the abbreviation "BAL" has already been explicitly introduced, the repeated use of its full name "Scutellaria baicalensis" constitutes redundant expression. It is recommended to uniformly replace "Scutellaria baicalensis" with "BAL" in these instances to maintain terminological consistency and enhance textual conciseness.

Answer: Thank you for your valuable and constructive comments.

Thank you very much for your valuable feedback. Regarding the terminology issues you pointed out in lines 39, 40, 42, and 44, we have carefully checked them. Since "*BAL*" has been explicitly introduced as the abbreviation for "*Scutellaria baicalensis*" in the text, to avoid repetition and ensure consistency and conciseness throughout the manuscript, we agreed with and adopt your suggestion. As requested, we have uniformly replaced "*Scutellaria baicalensis*" with "*BAL*" in the aforementioned lines. Please check, thanks.

2.The author analyzed the Firmicutes/Bacteroidetes (F/B) ratio in the Con,Mod,BAL, and Met groups within fecal metagenomics. However, the paper does not provide any figures illustrating this ratio data. It is recommended to supplement the manuscript with corresponding figures presenting the specific experimental results for the F/B ratio. This would provide more comprehensive data support for the relevant conclusions and enhance the verifiability of the paper.

Answer: We sincerely appreciate your valuable suggestions. We fully agreed that adding figures for *Firmicutes*, *Bacteroidetes*, and their ratio (*F/B* ratio) in the revised manuscript will more intuitively reveal the differences among the groups (Con, Mod, BAL, Met), further strengthen the data support for our conclusions, and enhance the verifiability of our findings. Accordingly, we have made the following additions in the revised manuscript: (1) Supplementary Figure S7 has been added to the Supplementary Materials, specifically presenting the *F/B* ratios for the four groups (*ANOVA*, $P > 0.05$). (2) We have added description at line 491 in the main text (Supplementary Figure S7).

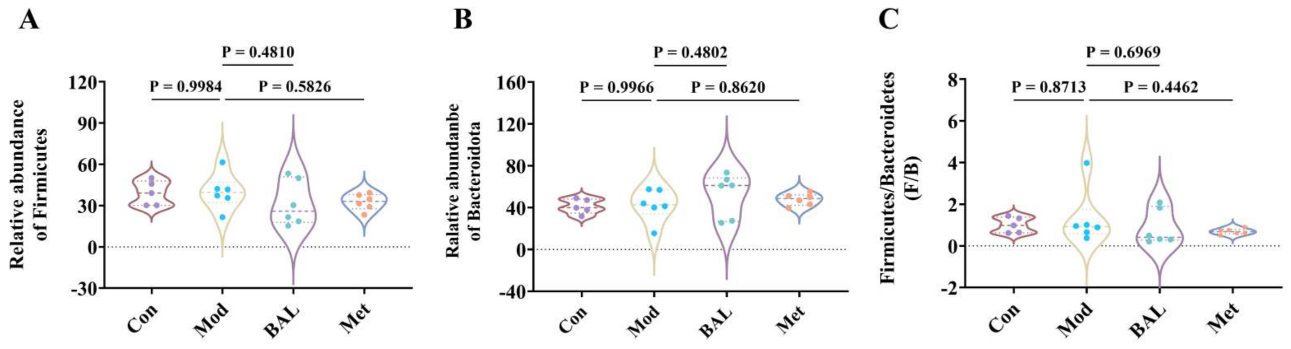

Fig.S7 Relative abundance of *Firmicutes* and *Bacteroidetes* and their ratio (*F/B* ratio) in each group.

3. The ruler shown in Fig.5 is currently yellow, which differs from the ruler colors in other illustrations within the text. To maintain visual consistency across all figures and tables in the paper, it is recommended that the ruler color be adjusted to black.

Answer: Thank you for pointing out the inconsistent ruler colors in Figure 5. We have revised the relevant images and adjusted the ruler colors to black to ensure consistency with the style of figures throughout the manuscript. The revised Figure 5 has been replaced in the revised draft. Please check.

4. When discussing the immune-related mechanisms by which Scutellaria baicalensis modulates symptoms in rats with polycystic ovary syndrome, the author mentions the role of the immune system. To further enhance the reliability and verifiability of the findings, it is recommended to supplement the experimental data with spleen weights and spleen inxices for each group of rats, thereby providing more direct quantitative evidence of immune status.

Answer: We sincerely appreciate your valuable suggestions. We fully agreed with your recommendation to supplement spleen weight and spleen index as direct evidence of immune status. As a key peripheral immune organ, spleen weight and its index can directly reflect the body's immune status, providing more robust experimental evidence for how *Scutellaria baicalensis* improves polycystic ovary syndrome (PCOS) through immune mechanisms. Following your recommendation, we have calculated the spleen index (spleen weight/body weight) for rats in each group. The specific modifications are as follows:

(1). In Results section: On line 439, statistical results for spleen weight and spleen index of each group have been added. We examined the effects of *BAL* on spleen weight and spleen index in PCOS rats. Results showed that the spleen index in the model group was significantly lower than that in the control group (*ANOVA*, $P < 0.05$), indicating immunosuppression under PCOS conditions. Although spleen weight decreased, no statistically significant difference was observed. Following *BAL* intervention, both spleen

weight (*ANOVA, P < 0.05*) and spleen index (*ANOVA, P < 0.01*) significantly recovered compared to the model group, whereas the Met group showed no notable changes. These findings indicated that *BAL* effectively ameliorates immune abnormalities in PCOS rats (Supplementary Figure S8).

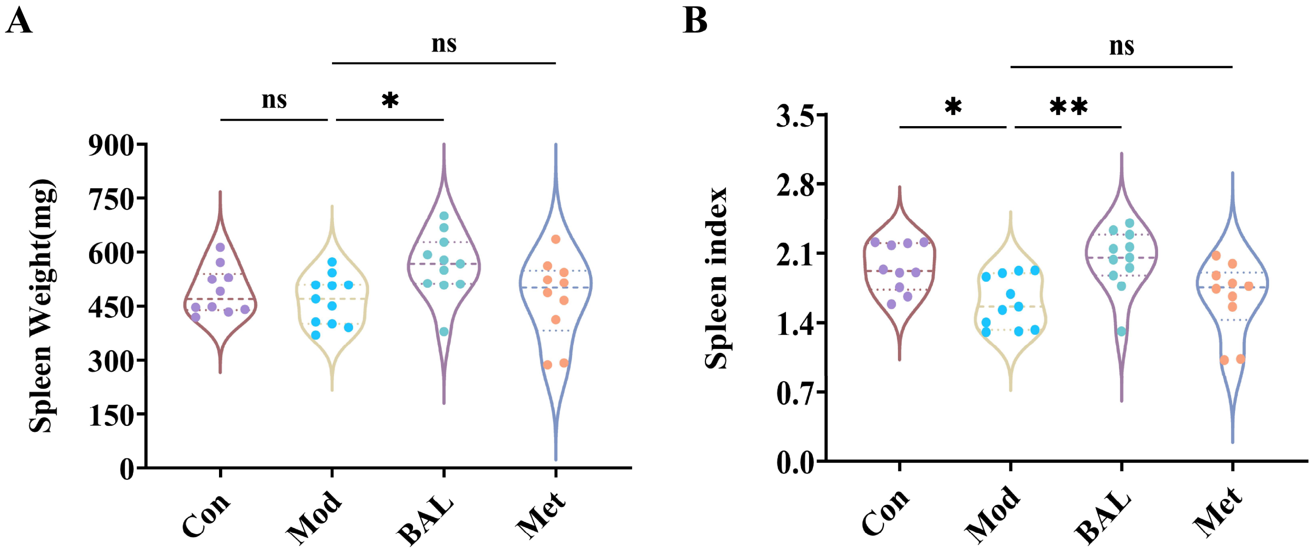

Fig.S8 Quantitative analysis of spleen weight and spleen index for four groups.

(2). In Animal Experiment Design Section: On line 227, we have added the spleen processing procedure: Dissect and isolate the spleen, remove connective tissue, rinse with PBS, blot dry with filter paper, weigh, and calculate the spleen index [Spleen Index = Spleen Weight (mg) / Body Weight (g)].

(3). In Discussion section: On line 612, we have added additional analysis: In the pathological context of PCOS, gut-derived LPS may induce a systemic inflammatory cascade by persistently activating the *TLR4/NF-κB* signaling pathway. This process has exacerbated lymphocyte apoptosis within splenic tissue while suppressing its regenerative capacity, ultimately leading to splenic atrophy and compromised immune function. Furthermore, elevated inflammatory factors not only contribute to insulin resistance but also increase free testosterone levels by suppressing hepatic synthesis of sex hormone-binding globulin or directly stimulating ovarian theca cells to produce androgens. This inhibits follicular growth and may even cause follicular atresia. On line 620: Additionally, In PCOS rats, spleen weight exhibited a decreasing trend without statistical significance (*ANOVA, P > 0.05*), whereas the spleen index was significantly reduced (*ANOVA, P < 0.05*), suggesting impaired systemic immune function. On line 622: Effectively reduced inflammatory factor levels in serum and ovarian tissue, improved colonic tight junction protein expression and mucosal structure, restored spleen index, and suppressed abnormal expression of the *TLR4/PI3K/AKT/NF-κB* p65 signaling axis in ovarian tissue. suggesting that *BAL* may alleviate immune dysregulation in PCOS rats by targeting the *LPS/TLR4/NF-κB* pathway, suppressing its excessive activation,

thereby restoring immune homeostasis, modulating the local ovarian inflammatory microenvironment, and improving ovarian function.

5. This manuscript currently lacks a Highlights section. To emphasize the key innovations and findings of this study, it is recommended that this section be added.

Answer: Thank you very much for your valuable suggestion. We fully agree on the importance of highlighting the key innovations and major findings of the study. However, it should be noted that according to the official submission guidelines of *mSystems*, the journal does not require a "Highlights" section, but instead recommends an "Importance" paragraph to summarize the significance and main discoveries of the study. In fact, in the original manuscript, we had already written an "Importance" section in strict accordance with the journal's requirements. If any confusion arose due to the formatting not being sufficiently conspicuous, we sincerely apologize and are willing to adjust the presentation as suggested by the editorial office. Once again, thank you for your careful and constructive feedback.

6. The discussion section could further deepen the practical value of the research.It is recommended to include a section addressing the study's impact, covering: 1. The target beneficiaries of this research (e.g., clinicians or specific patient groups); 2. The potential contributions to resolving existing scientific questions or clinical needs; 3.Clear directions for future research based on the current findings. Incorporating these elements would enhance the paper's academic influence and practical guidance.

Answer: We sincerely appreciate your valuable suggestions. As requested, we have added a subsection titled "Impact Analysis" to the "Discussion" section of the supplementary materials. This subsection outlined the target beneficiary groups, the potential contribution to existing scientific or clinical issues, and future research directions. The aim is to enhance the academic impact and practical guidance value of this paper.

We have supplemented the relevant content in the section titled 'Impact Analysis Study', starting from line 647 of the manuscript.

Impact Analysis Study

Based on the key findings of this study, we have conducted an in-depth analysis of its potential academic value and practical significance. The aim is to identify the target audience for this study, clarify its

contribution to existing scientific questions, and point the way forward for future research. For a detailed impact analysis, please refer to the supplementary materials.

(1) Target Beneficiary Group

The findings of this study are expected to benefit various populations. First, for patients with PCOS who also suffer from metabolic disorders and immune dysregulation, this study revealed that *Scutellaria baicalensis* improved reproductive endocrine function by modulated the "gut-immune-ovarian" axis. Given that approximately 30% to 70% of PCOS patients exhibit varying degrees of chronic low-grade inflammation**(1, 2),** This study provided a novel immunomodulatory intervention based on *Scutellaria baicalensis* for patients with PCOS. From the perspectives of reproductive endocrinology and traditional Chinese medicine, this study revealed new pathophysiological mechanisms underlying the heterogeneity of PCOS, laying a theoretical foundation for precision treatment integrating Western and traditional Chinese medicine. Furthermore, the *TLR4/PI3K/AKT* pathway and Phocaeicola-genus microbiota identified in this study may serve as potential targets and biomarkers for the development of PCOS drugs and probiotics.

(2) Potential contribution to existing scientific questions and clinical needs

This study explored the core issues of PCOS from multiple perspectives. At the mechanistic level, it not only validated the multi-target effects of *Scutellaria baicalensis* but also proposed, for the first time, the concept of "gut-immune-ovarian" axis dysregulation: LPS release caused by intestinal barrier damage serves as an upstream event driving systemic low-grade inflammation and ovarian dysfunction, integrating metabolic, immune, and reproductive phenotypes. At the therapeutic level, the study elucidated the complementary mechanisms of *Scutellaria baicalensis* and metformin—the latter focuses on improving metabolism, while the former focuses on restoring immune homeostasis and inhibiting inflammatory pathways. Although metformin, as a first-line drug, can increase ovulation rates by approximately 30%–50%, its gastrointestinal side effects and efficacy in non-obese PCOS patients remain controversial(3, 4). For PCOS patients who respond poorly to metformin or exhibit marked immunological and inflammatory features, *Scutellaria baicalensis* or its compound formulations offer a unique therapeutic option, addressing the shortfall of existing treatment regimens that fail to cover all pathological subtypes. In diagnostic assessment, immune parameters such as the *Phocaeicola* index identified through fecal metagenomics, combined with spleen indices, can serve as novel biomarkers for evaluating the immune status and treatment response in PCOS, thereby facilitating precise subtyping and personalized treatment.

(3) Future Research Directions Based on Current Findings

The findings of this study lay the groundwork for further exploration of underlying mechanisms and translational applications. Future research could focus on the following areas:

In-depth Mechanism Exploration and Causal Validation: This study has preliminarily elucidated the correlations and regulatory mechanisms. Future research should involve fecal microbiota transplantation experiments, in which the “healthy” microbiota enriched with *Phocaeicola* following *Scutellaria baicalensis* treatment is transplanted into germ-free or antibiotic-treated PCOS model rats, to validate the direct causal role of the gut microbiota in the therapeutic efficacy of *Scutellaria baicalensis*(5, 6)，In addition, tissue-specific knockout mice (such as ovarian granulosa cell-specific TLR4 knockout mice) can be used to further validate the critical role of the ovarian TLR4 signaling pathway in mediating the transition from systemic immune dysregulation to reproductive dysfunction.

Identification of Key Active Ingredients and Efficacy Evaluation: Although network pharmacology predicted that *Scutellaria baicalensis* contained multiple bioactive compounds, it remains to be determined which specific flavonoids or other compounds directly act on intestinal or ovarian targets in vivo. Future studies should employ techniques such as affinity chromatography-mass spectrometry to precisely identify the core active compounds in *Scutellaria baicalensis* that regulate the intestinal barrier, inhibit the TLR4 pathway, or promote the growth of *Phocaeicola*, and conduct pharmacodynamic validation.

Clinical Translation and Precision Medicine Research: Rigorously designed randomized, double-blind, placebo-controlled trials are needed to evaluate the efficacy and safety of *Scutellaria baicalensis* or its active monomers in specific PCOS subtypes (e.g., those with elevated LPS levels, reduced spleen index, or specific microbial profiles). Concurrently, baseline fecal samples should be prospectively collected from patients to validate the efficacy of *Phocaeicola* abundance as a biomarker for predicting treatment response to *Scutellaria baicalensis*, thereby laying the foundation for precision immunological interventions in PCOS.

Reviewer #2 (Comments for the Author):

The authors used several techniques to investigate the therapeutic effects of Scutellaria baicalensis exerts for PCOS, and try to illustrate the depth mechanism,which involved in the LPS/TLR4/NF-κB signaling pathway. The quality of this article is ok. However, several problems should be solve and the comments as follows.

Answer: We are deeply grateful to you for recognizing the value of our research. We fully accept and take seriously the specific issues raised, as these comments provide crucial guidance for enhancing the scientific

rigor and academic quality of our manuscript. We hereby addressed each comment point by point and tracked the corresponding revisions made in the manuscript. All modifications have been highlighted in the manuscript and supplementary materials.

1. As mentioned in the manuscript, the authors determined the intervention dose of Scultellaria baicalensis, namely, 50mg/kg/day, why? Was the efficacy and safety of this dose validated through preleminary experiments (e.g., dose-gradient experiments: 25, 50, 100mg/kg)? Without multiple dose groups, how were dose-dependent effects ruled out or the optimal therapeutic dose confirmed?

Answer: We are very grateful to you for your suggestions for further revisions to our manuscript. Your inquiry regarding the rationale for selecting the dosage of *Scutellaria baicalensis* is both professional and insightful, and it is crucial for ensuring the scientific rigor and reproducibility of our study's conclusions. In accordance with your recommendations, we have verified the relevant issues, conducted supplementary analyses, and made corresponding revisions to the manuscript. Our specific responses are outlined below.

We fully agreed with the reviewer's view that, in research on natural products such as traditional Chinese medicine, establishing the scientific basis for dose selection and verifying the dose-response relationship are critical steps in elucidating their pharmacological effects and determining the optimal therapeutic dose. Regarding the intervention dose of 50 mg/kg/day selected in our manuscript, we provide the following detailed explanation and supplementary information from three perspectives:

(1) Literature Basis for Dose Selection:

In this study, the dosage of *Scutellaria baicalensis* was determined based on the effective range previously validated in rodent disease models, and took into account evidence regarding the regulation of the *TLR4/NF-κB* pathway by *Scutellaria baicalensis* and *baicalin*.

A systematic review indicates that the effective oral dose of *Scutellaria baicalensis* (or its extract) in the aforementioned models is 20–100 mg/kg/day, and that it exhibits anti-inflammatory and metabolic regulatory effects. Specifically:

Regarding PCOS models, Xu et al (7), in a DHEA-induced PCOS rat model, *baicalin* (20 mg/kg, administered orally for 4 weeks) significantly reduced free testosterone and luteinizing hormone levels and improved the phenotype; the mechanism involves regulation via the miR-874-3p/FOXO3 and miR-144/FOXO1 axes.

Regarding the anti-inflammatory mechanism, a recent study by Li et al (8) demonstrated, using molecular docking and experimental studies, that *baicalin* can directly bind to the active site of the *TLR4/MD2* complex, thereby inhibiting the *PI3K/AKT/NF-κB* pathway and reducing *TNF-α/IL-1β* levels. This is consistent with the *LPS/TLR4/NF-κB* pathway, which is the focus of this study, and provides direct molecular evidence for the anti-inflammatory mechanism by which *Scutellaria baicalensis* targets this pathway. In an LPS-induced neuroinflammatory mouse model, Shah et al (9) demonstrated that *baicalin* (50 and 80 mg/kg) significantly reduced inflammatory cytokine levels by inhibiting *NF-κB* activation, thereby validating the anti-inflammatory efficacy of the 50 mg/kg dose.

Based on the literature, this study used the median dose (50 mg/kg/day) as the initial intervention dose; see the Supplementary Materials for details.

(2) Validation of Dose Efficacy and Safety Based on Preliminary Experiment Results:

We fully recognize the importance of preliminary experiments in determining the final protocol. Prior to the formal experiment, we conducted preliminary studies on the efficacy and toxicity of different doses of *Scutellaria baicalensis* in a rat PCOS model.

Dose gradient design: In rats with a PCOS model ($n = 3$ per group), *Scutellaria baicalensis* was administered orally at doses of 25, 50, and 100 mg/kg/day (low, medium, and high dose groups) for 21 consecutive days. A model group and a control group were established simultaneously.

Efficacy Assessment: Preliminary evaluations indicated that, compared with the control group, the 25, 50, and 100 mg/kg dose groups all significantly improved the PCOS-like phenotype, as evidenced by the restoration of the estrous cycle and reduced serum testosterone levels (Fig. S9), with the 50 mg/kg group showing the most significant improvement; there were no statistically significant differences between the 50 and 100 mg/kg groups across all parameters.

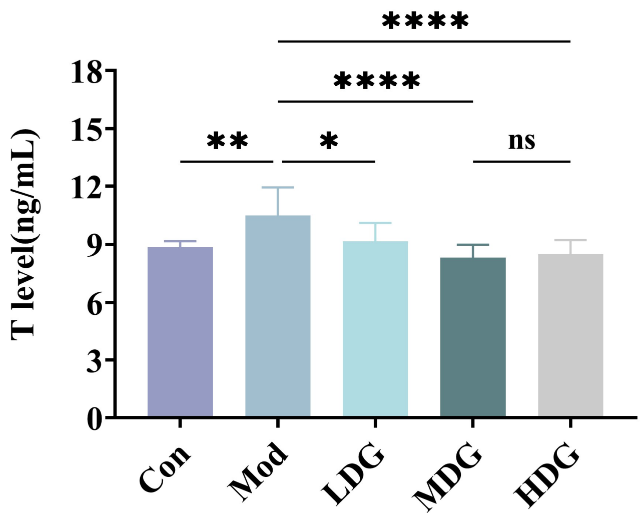

Fig.S9 Effects of low (25 mg/kg), medium (50 mg/kg), and high (100 mg/kg) doses of *Scutellaria baicalensis* on serum testosterone levels.

Safety Assessment: Throughout the intervention period, no deaths, behavioral abnormalities, or significant weight loss were observed in rats in any of the dose groups (Fig. S10). Based on these observable parameters, *Scutellaria baicalensis* demonstrated good preliminary safety within the 50–100 mg/kg dose range during the 21-day observation period.

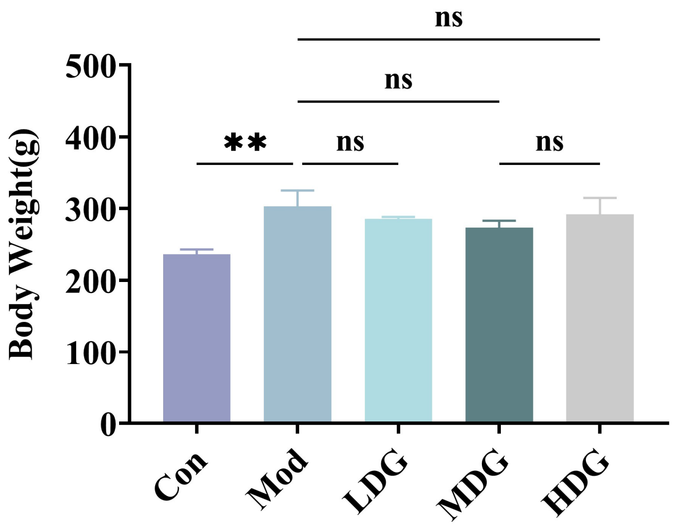

Fig.S10 Comparison of body weight changes among different groups of rats on the 22nd day.

In preliminary experiments, the 50 mg/kg dose demonstrated significant therapeutic effects, and there was no significant difference in efficacy compared to the 100 mg/kg dose. Based on literature reports regarding

the optimal concentration of *baicalin* for targeting the TLR4 pathway (9), we considered 50 mg/kg to be the preferred dose, as this dose strikes the best balance between efficacy and safety.

(3) Discussion of dose-dependent effects:

Given that using a single dose in formal experiments is insufficient to fully elucidate the dose-response relationship, we have added an analysis of this limitation to the "Experimental Design" section of the revised manuscript (line 216). Preliminary experiments suggested that the 50 mg/kg dose of *Scutellaria baicalensis* may be approaching the plateau phase of its effect; however, dose-gradient experiments ranging from 25 to 100 mg/kg are still required to determine the optimal dose for improving reproductive endocrine disorders in PCOS rats. The design and results of the relevant preliminary experiments are summarized in the "Supplementary Materials."

2. Was the dose for the metform control group (270mg/Kg/day) determined with reference to the body surface area convertion standard between rats and humans? Is this dose supported by previous literature regarding its efficacy in PCOS rat models?

Answer: Thank you for your valuable feedback. Your questions regarding the basis for metformin dosing and the relevant literature are of critical importance. We provide a detailed explanation below:

(1) Explanation of Dosage Conversion Standards:

Yes, the dosage of the metformin control group (270 mg/kg/day) in this study was determined in strict accordance with the body surface area-based dose conversion standards for rats and humans.

The logic and basis for this conversion are as follows:

Clinical dose reference: The commonly used daily dose range for metformin in the treatment of PCOS in humans is 1500–2000 mg/day/person.

Body Surface Area Conversion: Human clinical doses are converted to rat equivalent doses using interspecies dose conversion formulas based on body surface area and metabolic rate. A commonly used empirical formula is: human dose (mg/kg) × 6.2 ≈ rat equivalent dose (mg/kg); a direct conversion method based on body surface area may also be used.

Dose calculation: Taking a 60kg human as an example, the clinical dose of 25 mg/kg/day (1,500 mg/day) corresponds to an equivalent dose in rats of approximately 155 mg/kg/day; a dose of 33.3 mg/kg/day (2,000

mg/day) corresponds to more than 200 mg/kg/day. Therefore, the high doses of 250–300 mg/kg/day reported in the literature for rats ensure significant metabolic and endocrine improvements, a range that corresponds to moderate-to-high clinical doses in humans.

Conclusion: The selected dose of 270 mg/kg/day falls within the generally accepted range of effective doses and is intended to simulate and validate the pharmacological effects of metformin in the clinical treatment of PCOS; this dose selection is reasonable.

(2) Literature supporting the efficacy of this dose in a rat model of PCOS:

The literatures indicated that metformin, at a dose of 250–300 mg/kg/day, can significantly improve reproductive endocrine and metabolic disorders in a rat model of PCOS, including reducing serum testosterone(T) and luteinizing hormone(LH) levels, restoring the estrous cycle, improving insulin resistance, and alleviating ovarian morphological abnormalities (such as reducing the number of cystic follicles and promoting corpus luteum formation). For example, Yuan Xie et al(10) administered high (250 mg/kg/day), medium (50 mg/kg/day), and low (10 mg/kg/day) doses of metformin and found that the high dose effectively alleviated hepatic lipid accumulation in mice with PCOS and improved mitochondrial dysfunction and redox imbalance. Another study confirmed that 300 mg/kg/day of metformin significantly improved body mass index, insulin resistance, lipid profile, sex hormone levels, inflammatory markers, and polycystic-like ovarian changes in PCOS rats (11). Furthermore, metformin at 300 mg/kg/day restored the estrous cycle in PCOS rats, suppressed weight gain, and reduced testosterone levels (12).

In summary, the metformin dose of 270 mg/kg/day used in this study was calculated based on cross-species conversion using body surface area to simulate a clinically effective dose in humans. Previous studies have demonstrated that this dose significantly improves estrous cycles, endocrine parameters, and metabolic indicators in a rat model of PCOS. The selection of this control dose is well-founded scientifically and ensures the comparability of the study.

3. Molecular docking was performed with only three core active compounds (wogonin, baicalein, and oroxylin A) and three targets (TNF, IL6, AKT1) ,without explaining why other potential targets were excluded. Is the rationale for target selection sufficiently justified?

Answer: We would like to thank you for your valuable comments. Regarding the rationale for selecting three core active compounds (*wogonin, baicalein*, and *oroxylin A*) and three targets (TNF, IL-6, and AKT1)

in our molecular docking study, while excluding other potential targets, we provide the following explanation:

(1) Rationale and Basis for Target Selection

This study employed an iterative approach, utilizing "component-target" networks and protein-protein interaction network analysis, and screening based on degree and centrality, to ultimately identify TNF, IL-6, and AKT1 as core targets. All three are key regulators of inflammatory and insulin signaling pathways and have been identified as hub genes in network pharmacology studies of PCOS. Previous research has shown that YJKL may improve PCOS-related infertility by regulating the TNF signaling pathway, further validating the central roles of AKT1 and TNF (13); another study on Morus alba branches similarly validated the critical role of AKT1 and TNF in PCOS treatment (14). Furthermore, the Wen Shen Hua Tan Qu Yu Decoction has been shown to potentially improve PCOS-related inflammation and apoptosis by regulating IL-6 expression (15). The aforementioned targets are clearly associated with the core pathological mechanisms of PCOS—chronic inflammation, insulin resistance, and ovarian dysfunction—and the underlying mechanisms are well-supported by the literature (16, 17). Currently, the strategy of screening TNF, IL-6, and AKT1 as representative targets based on PPI networks has been adopted in multiple studies incorporating molecular docking, indicating that the target selection method used in this study aligns with mainstream research paradigms in the field (18-20).

(2) The Scientific Basis for Screening Bioactive Compounds

This study selected three flavonoid compounds—*wogonin, baicalein*, and *oroxylin A*—which are abundant in *Scutellaria baicalensis* and exhibit significant biological activity, as the core ligand molecules. These compounds constitute the primary molecular basis for the pharmacological activity of *Scutellaria baicalensis* (21); These compounds possess anti-inflammatory and metabolic regulatory effects and are closely associated with the functions of TNF, IL-6, and AKT1. Previous studies have demonstrated that they exert their biological effects by regulating TNF-α, IL-6, and the PI3K-AKT pathway (22, 23). Based on this, this study uses three bioactive compounds as ligands to validate molecular docking against three key targets. Adhering to the principles of "simplicity and precision," it evaluates the core "compound-target" interactions predicted by network pharmacology at minimal cost.

(3) Limitations of the Study and Future Directions

We thank the reviewer for your constructive feedback. In addition to TNF, IL-6, and AKT1, other targets may also mediate the multi-target effects of *Scutellaria baicalensis*. Given that this study represented an initial exploration of the underlying mechanisms, we prioritized the validation of core targets that exhibited the most significant network topological features and the clearest association with PCOS pathology.

To address the limitations of this study, we have added the following to line 584 of the "Discussion" section: In future studies, we will combine high-throughput technologies such as network pharmacology and proteomics to systematically identify the potential target profiles of the bioactive components in *Scutellaria baicalensis*, and validate their functions through gene silencing and overexpression to elucidate the multi-target synergistic mechanism underlying its treatment of PCOS.

Thank you once again for your review and constructive feedback. The comments mentioned above have been incorporated into the "Materials and Methods" and "Discussion" sections of the supplementary materials, and the relevant references have been noted in the text, Please check.

4. The in vivo metabolic fate of the active compounds from Scutellaria baicalensis remains unclear. Were the concentrations of these compounds measured in serum, ovarian, or intestinal tissues to confirm their ability to reach the target sites and achieve effective concentrations? Were potential synergistic or antagonistic interactions among the compounds considered?

Answer: We sincerely thank you for your valuable feedback, which has provided important guidance for enhancing the scientific rigor of this paper. In response to your questions regarding the in vivo metabolic pathways, distribution in target tissues, and interactions of the active components of *Scutellaria baicalensis*, we provide the following responses:

(1) Determination of the distribution and concentration of active ingredients in target tissues

We fully agree with the reviewer's comments: active compounds must reach the target organs and achieve effective concentrations to elucidate their pharmacological mechanisms. We did not measure the concentrations of *Scutellaria baicalensis's* active compounds in serum, ovaries, or the intestines, which constituted a limitation of this study. Our focus was on predicting potential targets and pathways for *Scutellaria baicalensis* in the treatment of PCOS through network pharmacology, combined with validation via animal experiments. In future work, we will establish a quantitative method using LC-MS/MS to determine the pharmacokinetic profiles of *Scutellaria baicalensis's* major active components in serum and target organs. We will conduct PK/PD analyses in conjunction with pharmacodynamic studies to confirm

effective concentrations at target sites and establish the relationship between network predictions and in vivo pharmacodynamics and metabolism.

(2) Regarding synergistic or antagonistic interactions between compounds

The reviewer's insights into compound interactions are profound, and this is precisely the core of research on the complex systems of traditional Chinese medicine. The various flavonoid components in *Scutellaria baicalensis* may exert their pharmacological effects through complex interactions. In this study, we treated the multi-component mixture of *Scutellaria baicalensis* as a single entity and constructed a "multi-component–multi-target" network. Although this approach initially accounted for synergistic effects, it did not involve in-depth experimental analysis of the synergistic or antagonistic interactions among the individual components.

According to the recommendations, future research could combine the Chou-Talalay composite index method with animal efficacy indicators (such as hormone levels, follicular development, and glucose metabolism) to optimize the ratio of active components in *Scutellaria baicalensis*, thereby shifting the focus from "holistic prediction" to "compositional optimization" to elucidate its mechanism of action in treating PCOS.

Thank you once again for your constructive feedback. We have addressed the limitations of the study in line 661 of the manuscript's conclusion and will address them in future research.

5. The intervention period lasted merely 5 weeks. No assessments were performed on long-term outcomes (e.g., the recovery of fertility in rats, offspring growth) or long-term safety (e.g., hepatic and renal function, sustained remodeling of the gut microbiota). Can the results of short-term intervention adequately reflect the efficacy of long-term clinical administration?

Answer: We appreciate your constructive feedback. We fully acknowledge that the main limitation of this study is that the intervention lasted only five weeks and did not assess long-term efficacy and safety; we will address this in future studies.

In response to the reviewers' questions, we address them from three perspectives: the rationale behind the experimental design, the significance of our key findings, and future directions:

(1)Based on pathological evaluations and preliminary observations in rats with PCOS, this study employed a 5-week intervention protocol; the duration of this protocol was determined by reference to the research

paradigm of previous studies exploring the mechanisms of traditional Chinese medicine in treating PCOS through the gut microbiota (24, 25), Studies of this type typically employ a 4–8-week treatment period. Our preliminary experiments indicated that a 3-week intervention with *Scutellaria baicalensis* can produce initial improvements in the phenotype of rats with PCOS (slowed weight gain, decreased serum testosterone levels, and reduced estrous cycle irregularities). Based on these findings, and to ensure the detection of reproductive endocrine effects mediated by gut microbiota regulation as well as the statistical significance of the outcome measures, the treatment period for this study was ultimately set at 5 weeks.

(2)This study found that *Phocaeicola* is a key gut microbiota species involved in *Scutellaria baicalensis's* regulation of PCOS. Short-term interventions can identify contributing bacterial communities but cannot explain the mechanisms underlying the sustained effects. After a 5-week intervention, this bacterium was significantly enriched and strongly correlated with host phenotypes, suggesting that *Phocaeicola* may act as a key microbial mediator in the long-term therapeutic effects of *Scutellaria baicalensis*. This finding provided targets and a theoretical basis for subsequent mechanistic studies (colony-forming ability, metabolite profiles, and host gene expression regulation).

(3)This study is an exploratory investigation aimed at elucidating the early mechanisms by which *Scutellaria baicalensis* regulated PCOS via the "gut-gonadal axis"; it was not intended to simulate long-term clinical use. Regarding the reviewer's concern about long-term safety, we have addressed this in line 643 of the "Discussion" section. In future studies, we plan to extend the treatment period to more than 10 weeks, focusing on three aspects: (a) The colonization dynamics of *Phocaeicola* and the recovery capacity of the gut microbiota following drug discontinuation; (b) Rat fertility and the development of offspring; (c) Histopathology of liver and kidney tissues and serum biochemical markers. We hope to more systematically evaluate the long-term efficacy and safety of *Scutellaria baicalensis* in treating PCOS through the regulation of the gut microbiota.

We would like to once again express our gratitude to the reviewer for your valuable suggestions. These comments will help enhance the scientific rigor of this study and provide important guidance for future long-term research.

6. There are significant differences in the composition of the gut microbiota between humans and rats. In the present study, is there any supporting literature in human patients with polycystic ovary syndrome (PCOS) for the regulatory effects of Scutellaria baicalensis on the abundance of Phocaeicola? How can the translational gaps between animal models and clinical applications in humans be addressed?

Answer: We would like to thank you for your valuable comments. Regarding the differences in gut microbiota composition between humans and rats, as well as the issue of evidence for the genus *Phocaeicola* in clinical studies of PCOS, our response is as follows:

(1)We agree with the reviewer that differences in gut microbiota composition between rodents and humans pose a common challenge for microbiota-related translational research. As the reviewer noted, there are currently no clinical studies reporting the effects of *Scutellaria baicalensis* on the abundance of the genus *Phocaeicola* in patients with PCOS. The reasons are as follows: First, the genus *Phocaeicola* was only recently established as a new genus distinct from *Bacteroides*, and its role in the pathophysiology of PCOS has not yet received widespread attention in clinical studies; second, research on the mechanisms of traditional Chinese medicine based on the gut microbiota is largely still in the preclinical stage.

Nevertheless, the primary value of this study lies in providing targets and theoretical hypotheses for translational research. The genera *Phocaeicola* and *Bacteroides* are closely related and perform similar functions in glycolipid metabolism and host-microbial coevolution. Previous studies have shown that the primary active components of *Scutellaria baicalensis* (such as *baicalin*) can improve metabolic disorders by regulating the abundance of *Bacteroides* in the gut (26, 27), providing indirect support for the findings of this study.

(2)To bridge the translational gap between animal models and clinical applications, we plan to proceed with the following three areas of work:

First, we plan to use metagenomic sequencing to compare the similarity between *Phocaeicola* strains derived from rats and humans in terms of core functional genes (such as carbohydrate metabolism enzymes and short-chain fatty acid synthesis pathways), in order to assess the functional conservation of these genes in metabolic regulation.

Second, we plan to establish a humanized PCOS mouse model (by transplanting patient fecal microbiota into germ-free mice) to verify, within this human microbiome context, the role of *Scutellaria baicalensis* in enriching the genus *Phocaeicola* and its effects on improving reproductive endocrine phenotypes, thereby further clarifying the causal relationship between them.

Third, we plan to conduct a clinical cohort study. We are currently designing an exploratory clinical trial for patients with PCOS. The study aims to assess the effect of *Scutellaria baicalensis* on the abundance of the genus *Phocaeicola* in the gut using 16S rRNA sequencing and metagenomic analysis. We will also perform

association analyses by integrating data on sex hormone levels, markers of glucose and lipid metabolism, and the restoration of the estrous cycle, with the goal of providing direct evidence for clinical translation.

In summary, the contribution of this study lies in revealing that *Phocaeicola* may act as a key microorganism in mediating the regulatory effects of *Scutellaria baicalensis* on the "gut-ovarian axis."

7. The study indicates that the genus Phocaeicola is a core gut microbiota component regulated by Scutellaria baicalensis, but its specific mechanism of action remains unclear. Does Phocaeicola contribute to intestinal barrier repair by producing metabolites such as short-chain fatty acids or bile acids? Or does it directly interact with intestinal epithelial cells to inhibit LPS release?

Answer: Thank you for your valuable suggestions regarding the mechanistic analysis. We fully agree that it is crucial to elucidate the specific mechanisms by which *Phocaeicola* modulates host metabolism and inflammatory states under *Scutellaria baicalensis* intervention. It should be noted that this study primarily identified *Phocaeicola* as a key biomarker based on metagenomic analysis; fecal microbiota transplantation experiments have not yet been conducted, so we are currently unable to directly establish a causal relationship between *Phocaeicola* and the observed phenotypes.

Both mechanisms proposed by the reviewer—that *Phocaeicola* indirectly regulated the intestinal barrier via metabolites, or directly interacted with the intestinal epithelium to inhibit LPS release—are of significant value. This study found that *Scutellaria baicalensis* upregulated tight junction proteins, inhibited LPS release, and suppressed *TLR4/NF-κB* pathway activation, thereby directly affected the intestinal epithelium. These findings suggested that *Scutellaria baicalensis* may exert its effects through dual regulation of the "microbiota-intestinal epithelium" axis, with *Phocaeicola* potentially played a synergistic or mediating role in this process.

Based on the above results, we hypothesize that the mechanism of action of *Phocaeicola* may involve two modes (or a combination of both): (a) indirect regulation of the intestinal epithelial barrier via metabolites, synergizing with the inhibition of the *TLR4/NF-κB* pathway by *Scutellaria baicalensis*; (b) acting as a core responsive bacterial genus, altering its interaction pattern with intestinal epithelial cells under the influence of *Scutellaria baicalensis*, thereby affecting LPS translocation and the host inflammatory response.

Future research will be conducted through the following experiments: (a) Combining metagenomics with targeted metabolomics to analyze the levels of short-chain fatty acids, bile acid metabolism-related genes, and metabolites in *Phocaeicola*; (b) Using germ-free or pseudo-germ-free mouse models for monomicrobial

colonization, combined with *Scutellaria baicalensis* intervention, to elucidate the causal role of this bacterium in regulating the intestinal barrier and inflammatory pathways; (c) Establishing a *Phocaeicola-Caco-2* cell co-culture system to observe its direct regulatory effects on tight junction proteins, LPS translocation, and the *TLR4/NF-κB* pathway.

In summary, although this study did not establish a causal role for *Phocaeicola* through fecal transplantation, it did confirm that *Scutellaria baicalensis* directly modulated the intestinal barrier and inflammatory pathways. The reviewer accurately identified gaps in our understanding of the underlying mechanisms. The experiments mentioned above will be incorporated into our future plans. We would like to once again thank the reviewer for your valuable feedback.

8. In microbiome analysis,when no significant differences were observed in α - diversity (Shannon and Simpson indices) but distinst significant defferences existed in β - diversity, how to interpret the phenomenon of " unchanged species richness yet remodeling of microbial community structure"? Could this be attributed to the selective regulation of specific functional microbial taxa by Scutellaria baicalensis?

Answer: Thank you for your insightful questions regarding the analysis of microbiome data. In this study, there were no significant changes in *α-diversity* (*Shannon index* and *Simpson's index*) following the *Scutellaria baicalensis* intervention, indicating that species richness and evenness were not significantly affected; however, *β-diversity* was significantly altered, suggesting that *Scutellaria baicalensis* induced an overall restructuring of the microbial community. This is commonly observed when an intervention selectively modulates specific microbial groups rather than broadly affecting overall diversity.

We believe this phenomenon stems from *Scutellaria baicalensis's* selective regulation of specific functional microbial communities. Differential analysis and LEfSe analysis identified the genus *Phocaeicola* as a biomarker; changes in its abundance were closely associated with improvements in host phenotype, and there were no significant differences in *α-diversity*, indicating that *Scutellaria baicalensis* exerts a functionally targeted regulatory effect on the microbiota.

In summary, the stability of *α-diversity* and the significant changes in *β-diversity* suggest that *Scutellaria baicalensis* maintained the overall richness of the microbial community while selectively reshaping key functional genera (such as *Phocaeicola*), thereby synergistically improving the host's pathological condition. Once again, we sincerely thank you for your valuable feedback.

1. Armanini D, Boscaro M, Bordin L, Sabbadin C. 2022. Controversies in the Pathogenesis, Diagnosis and Treatment of PCOS: Focus on Insulin Resistance, Inflammation, and Hyperandrogenism. International Journal of Molecular Sciences 23.
2. Rudnicka E, Suchta K, Grymowicz M, Calik-Ksepka A, Smolarczyk K, Duszewska AM, Smolarczyk R, Meczekalski B. 2021. Chronic Low Grade Inflammation in Pathogenesis of PCOS. International Journal of Molecular Sciences 22.
3. Juda A, Rojek K, Kamińska M, Strzoda AJ, Strzoda A, Sowiński WJ, Zdybel M, Strzoda A. 2025. Metformin in the Treatment of Polycystic Ovary Syndrome: A Literature Review. International Journal of Infertility & Fetal Medicine 16:186-190.
4. Teede HJ, Tay CT, Laven JJE, Dokras A, Moran LJ, Piltonen TT, Costello MF, Boivin J, Redman LM, Boyle JA, Norman RJ, Mousa A, Joham AE, Arlt W, Azziz R, Balen A, Bedson L, Berry L, Boivin J, Boyle J, Brennan L, Brown W, Burgert T, Busby M, Ee C, Garad RM, Gibson-Helm M, Harrison C, Hart R, Hopkins K, Hirschberg AL, Ho T, Hoeger K, Jordan C, Legro RS, Li R, Lujan M, Ma R, Mansfield D, Marsh K, Mocanu E, Mol B, Mormon R, Norman R, Oberfield S, Patel M, Pattuwage L, Peña A, Redman L, Rombauts L, et al. 2023. Recommendations from the 2023 international evidence-based guideline for the assessment and management of polycystic ovary syndrome. European Journal of Endocrinology 189:G43-G64.
5. Li P, Shuai P, Shen S, Zheng H, Sun P, Zhang R, Lan S, Lan Z, Jayawardana T, Yang Y, Zhao J, Liu Y, Chen X, El-Omar EM, Wan Z. 2023. Perturbations in gut microbiota composition in patients with polycystic ovary syndrome: a systematic review and meta-analysis. BMC Medicine 21.
6. Miller CB, Bader GA, Kay CL. 2026. Fecal Microbiota Transplantation in 2025: Two Steps Forward, One Step Back. Current Gastroenterology Reports 28.
7. Xu X, Xu X, Wang X, Shen L. 2023. Baicalin suppress the development of polycystic ovary syndrome via regulating the miR-874-3p/FOXO3 and miR-144/FOXO1 axis. Pharm Biol 61:878-885.
8. Li YY, Peng YQ, Yang YX, Shi TJ, Liu RX, Luan YY, Yin CH. 2024. Baicalein improves the symptoms of polycystic ovary syndrome by mitigating oxidative stress and ferroptosis in the ovary and gravid placenta. Phytomedicine 128:155423.
9. Shah M-A, Park D-J, Kang J-B, Kim M-O, Koh P-O. 2019. Baicalin attenuates lipopolysaccharide-induced neuroinflammation in cerebral cortex of mice via inhibiting nuclear factor kappa B (NF-κB) activation. Journal of Veterinary Medical Science 81:1359-1367.
10. Xie Y, Tian Y, Huang J, Deng W, Li X, Liu Y, Liu H, Gao L, Xie Q, Yu Q. 2025. Metformin Alleviates Liver Metabolic Dysfunction in Polycystic Ovary Syndrome by Activating the Ethe1/Keap1/PINK1 Pathway. International Journal of Biological Sciences 21:3505-3526.
11. Mahmoud MM, Rashed LA, Soliman SA, Sayed SM, Kamel O, Kamar SS, Hussien RES. 2023. SGLT-2 inhibitors enhance the effect of metformin to ameliorate hormonal changes and inflammatory markers in a rat PCOS model. Physiological Reports 11.
12. Tohma Y, Onalan G, Tepeoglu M, Bayraktar N, Colak E, Ozcimen E, Zeyneloglu H. 2019. Phosphodiesterase 4 inhibitor plus metformin is superior to metformin alone for the treatment of polycystic ovary syndrome: A rat model study. Experimental and Therapeutic Medicine doi:10.3892/etm.2019.7428.
13. Zhang R, Xu W, Wei H, Li B, Wang Y, He X, Cao J, He X, Xu M, Lu W, Xu Y. 2024. Mechanism of YJKL Decoction in Treating of PCOS Infertility by Integrative Approach of Network Pharmacology and Experimental Verification. Drug Design, Development and Therapy Volume 18:3853-3870.
14. Jiang Q, Pu D, Hu Y, Wu R, Jiang C, Yuan S, Zhu X, Xu L. 2025. Network pharmacology-based investigation of potential targets of mulberry twig acting on polycystic ovary syndrome. Medicine 104.
15. Guo X, Wang Q, Zhong Z. 2025. Key molecules in Wenshen Huatan Quyu Decoction for polycystic ovary syndrome: Insights from network pharmacology. Asian Journal of Surgery 48:2613-2614.
16. Xu W, Tang M, Wang J, Wang L, Kennedy DA. 2020. Identification of the Active Constituents and Significant Pathways of Cangfu Daotan Decoction for the Treatment of PCOS Based on Network Pharmacology. Evidence-Based Complementary and Alternative Medicine 2020.
17. Ying Z, Hongqiu Z, Junjie L, Peijuan W, qian Z, Mengxue D, Yan L. 2026. Network pharmacology and molecular docking reveal the mechanism of Zishen Yutai Wan against polycystic ovary syndrome. Journal of Ovarian Research 19.

18. Ding J, Shanshan M, Mengcheng C, Danying Z, Jin Y. 2022. Integrated Network Pharmacology and Clinical Study to Reveal the Effects and Mechanisms of Bushen Huoxue Huatan Decoction on Polycystic Ovary Syndrome. Evid Based Complement Alternat Med 2022:2635375.
19. Hai B, Zhang Y, Huang J, Mprah R, Wang M. 2025. Exploring the key ingredients and mechanisms of Banxia Xiexin decoction for the treatment of polycystic ovary syndrome based on network pharmacology and experimental validation. Annals of Medicine 57.
20. Liu Y, Bai H, Guan H, Wang C, Song X, Yong Z, Guo X, Li L, Zhang Z. 2025. Animal experiments and network pharmacology to explore the anti-inflammatory mechanism of dapagliflozin in the treatment of polycystic ovary syndrome. Gynecological Endocrinology 41.
21. Ji Y, Han J, Lee N, Yoon J-H, Youn K, Ha HJ, Yoon E, Kim DH, Jun M. 2020. Neuroprotective Effects of Baicalein, Wogonin, and Oroxylin A on Amyloid Beta-Induced Toxicity via NF-κB/MAPK Pathway Modulation. Molecules 25.
22. Zhang L, Tian Y, Wang J, Deng S, Fan H. 2023. Network pharmacology-based research on the effect of Scutellaria baicalensis on osteosarcoma and the underlying mechanism. Medicine 102.
23. Zhu G, Zhang J, Yang Y, Zhang H, Jin W, Su F, Liang J, Wang K, Zhang J, Chen C. 2021. The Key Target and Molecular Mechanism of the Volatile Component of Scutellaria baicalensis Georgi in Acute Lung Injury Based on Network Pharmacology. Frontiers in Pharmacology 12.
24. Gao Y, Mo S, Cao H, Zhi Y, Ma X, Huang Z, Li B, Wu J, Zhang K, Jin L. 2023. The efficacy and mechanism of Angelica sinensis (Oliv.) Diels root aqueous extract based on RNA sequencing and 16S rDNA sequencing in alleviating polycystic ovary syndrome. Phytomedicine 120:155013.
25. Wang Y, Xiao H, Liu Y, Tong Q, Yu Y, Qi B, Bu X, Pan T, Xing Y. 2022. Effects of Bu Shen Hua Zhuo formula on the LPS/TLR4 pathway and gut microbiota in rats with letrozole-induced polycystic ovary syndrome. Front Endocrinol (Lausanne) 13:891297.
26. Lin Y, Wang ZY, Wang MJ, Jiang ZM, Qin YQ, Huang TQ, Song Y, Liang HT, Liu EH. 2022. Baicalin attenuate diet-induced metabolic syndrome by improving abnormal metabolism and gut microbiota. European Journal of Pharmacology: An International Journal:925.
27. Wang J, Ishfaq M, Li J. 2021. Baicalin ameliorates Mycoplasma gallisepticum-induced inflammatory injury in the chicken lung through regulating the intestinal microbiota and phenylalanine metabolism. Food & Function.

Re: mSystems01825-25R1 (Targeting the Gut-Ovarian Axis: Scutellaria baicalensis Improves Polycystic Ovary Syndrome by Modulating Gut Microbiota Composition and Inhibiting the LPS/TLR4/NF-κB Signaling Pathway)

Dear Dr. Zhengxiu Pan:

Your manuscript has been accepted, and I am forwarding it to the ASM production staff for publication. Your paper will first be checked to make sure all elements meet the technical requirements. ASM staff will contact you if anything needs to be revised before copyediting and production can begin. Otherwise, you will be notified when your proofs are ready to be viewed.

Data Availability: ASM policy requires that data be available to the public upon online posting of the article, so please verify all links to sequence records, if present, and make sure that each number retrieves the full record of the data. If a new accession number is not linked or a link is broken, provide production staff with the correct URL for the record. If the accession numbers for new data are not publicly accessible before the expected online posting of the article, publication may be delayed; please contact ASM production staff immediately with the expected release date.

Publication Fees: For information on publication fees and which article types have charges, please visit our website. We have partnered with Copyright Clearance Center (CCC) to collect author charges. If fees apply to your paper, you will receive a message from no-reply@copyright.com with further instructions. For questions related to paying charges through RightsLink, please contact CCC at ASM_Support@copyright.com or toll free at +1-877-622-5543. CCC makes every attempt to respond to all emails within 24 hours.

ASM Membership: Corresponding authors may join or renew ASM membership to obtain discounts on publication fees. Need to upgrade your membership level? Please contact Customer Service at Service@asmusa.org.

Embargo Policy: A press release may be issued as soon as the manuscript is posted on the mSystems Latest Articles webpage. The corresponding author will receive an email with the subject line "ASM Journals Author Services Notification" when the article is available online.

Cover Image Submissions: If you would like to submit a potential Cover Image, please email a file and a short legend to msystems@asmusa.org. Please note that we can only consider images that (i) the authors created or own and (ii) have not been previously published. By submitting, you agree that the image can be used under the same terms as the published article. Image File requirements: TIF/EPS, 7.5 inches wide by 8.25 inches tall (at least 2,250 pixels wide by 2,475 pixels tall), minimum 300 dpi resolution (600 dpi preferred), RGB, and no figure elements, e.g., arrows or panel labels. The legend should be a short description of the image, 1-2 sentences recommended. Please download and use this interactive template in Adobe to ensure that your proposed cover image meets our size requirements (https://journals.asm.org/pb-assets/pdf-text-excel-files/ASM-Interactive-Sizing-Cover-Template-1715689791.pdf).

Thank you for submitting your paper to mSystems.

Sincerely,
Sangram Bagh
Editor
mSystems

Reviewer #1 (Comments for the Author):

The authors have adequately responded to all of my comments and made appropriate revisions.

Reviewer #2 (Comments for the Author):

The authors have thoughtfully addressed all previous concerns raised during the review process, enhancing the overall quality and validity of the research.
